# Autonomous Synthesis of Functional, Permanently Phosphorylated Proteins for Defining the Interactome of Monomeric 14-3-3ζ

**DOI:** 10.1021/acscentsci.3c00191

**Published:** 2023-04-10

**Authors:** Phillip Zhu, Stanislau Stanisheuski, Rachel Franklin, Amber Vogel, Cat Hoang Vesely, Patrick Reardon, Nikolai N. Sluchanko, Joseph S. Beckman, P. Andrew Karplus, Ryan A. Mehl, Richard B. Cooley

**Affiliations:** † Department of Biochemistry and Biophysics, Oregon State University, 2011 Agricultural and Life Sciences, Corvallis, Oregon 97331, United States; ‡ Department of Chemistry, Oregon State University, 153 Gilbert Hall, Corvallis, Oregon 97331, United States; § e-MSion Inc., 2121 NE Jack London St., Corvallis, Oregon 97330, United States; ∥ A.N. Bach Institute of Biochemistry, Federal Research Center of Biotechnology of the Russian Academy of Sciences, 119071 Moscow, Russia

## Abstract

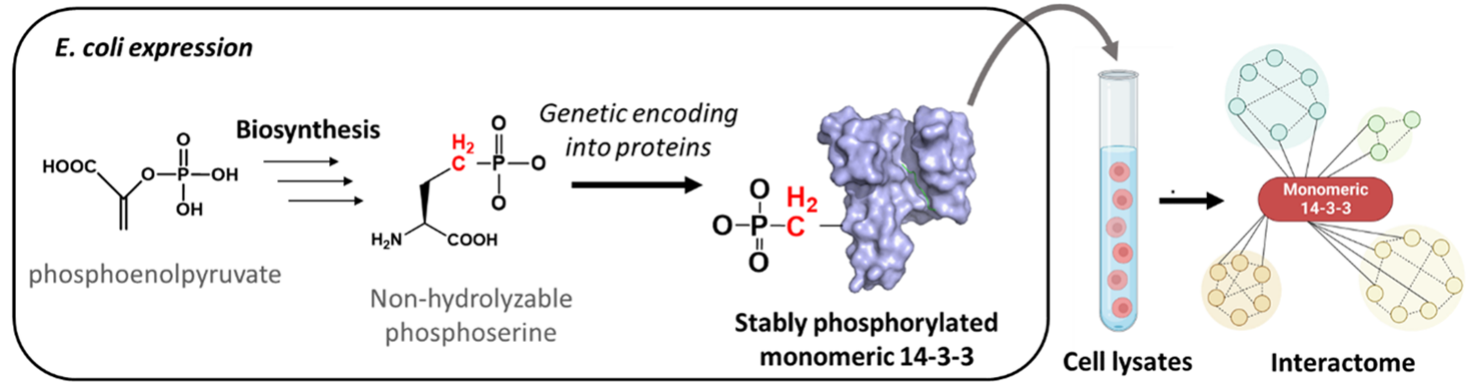

14-3-3 proteins are dimeric hubs that bind hundreds of phosphorylated “clients” to regulate their function. Installing stable, functional mimics of phosphorylated amino acids into proteins offers a powerful strategy to study 14-3-3 function in cellular-like environments, but a previous genetic code expansion (GCE) system to translationally install nonhydrolyzable phosphoserine (nhpSer), with the γ-oxygen replaced with CH_2_, site-specifically into proteins has seen limited usage. Here, we achieve a 40-fold improvement in this system by engineering into *Escherichia coli* a six-step biosynthetic pathway that produces nhpSer from phosphoenolpyruvate. Using this autonomous “PermaPhos” expression system, we produce three biologically relevant proteins with nhpSer and confirm that nhpSer mimics the effects of phosphoserine for activating GSK3β phosphorylation of the SARS-CoV-2 nucleocapsid protein, promoting 14-3-3/client complexation, and monomerizing 14-3-3 dimers. Then, to understand the biological function of these phosphorylated 14-3-3ζ monomers (containing nhpSer at Ser58), we isolate its interactome from HEK293T lysates and compare it with that of wild-type 14-3-3ζ. These data identify two new subsets of 14-3-3 client proteins: (i) those that selectively bind dimeric 14-3-3ζ and (ii) those that selectively bind monomeric 14-3-3ζ. We discover that monomeric—but not dimeric—14-3-3ζ interacts with cereblon, an E3 ubiquitin-ligase adaptor protein of pharmacological interest.

## Abstract 2

An autonomous *E. coli* system to genetically encode a functional nonhydrolyzable phosphoserine mimic into proteins is created and used to reveal how 14-3-3 phosphorylation rewires its interactome.

## Introduction

In humans, over 75% of proteins are reversibly phosphorylated at one or more sites, with nearly 80% of these being phosphoserine (pSer) modifications.^1^ An essential eukaryotic protein family that regulates phospho-protein function is called 14-3-3, for which seven isoforms of 14-3-3 proteins exist in humans (denoted by their Greek letter β, γ, ε, ζ, η, θ, σ), and all are natively dimeric.^2−4^ They act as central hubs that bind hundreds of “client” proteins when clients are phosphorylated at serine/threonine sites within specific sequence motifs as a means to regulate client activity, cellular localization and ability to interact with other proteins.^5−7^ Though 14-3-3 proteins have been the subject of intense investigation, studying the molecular mechanisms by which they regulate client function remains a challenge due to difficulties in making 14-3-3/phospho-client complexes, as well as their transient nature in a cellular context where phosphatases can hydrolyze clients forcing them to disassociate from 14-3-3.^8^ Adding to these challenges is that 14-3-3 *itself* is subject to many post-translational modifications (PTMs) that are thought to be important for regulating 14-3-3 function,^7^ including at least 20 sites of phosphorylation.^9^

One key site of phosphorylation on 14-3-3 is Ser58 (ζ-isoform numbering, a conserved site in all isoforms except θ and σ). Phosphorylation at this site primes cells for apoptosis by monomerizing 14-3-3,^10−12^ creating interest in anticancer therapeutics that act to stabilize the monomeric form.^13^ Efforts to study this phosphorylated form of 14-3-3 by direct enzymatic phosphorylation of the 14-3-3 dimer at this interfacial residue proved ineffective.^14−16^ Prior work to understand the role of Ser58 phosphorylation therefore has largely focused on using phosphomimicking mutations (S → D/E), which unfortunately only partially monomerize 14-3-3,^17−20^ as well as a variety of mutated forms of 14-3-3 that achieve monomerization by including multiple mutations (up to 7) at the dimer interface.^21^ It remains unclear how the monomerization of 14-3-3 by phosphorylation, as it occurs inside the cell, alters its interactome and ability to regulate client function in a way that primes cells for apoptosis.

**Figure 1:**
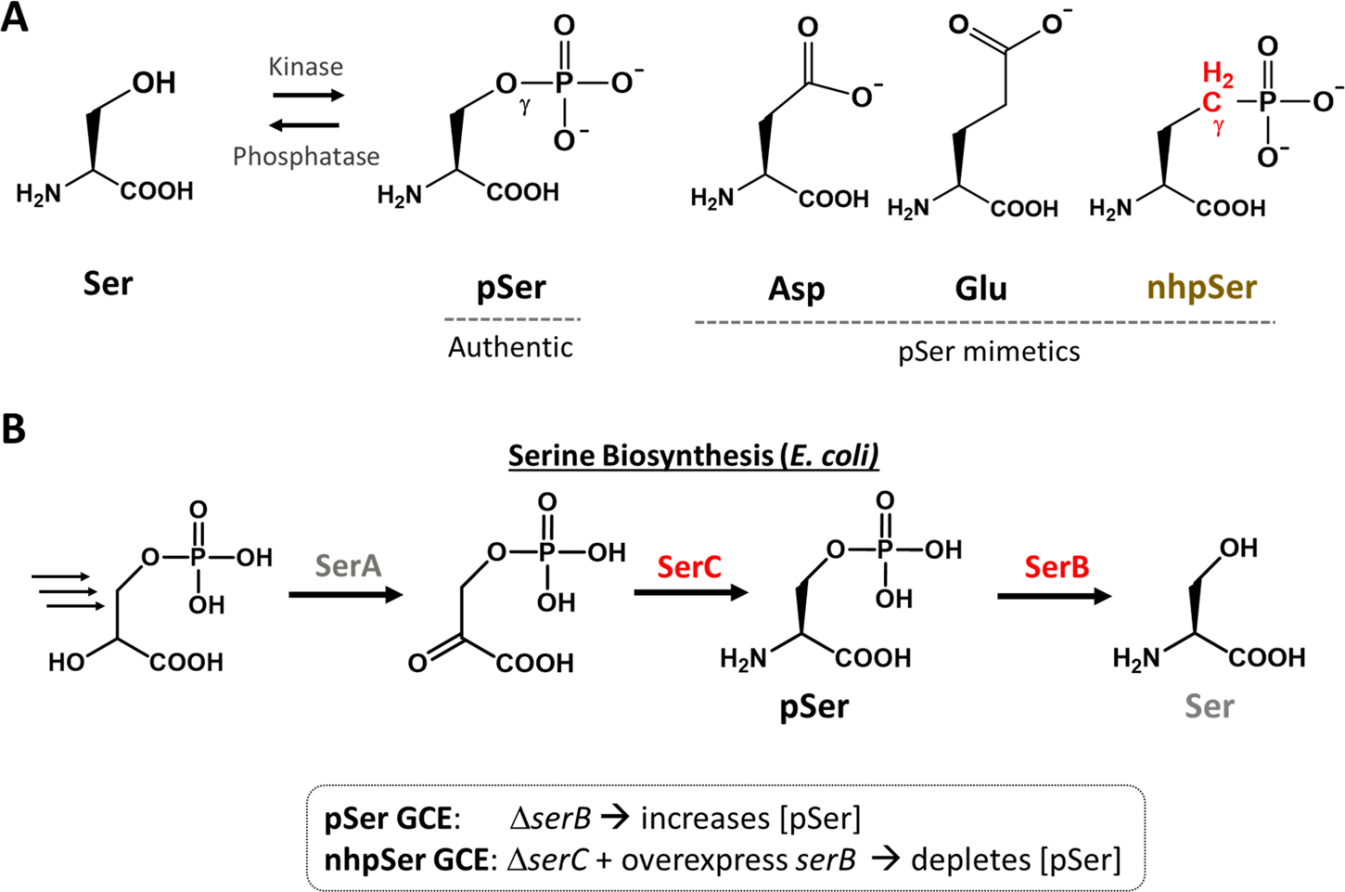
Mimics and biosynthesis of phosphoserine. (A) Structures of phosphoserine and its mimics. (B) Phosphoserine is a biosynthetic intermediate of serine in *E. coli*. This pathway can be altered for GCE purposes to elevate (for pSer GCE) or eliminate (for nhpSer GCE) intracellular concentrations of the free phosphoserine amino acid. Residual pSer produced by transaminases other than SerC can be reduced further by overexpression of *serB* in *serC* knockout strains.

We and others have shown that genetic code expansion (GCE) offers a tractable strategy to overcome challenges associated with making site-specifically phosphorylated client proteins, whereby phosphoserine is incorporated into proteins during translation at UAG (amber) stop codons.^22−25^ As we show here, these GCE methods can be used to generate monomeric 14-3-3ζ homogeneously modified with pSer at position 58, yet the lability of pSer still hindered our study of 14-3-3ζ pSer58 in cellular-like environments. In-principle, this limitation can be overcome by substituting phospho-sites with nonhydrolyzable (i.e., stable), functional analogs or mimics (Figure 1). The negatively charged amino acids aspartate and glutamate (Figure 1A) are trivial to incorporate into proteins, but their charge, shape and hydrogen-bonding geometries are all markedly different than those of pSer and, in the case of 14-3-3 studies, they are insufficient to promote 14-3-3/client interactions^14,26,27^ and also fail to fully monomerize 14-3-3.^17,20,28,29^

A better structural mimic than Asp and Glu is phosphono-methyl-alanine (also called 2-amino-4-phosphonobutyrate), in which the bridging γ-oxygen of pSer is replaced with a CH_2_ (Figure 1A). For simplicity, we referred to it here as “non-hydrolyzable pSer” (nhpSer). Some direct assessments of how nhpSer recapitulates the functional effects of authentic protein serine phosphorylation have been done using synthetic peptides^30,31^ and protein chemical ligation^32^ approaches, but examples are sparse, resulting in an uncertainty in the field regarding the utility of nhpSer as a general mimic due to (i) loss of the γ-oxygen H-bond acceptor of pSer and (ii) a higher p*K*_a2_ of ∼7–8 compared to ∼5–6 for pSer. nhpSer has been translationally incorporated into proteins at genetically programmed amber (TAG) stop codons using GCE in both *E. coli*(33) and mammalian cells,^34^ but as we show in this work, the yields of nhpSer-protein expression were too low for the *E. coli* system to access biologically relevant, stably phosphorylated proteins.

Thus, for advancing our studies of both monomerized 14-3-3 and 14-3-3/client complexes, we here have (1) generated an efficient and scalable nhpSer *E. coli* GCE system suitable for expressing biologically relevant proteins, (2) verified the mimicry of nhpSer for pSer in three distinct physiologically relevant test cases, and (3) advanced our understanding of the activities of 14-3-3 proteins by generating a comprehensive data set for how the interactome of 14-3-3ζ changes when it is monomerized by phosphorylation at Ser58. Foundational to accomplishing these goals, and now available for advancing the studies of the many biochemical processes involving pSer-containing proteins, is an engineered self-sufficient *E. coli* GCE expression system—which we have dubbed “PermaPhos”—that biosynthesizes nhpSer internally from the central metabolite phosphoenolpyruvate, enabling the generation of milligram quantities of biologically relevant proteins containing site-specifically incorporated nhpSer.

## Results

### Phosphatases in *E. coli* Can Hydrolyze pSer-Containing Proteins Produced by GCE

Production of homogeneously phosphorylated proteins is critical to our ability to study their structure and function. Though *E. coli* does not have elaborate phospho-signaling systems like eukaryotes, we and others have seen that GCE produced phospho-proteins can be dephosphorylated during protein expression,^23,35,36^ frustrating downstream *in vitro* characterization. For example, Sluchanko and colleagues were unable to produce homogeneously phosphorylated SARS-CoV-2 nucleocapsid protein containing pSer due to unwanted hydrolysis, but they succeeded in doing so with nhpSer.^23^ As an additional example here, when we tried expressing a segment of mitogen-activated protein kinase kinase kinase 7 (MAP3K7/TAK1) phosphorylated at Ser439 in *E. coli* using a high-fidelity pSer GCE system, more than half purified as hydrolyzed even in the presence of phosphatase inhibitors (Figure S1). In these scenarios the ability to install a nonhydrolyzable pSer mimic is a necessary alternative to installing pSer.

### Evaluation of the Existing nhpSer GCE System

Previously, nhpSer was installed into proteins at programmed amber (TAG) stop codons with GCE by adding nhpSer to the culture media while expressing the same amino-acyl tRNA synthetase (aaRS)/tRNA pair and EF-Tu (EF-Sep) that allowed for pSer incorporation.^33^ To ensure exogenously added nhpSer was preferentially incorporated over pSer, an *E*. coli Δ*serC* mutant was used while also overexpressing *serB* to hydrolyze any pSer that might get into the cell from the media or formed by promiscuous transaminases that substitute for SerC function (Figure 1B).^33^ Our first goal was to quantify the efficiency of that system by using it to incorporate either one or two nhpSer residues into the sfGFP reporter protein. Using conditions of 2 mM nhpSer in the media culture, the fluorescence values obtained were slightly above those of cultures lacking nhpSer for single TAG codon suppression (corresponding to ∼1–2 mg per liter culture), but the double TAG codon suppression cultures produced indistinguishable quantities whether nhpSer was added or not (Figure S2). Proteins from these cultures were purified, and their phosphorylation status was evaluated initially using Phos-tag gel electrophoresis in which phosphorylated proteins migrate more slowly than their nonphosphorylated proteins and serendipitously nhpSer proteins migrate more slowly than their pSer counterparts.^34,37^ Purified protein produced from the single TAG site suppression was consistent with protein containing nhpSer with a minor contamination of pSer-containing protein (Figure S2).

This confirms that the published GCE system is indeed functional for nhpSer incorporation, but it gives low yields and some contamination with pSer-containing protein that could be difficult to purify away. We reasoned this low efficiency was due to low bioavailability of the nhpSer amino acid given that charged amino acids do not readily traverse cell membranes. Indeed, GCE systems for pSer and pThr incorporation (as well as sulfo-tyrosine) overcame this issue of low target amino acid bioavailability by leveraging biosynthetic pathways that produce high intracellular amino acid concentrations,^22,33,35,38,39^ and so we sought to do the same for nhpSer.

### Developing an Efficient nhpSer-Protein Expression System

#### A Putative nhpSer Biosynthetic Pathway

**Figure 2:**
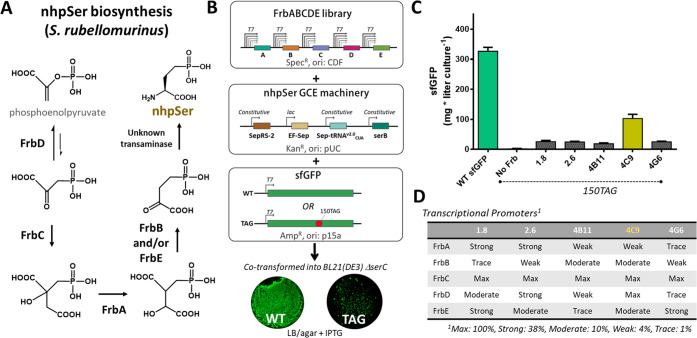
Construction of functional FrbABCDE biosynthetic pathways for translational incorporation into sfGFP in *E. coli*. (A) Biosynthetic pathway for nhpSer by *S. rubellomurinus*. The first committed step of nhpSer biosynthesis is catalyzed by a phosphoenolpyruvate mutase, FrbD, which forms the unusual C–P phosphonate bond. The energetically unfavorable equilibrium is pulled forward by the homocitrate synthase homologue FrbC via irreversible condensation of acetyl-CoA. The next two steps parallel those of the TCA cycle and are carried out by FrbA (an aconitase) and Frbs B and/or E (divergent homologues of isocitrate dehydrogenase). It is not yet known whether FrbB or FrbE catalyze this reaction, or if both are required.^40−42^ It may be possible the FrbB and FrbE form a heterodimer, or that the two-step isocitrate dehydrogenase reaction is decoupled such that one catalyzes NADP-dependent oxidation while the other performs decarboxylation. (B) A library of FrbABCDE pathways containing all combinations of Frb enzyme transcriptional strengths was cotransformed with a plasmid housing all nhpSer GCE components and either a sfGFP-WT or sfGFP-150TAG reporter plasmid into BL21(DE3) Δ*serC* cells. (C) Comparison of nhpSer incorporation efficiency into sfGFP-150TAG for the top 5 isolated FrbABCDE pathways compared to wild-type sfGFP expression. Error bars represent standard deviations of expressions performed in triplicate. (D) Promoter identities for the Frb enzymes for each isolated pathway shown in panel C. Promoter sequences are indicated in Figure S3.

A putative biosynthetic pathway for nhpSer was discovered in 2010, when Zhao and colleagues probed the 10-step pathway by which *Streptomyces rubellomurinus* converts phosphoenolpyruvate into the antimalarial natural product FR-900098.^40,41^ After reconstituting the full 10-step pathway in *E. coli*, they showed that nhpSer plus the last four enzymes in the pathway led to FR-900098 production.^40,41^ This implied that nhpSer was a pathway intermediate produced by enzymes FrbA, FrbB, FrbC, FrbD, and possibly FrbE, plus a transaminase that is not part of the Frb biosynthetic cluster, which can be substituted for by an *E. coli* transaminase^40−42^ (Figure 2A).

#### Engineering *E. coli* to Make nhpSer for Incorporation into Proteins

Reconstitution of biosynthetic gene clusters in heterologous organisms benefits from tuning enzyme activities so as to maximize product while not exhausting cellular resources or creating toxic side effects.^43^ To do this for nhpSer biosynthesis, we adopted the strategy used to optimize the yields of FR-900098.^42^ First, a randomized library of T7 promoter variants was screened in the BL21(DE3) Δ*serC* cell line to identify five variants that drove sfGFP expression at discrete levels spanning 2 orders of magnitude (∼100, 40, 10, 4, and 1% that of wild-type) (Figure S3). Next, a combinatorial *FrbABCDE* library was created containing all permutations of the five Frb genes under the control of the five promoter variants (5^5^ = 3125 pathway assemblies, Figure 2B).

To select for functional Frb pathway assemblies, we cotransformed the *FrbABCDE* library into BL21(DE3) Δ*serC* cells along with a single plasmid housing all nhpSer GCE machinery components (including a constitutively overexpressed *serB* gene) and a third plasmid expressing sfGFP-150TAG (Figure 2B). Using sfGFP fluorescence as a readout of nhpSer biosynthesis and subsequent incorporation, the top 5 performing Frb assemblies (Figure 2C) were isolated, representing 5 unique combinations of promoters (Figure 2D). FrbC was transcribed at maximal levels in all selected clones, likely to more effectively “pull” the unfavorable equilibrium catalyzed by FrbD forward. As one of the expression vectors (4C9) strongly outperformed the others (Figure 2C), we chose it for further characterization and named it “Frb-v1”.

**Figure 3:**
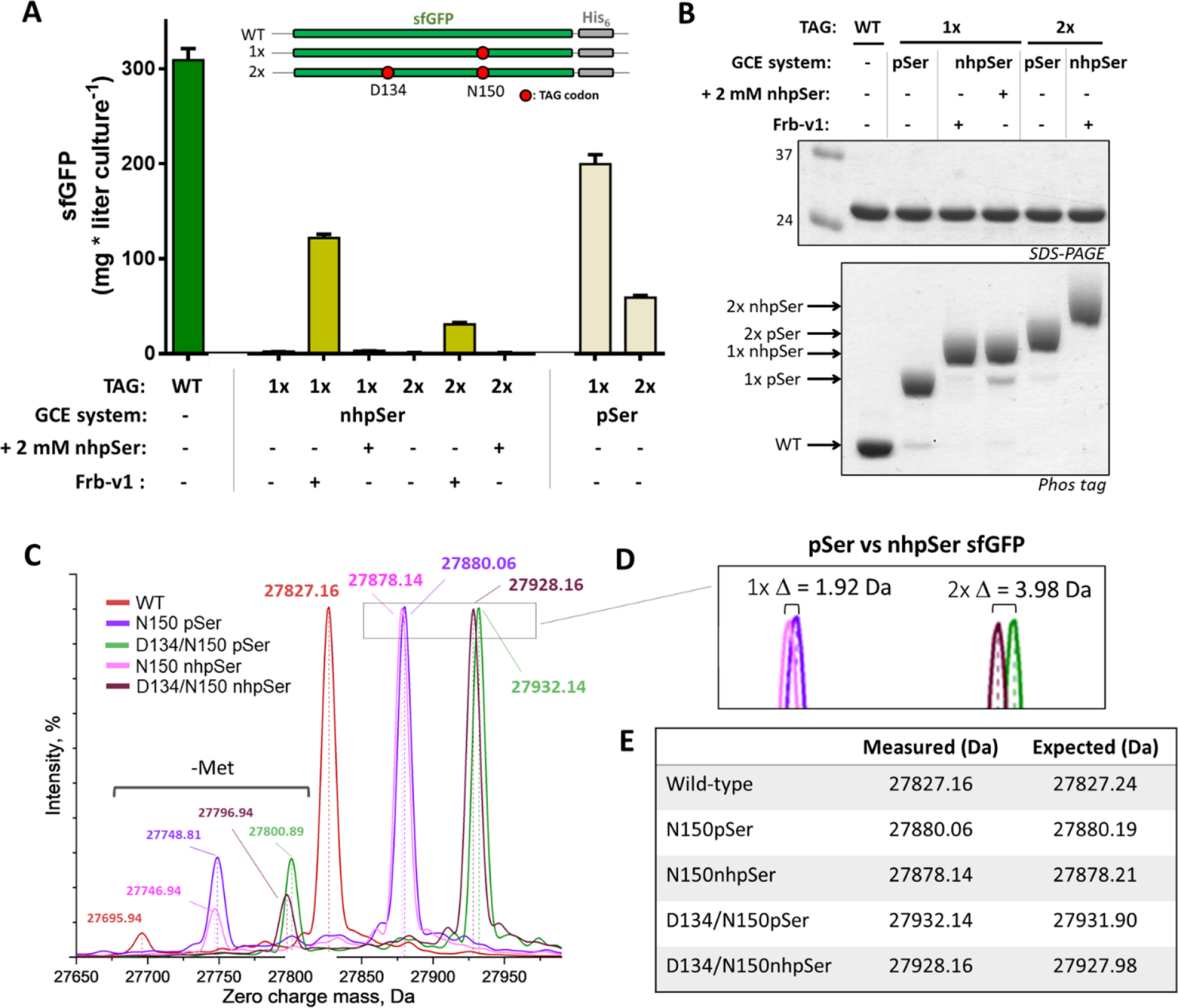
Evaluation of the Frb-v1 pathway efficiency and fidelity. (A) Comparison of single (1×, 150TAG) and double site (2×, 134/150TAG) nhpSer incorporation in sfGFP when nhpSer is biosynthesized via the Frb-v1 pathway and when supplemented in the media at 2 mM concentration. The wild-type expression level and the efficiency of pSer GCE systems are shown for comparison. Error bars represent standard deviations of expressions performed in triplicate. (B) SDS-PAGE and Phos-tag gels of the purified proteins expressed in panel A. Phos-tag gels contain an acrylamide derivative that transiently interacts with phospho-groups, causing phospho-proteins to migrate slower than unmodified proteins.^37^ NhpSer-containing proteins migrate slower than their equivalent pSer-containing proteins, providing a convenient method to distinguish between the two isosteres.^34,69^ For each lane 2 μg of protein was loaded, so band intensities are not reflective of expression yield. (C) Whole-protein mass spectrometry of single and double site pSer and nhpSer incorporation into sfGFP (when nhpSer is biosynthesized by the Frb-v1 pathway). (D) Zoom in of the peaks in panel C for 1× and 2× pSer/nhpSer incorporated sfGFP confirm the expected mass differences of a bridging O to CH_2_ substitution. (E) Measured and expected whole-protein masses shown in panels C and D.

Using the Frb-v1 reconstituted nhpSer pathway, single and double nhpSer incorporations into sfGFP yielded ∼120 and 30 mg/L culture, respectively (Figure 3A). These values are dramatically (∼40 fold) higher than when cells without Frb-v1 are supplemented with nhpSer at 2 mM concentration, are more than 50% of the expression levels achieved with optimized pSer GCE expression conditions, and correspond to about 40% and 13%, respectively, of wild-type sfGFP expression (Figure 3A).

#### Fidelity Assessment of nhpSer Incorporation

Proteins from all the above expressions were purified by standard metal-affinity chromatography to validate nhpSer incorporation. All purified sfGFP forms migrated the same on SDS-PAGE gels (Figure 3B, top), but on Phos-tag gels, the sfGFP-150nhpSer proteins produced via Frb-v1 biosynthesis and nhpSer supplementation to the media migrated identically and more slowly than sfGFP-150pSer (Figure 3B, bottom), consistent with their reported properties.^34^ Similarly, the sfGFP-134/150nhpSer protein produced via Frb-v1 biosynthesis migrated more slowly than the equivalent protein doubly substituted with pSer and gave no clear evidence of contaminating pSer-containing protein. Electrophoretic comparison with sfGFP-134/150nhpSer made via nhpSer supplementation was not feasible due to insufficient expression levels.

We next used ultra-high-resolution mass spectrometry to directly confirm the translational incorporation of nhpSer into sfGFP. All proteins—wild-type, pSer, and nhpSer sfGFP (single and double site incorporation)—had average molecular masses within 0.25 Da (<10 ppm) of their expected values (Figure 3C–E). Evaluating the pSer-containing sfGFP side-by-side with the nhpSer-equivalent proteins confirmed that our whole-protein MS methods were sufficiently accurate to observe the small mass differences between pSer and nhpSer (expected Δ = 1.98 and 3.96 Da for the O to CH_2_ substitution at the bridging γ-atom for single and double site incorporation, respectively, Figure 3D). Further, electron transfer dissociation MS/MS fragmentation of tryptic digests unambiguously confirmed the location of nhpSer and pSer at the targeted sites D134 and N150 in the doubly modified sfGFP variants (Figures S4 and S5). We also did not find, within limits of detection, pSer or any natural amino acid misincorporated at the D134 and N150 sites of nhpSer incorporation when we expanded the search space to include all possible variations (data not shown).

#### nhpSer in Proteins Made with Frb-v1 Are Resistant to Hydrolysis

To confirm nhpSer-containing proteins made via the Frb-v1 pathway are indeed resistant to phosphatase activity, we incorporated pSer and nhpSer at the functionally relevant site of Ser16 of the small heat shock protein B6 (HSPB6)^44^ expressed as a peptide (residues 11–20) fusion with a highly soluble SUMO protein (Figure S6A). We verified nhpSer and pSer incorporation by Phos-tag gels, and upon incubation with λ-phosphatase, the pSer-containing protein was hydrolyzed while the nhpSer-containing protein was not (Figure S6B). These data confirm that reconstitution of the FrbABCDE pathway in *E. coli* cells in combination with the nhpSer GCE machinery creates a fully autonomous organism with a 21-amino acid genetic code able to efficiently synthesize protein with permanent phosphoserine mimics site-specifically installed, i.e. “PermaPhos”.

### Assessing nhpSer Mimicry of pSer Function in Multiple Protein Contexts

The above data show that PermaPhos provides a simple and efficient strategy for site-specific installation of nhpSer into proteins and thus overcomes a key barrier to its use as a pSer mimetic for proteins expressed in *E. coli*. Given general reservations that nhpSer may not mimic pSer effectively,^45^ we used PermaPhos to assess the extent to which nhpSer-containing proteins recapitulate the function of pSer-containing proteins, particularly in cases where Asp or Glu do not. First, we directly measured the p*K*_a2_ values of nhpSer and pSer on a protein to compare their charge state at physiologic pH. Then, for each of three major regulatory outcomes of serine phosphorylation—(i) activation of enzymatic activity, (ii) stabilization of protein–protein interactions, and (iii) disruption of protein–protein interactions—we selected one biologically relevant system for which we could produce the equivalent homogeneous pSer protein forms via GCE and for which Asp/Glu mutations are not effective mimetics, and then we directly compared the impact of nhpSer vs pSer vs Asp/Glu. This choice to use proteins that could be homogeneously produced with both pSer and nhpSer was critical to effectively comparing their structure, function and/or thermodynamic properties.

#### Charge State of nhpSer at Physiological pH

**Figure 4:**
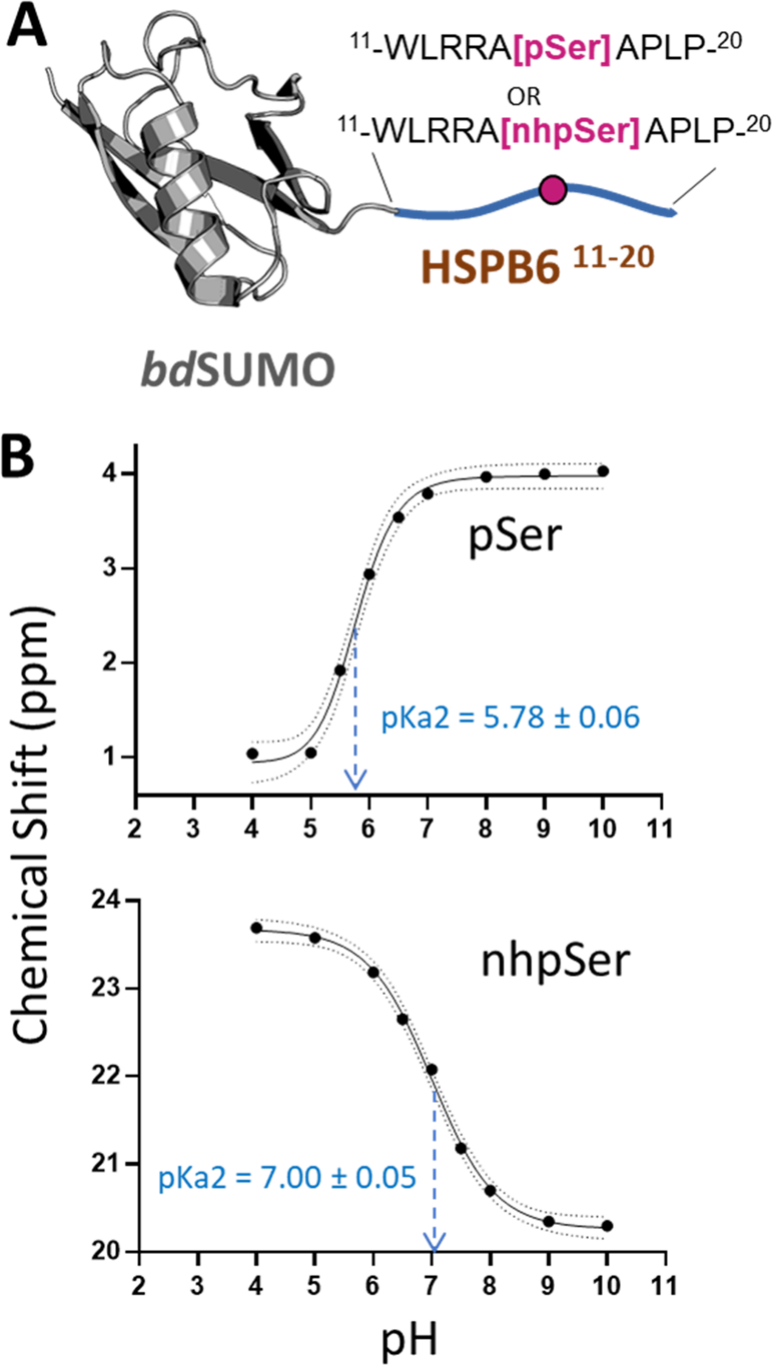
p*K*_a2_ measurement of pSer and nhpSer in the context of a protein. (A) pSer and nhpSer were incorporated into site Ser16 of HSPB6 (residues 11–20) fused to a *bd*SUMO domain. Phos tag gels confirming pSer and nhpSer incorporation are shown in Figure S6. (B) Plotting of ^31^P NMR chemical shifts revealed a sigmoidal curve from which the p*K*_a2_ of each phospho-moiety was extrapolated at the inflection point. Dotted lines represent the 95% confidence interval of the fitted curves. Raw ^31^P NMR spectra are shown in Figure S7.

Methylene phosphonates like nhpSer have reported p*K*_a2_ values ranging from 7 to 8, compared to ∼5–6 for phosphates.^46^ Whether this p*K*_a2_ is closer to 7 or to 8 changes its protonation state at physiologic state quite notably, yet we found no studies that have measured this directly in the context of a protein. To do this, we used the above-described SUMO-HSPB6 fusion protein with pSer and nhpSer at Ser16 (Figure 4A) and followed the ^31^P NMR chemical shifts as a function of pH.^47,48^ This construct was chosen because it was highly soluble, stable, and small (i.e., suitable for NMR) and also because the phospho-site is within an unstructured segment such that phosphorus resonance changes should reflect the protonation state rather than changes in the protein conformation. We observed a well-defined ^31^P peak for nhpSer- and pSer-containing proteins, and consistent with previous work, increasing pH resulted in an upfield ^31^P chemical shift for nhpSer and a downfield shift for pSer,^47^ with the inflection points indicating a p*K*_a2_ of 7.00 ± 0.05 for nhpSer and 5.78 ± 0.06 for pSer (Figures 4B and S7). These data imply that at pH 7.4, nhpSer in this context exhibits an average charge of −1.7 (∼71% dianonic), compared to −2.0 for pSer, whereas that for the carboxylate of Asp/Glu is −1.0.

#### Mimicking pSer-Dependent Kinase Activity

**Figure 5:**
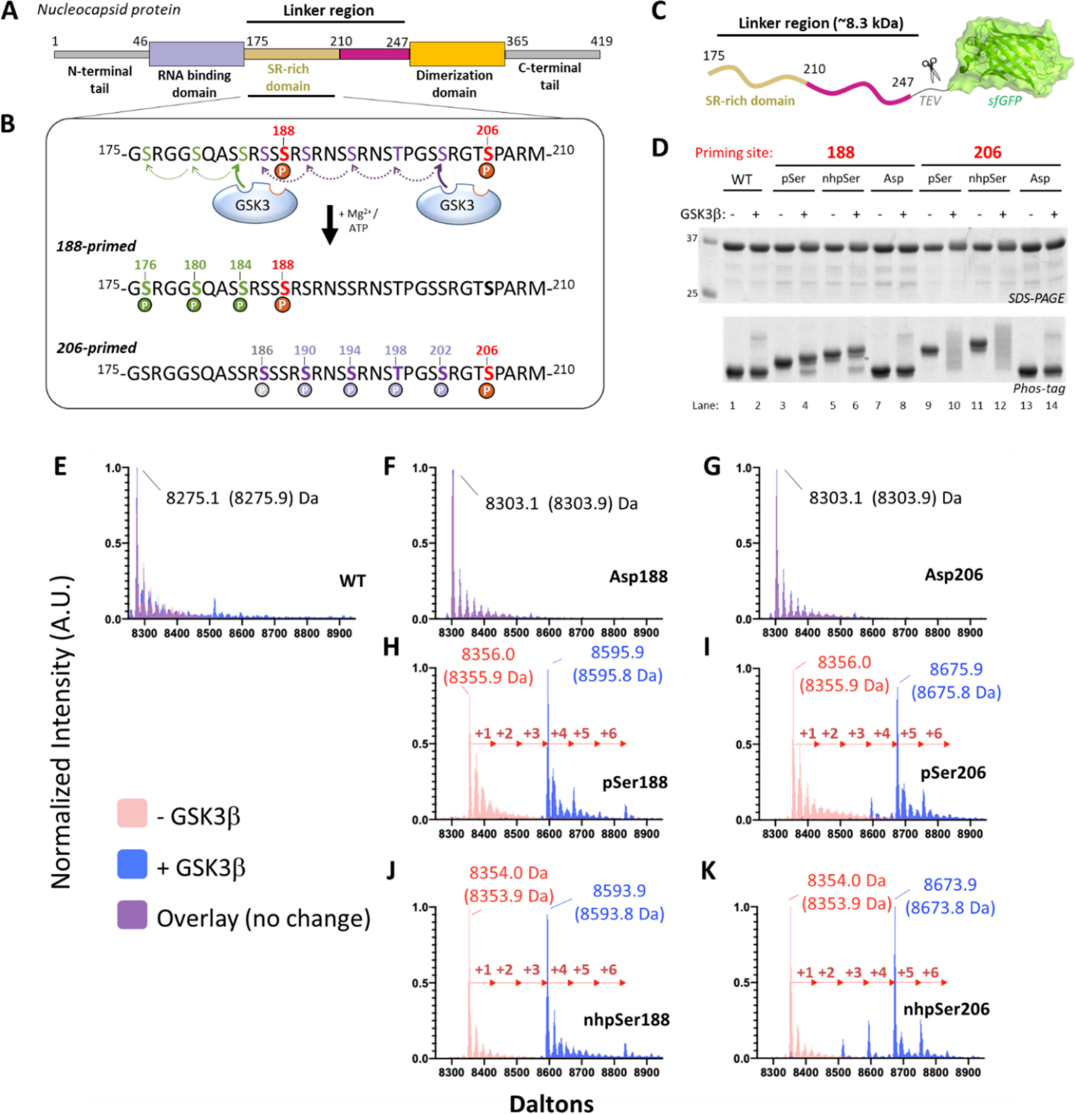
Using nhpSer as a GSK3β activator. (A) The SARS-CoV-2 nucleocapsid phosphoprotein (Np) contains a serine-arginine rich region (SR) in its linker region (Linker Np, residues 175–247) that connects the N-terminal RNA binding domain and the C-terminal dimerization domain. (B) Proposed mechanism of GSK3β poly-phosphorylation of the SR region of Np: sites S188 and S206 act as priming sites of GSK3β activity when phosphorylated, resulting in two distinct tracts containing up to 3 and 5 additional sites of phosphorylation, respectively. (C) The Linker-Np was genetically fused to a TEV cleavable C-terminal sfGFP protein for enhanced solubility. (D) Ser, Asp, pSer and nhpSer were incorporated at sites S188 and S206 within the Linker-Np, purified, and mixed with ATP, Mg^2+^, and GSK3β. SDS-PAGE and Phos-tag gels of reaction products confirm phosphorylation of Linker-Np by GSK3β only in the pSer and nhpSer primed Linker-Np proteins, and not the Ser or Asp proteins, as evidenced by the gel-shifts observed in the Phos-tag gel. (E–K) Whole-protein mass spectrometry of Linker-Np (without sfGFP) before (pink) and after (blue) GSK3β reactions. Overlapping spectra where no change occurs combine to be purple in color. (E) WT, (F) S188Asp, (G) S206Asp, (H) S188pSer, (I) 206pSer, (J) 188nhpSer, (K) 206nhpSer. Arrows indicate +80 Da increments corresponding to the addition of a phosphate group. Theoretical masses of the dominant protein species are shown in parentheses.

Glycogen synthase kinase-3β (GSK3β) is a serine/threonine kinase that regulates numerous signaling pathways. Rather than being activated by direct phosphorylation, GSK3β activity is activated or “primed” by a Ser/Thr phosphorylated substrate, which is pre-phosphorylated by another kinase; GSK3β then phosphorylates Ser/Thr residues four positions N-terminal to the substrate-priming phospho-site (Figure 5A and 5B).^49^ The binding of primed substrates activates GSK3β by inducing conformational changes similar to those triggered in canonical kinases when they are phosphorylated in their activation loop^50^ (Figure S8). A hallmark feature of GSK3β is the so-called “zippering” effect in which each residue phosphorylated by GSK3β can serve as the next priming site for another Ser/Thr N-terminal to it, resulting in substrate poly-phosphorylation.^51^ Asp/Glu mimetics are not considered sufficient to prime GSK3β activity.^52,53^

As a stringent test of pSer mimicry, we asked whether nhpSer could serve as a priming site to activate GSK3β. Due to its relevance to the current Covid-19 pandemic, we focused on the GSK3β hyperphosphorylation of the “Linker” region of the SARS-CoV-2 nucleocapsid protein (Np) (Figure 5A). This modification is essential to the viral life cycle,^54,55^ apparently facilitating the release of genomic RNA from Np for viral protein translation^56−58^ and also complexation with 14-3-3.^59^ Sites S188 and S206 of Np are priming sites for GSK3β phosphorylation, resulting in the potential addition of 3 and 5 phosphorylation events, respectively (Figure 5B). We purified the relevant linker region of Np as a fusion of sfGFP (Figure 5C) with Ser (wild-type), pSer, nhpSer, and Asp at sites 188 or 206, and we incubated them with GSK3β, ATP, and Mg^2+^. Phos-tag gel analysis showed that only trace amounts of wild-type, S188D, and S206D variants were phosphorylated (Figure 5D, lanes 1/2, 7/8, and 13/14), whereas Linker Np-pSer188 and -pSer206 both underwent substantial electrophoretic band shifts, indicative of phosphorylation by GSK3β (Figure 5D, lanes 3/4 and 9/10). Analogous band shifts were observed for the Linker Np-nhpSer188 and Np-nhpSer206 variants upon incubation with GSK3β, though slightly upshifted as expected for nhpSer-containing proteins (Figure 5D, lanes 5/6 and 11/12). We also conducted the same GSK3β reactions using full-length Np and observed the same pattern of Phos-tag electrophoretic mobility shifts when Ser, Asp, pSer and nhpSer were placed at sites 188 or 206 (Figure S9).

We then used mass spectrometry to compare in greater detail the identity of the products resulting from pSer-primed vs nhpSer-primed GSK3β phosphorylation of the Np Linker (after removing the sfGFP). This analysis showed the production of only a trace amount of phosphorylated product for wild-type, S188D, and S206D (Figures 5E–G, S10, S11, and S16), consistent with the Phos-tag gel results (Figure 5D). GSK3β incubation with the pSer188- and pSer206-primed Np Linkers produced a dominant species with 3 and 4 added phospho-sites, respectively (Figure 5H and 5I). MS/MS of the dominant poly-phosphorylated species confirmed the locations of the added phosphates were exactly as expected for the GSK3β “zippering” effect (Figures S12, S13, S17, and S18). Finally, analyses of GSK3β reacted nhpSer188 and nhpSer206 Linker-Np produced the same dominant species as the pSer primed proteins (Figure 5J and 5K), with identical locations of the added phospho-sites (Figures S14, S15, S19, and S20). We conclude from these experiments that nhpSer faithfully recapitulates pSer-dependent activation of GSK3β.

#### Mimicking pSer-Dependent Stabilization of Protein–Protein Interactions

**Figure 6:**
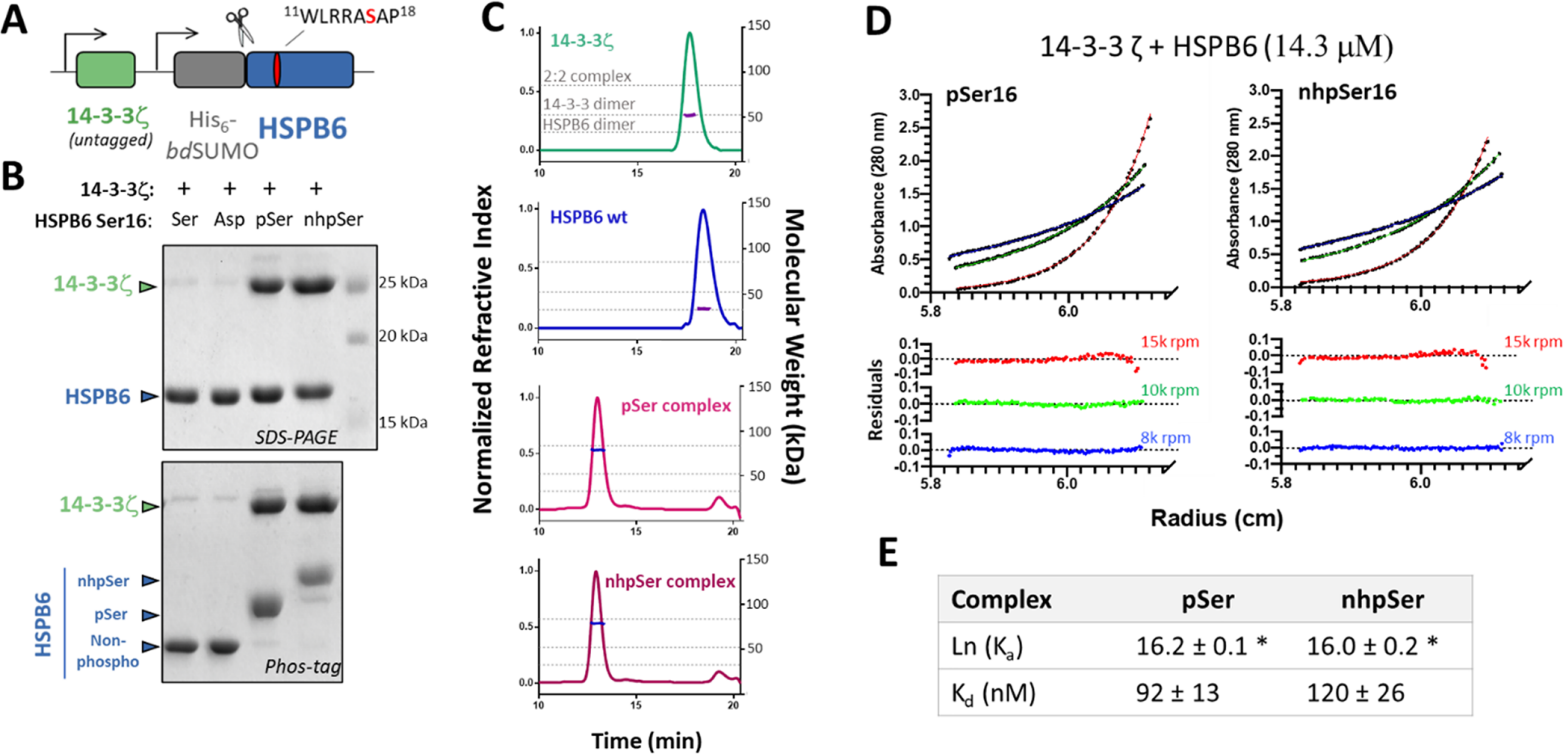
Mimicking pSer-dependent stabilization of 14-3-3/HSPB6 interactions with nhpSer. (A) Untagged 14-3-3ζ and His_6_-*bd*SUMO-HSPB6 proteins were coexpressed such that if they formed a tight complex, 14-3-3ζ copurifies with HSPB6. At site 16 of HSPB6, which lies within a conical and well-established 14-3-3 binding site, either serine, aspartate, pSer or nhpSer were incorporated. (B) SDS-PAGE gels (top panel) of the purified proteins show that 14-3-3ζ copurifies with HSPB6 when pSer and nhpSer, but not Ser or Asp, were incorporated at site S16. Phos-tag gel electrophoresis (bottom panel) of these proteins confirmed nhpSer incorporation into HSPB6 as indicated by it having the slowest electrophoretic mobility, with pSer being intermediate between nhpSer and wild-type or S16D HSPB6. (C) SEC-MALS of the purified protein complexes confirms 2:2 stoichiometry. Dotted gray lines denote the theoretical molecular weights of the 2:2 complex (85.4 kDa), the 14-3-3ζ dimer (52.4 kDa) and the HSPB6 dimer (33.0 kDa). Data statistics are shown in Table S1. (D) Equilibrium AUC of the pSer (left) and nhpSer (right) HSPB6/14-3-3ζ complexes at 14.3 μM concentration at 15k (red), 10k (green) and 8k (blue) rpm. Residuals from fitting the equilibrium data to a two-state A + B ↔ AB model are shown in the bottom panels. Equilibrium data for 7.2 μM and 3.6 μM concentrations are shown in Figure S23. (E) Global fitting of 14-3-3ζ/HSPB6-pSer16 and 14-3-3/HSPB6-nhpSer16 equilibria at three different protein concentrations and at three different centrifugation speeds revealed dissociation constants of 92 ± 13 and 120 ± 26 nM, respectively. Error represents 95% confidence interval range. * Denotes statistically indistinguishable values as calculated by student’s unpaired *t* test, *p* = 0.05; *t* = 0.832.

Next, we tested the ability of nhpSer to promote pSer-dependent protein–protein interactions. For this we chose the 14-3-3 family of dimeric hub proteins that bind to and regulate hundreds of “client” pSer/pThr-containing proteins^7^ and for which it is well documented that Asp/Glu are not functional mimics.^14,26,27^ While a short nhpSer peptide has been shown to bind 14-3-3 with comparable affinity as the corresponding pSer peptide,^30^ neither affinities nor the stoichiometries of complex formation have been measured in the context of the physiologically relevant full-length client and 14-3-3 proteins. For a model client of 14-3-3, we chose full-length HSPB6 because it forms a structurally and thermodynamically well-characterized high-affinity complex with 14-3-3 when phosphorylated at Ser16.^44,60^ For initial assessment of 14-3-3/client complexation, we adopted our previously reported *E. coli* expression system^22^ in which untagged 14-3-3 (ζ-isoform) is coexpressed with His-tagged full-length HSPB6, so that 14-3-3ζ would copurify with HSPB6 only if they formed a stable complex (Figure 6A).

Expressing full-length HSPB6 with either Ser, pSer, nhpSer, or Asp at position 16, 14-3-3ζ copurified with HSPB6-pSer16 and with HSPB6-nhpSer16, but not HSPB6-WT or HSPB6-S16D (Figures 6B and S21), confirming that only pSer and nhpSer promote a stable complex between HSPB6 and 14-3-3ζ. We further confirmed by size-exclusion chromatography coupled to multiangle light scattering (SEC-MALS) that the complexes of 14-3-3 with HSPB6-pSer16 and with HSPB6-nhpSer16 both have 2:2 stoichiometry (Figure 6C and Table S1) as expected.^44^ In contrast, when either purified HSPB6-WT or the S16D variant were mixed with 14-3-3ζ, they remained uncomplexed by SEC-MALS analysis (Figure S22).

We next used equilibrium analytical ultracentrifugation (AUC) to directly measure the affinities of the pSer and nhpSer promoted HSPB6/14-3-3 complexes. Global fitting of the 14-3-3/HSPB6-pSer16 and -nhpSer16 2:2 complexes fit well to a two-state model and yielded statistically indistinguishable dissociation constants of 92 ± 13 and 120 ± 26 nM, respectively (Figures 6D, 6E, S23 and S24). These measured *K*_d_ values are smaller than but consistent with the previously reported *K*_d_ of 560 ± 200 nM for the pSer-dependent complex extrapolated from fluorescently labeled HSPB6.^44^ Regardless, HSPB6 with nhpSer or pSer at site S16 binds 14-3-3 with indistinguishable affinity, while the S16D variant forms no detectable complex with 14-3-3. These data confirm that the nhpSer-stabilized HSPB6/14-3-3ζ complex recapitulates the authentic complex stabilized by pSer.

#### Mimicking pSer-Dependent Disruption of Protein–Protein Complexes

**Figure 7:**
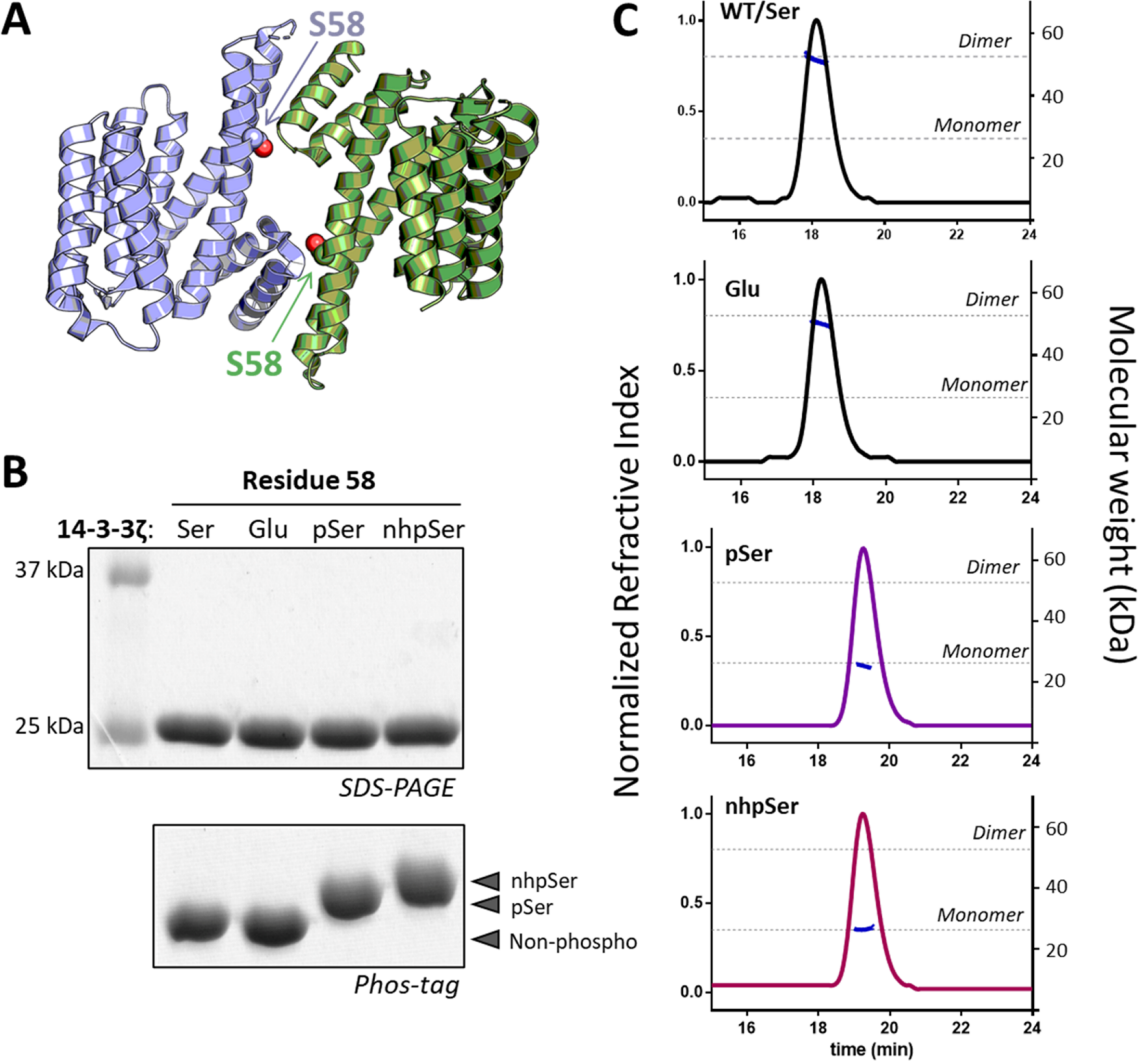
Mimicking pSer-dependent disruption of the 14-3-3 dimer. (A) Ser58 lies at the dimer interface of 14-3-3ζ (PDB 6F08). Individual protomers are colored in blue and green. (B) SDS-PAGE and Phos-tag gels confirm pSer and nhpSer incorporation into 14-3-3ζ. (C) SEC-MALS of the four 14-3-3ζ protein variants shown in panel B confirm the dimeric status of WT and S58E 14-3-3ζ, while pSer58 and nhpSer58 variants elute exclusively as a monomer. Dotted lines indicate theoretical masses of the dimeric (52.4 kDa) and monomeric 14-3-3 (26.2 kDa). All proteins were injected onto the SEC column at 50 μM initial concentration. Data statistics are shown in Table S2.

The dimeric status of 14-3-3 isoforms ζ, ε, γ, η and β is regulated by phosphorylation of a serine—Ser58 in 14-3-3ζ—at the dimer interface, leading to its monomerization (Figure 7A).^10,61^ Phosphorylated, monomeric 14-3-3 is thought to sensitize cells to apoptosis, sparking interest in the development of therapeutics that stabilize it.^13^ But studying this form of 14-3-3 has been challenging due to an inability to make it: Asp/Glu phosphomimetic mutations are reported to weaken the 14-3-3 dimer, albeit poorly compared to phosphorylation,^17,20,28,29^ and kinase phosphorylation results in incomplete modification due to the hidden interfacial location of this residue.^14−16^ Characterizations of monomeric 14-3-3 have thus historically relied on deleting the entire N-terminal dimerization interface,^21^ adding interface mutations or, very recently, a multistep strategy to purify the phosphorylated away from the unmodified form.^20,29^ Using pSer and PermaPhos GCE, we expressed and then purified homogeneous 14-3-3ζ with Ser, pSer, nhpSer, as well as Glu at position S58 (Figure 7B), and we evaluated their oligomeric status using SEC-MALS (Figure 7C and Table S2). The 14-3-3ζ WT eluted as a single dimeric peak at 50 μM initial concentration (∼2-fold above physiologic concentration^20^), as did the phosphomimetic S58E variant, consistent with previous work.^20,28^ In contrast, both 14-3-3ζ pSer58 and 14-3-3ζ nhpSer58 eluted exclusively as a monomer. In addition, we confirmed that 14-3-3ζ nhpSer58 retained an intact phospho-peptide binding groove just like 14-3-3ζ pSer58 (Figure S25). Thus, nhpSer at position 58 recapitulates the phosphorylation-dependent monomerization of 14-3-3, while Glu at this position does not.

#### Functional Consequences of 14-3-3 Monomerization by Phosphorylation

##### Revealing the Interactome of Monomeric 14-3-3ζ

With 14-3-3ζ nhpSer58 in hand as a nonhydrolyzable phosphorylated mimic that monomerizes 14-3-3ζ, we set out to determine how this monomerization alters 14-3-3ζ function and rewires cellular signaling systems. As noted earlier, prior attempts to shed light on this have largely relied on “dimerization deficient” 14-3-3 variants that had no phosphoryl group at position 58, and instead had multiple dimer interface mutations, leaving it unclear to what extent the results accurately captured the behavior of phosphorylated, monomeric 14-3-3.^21^

To more effectively address this question, 14-3-3ζ with Ser, pSer, and nhpSer at position 58 were each covalently immobilized onto Sepharose beads and incubated with HEK293T lysates for sufficient time to form complexes with endogenous proteins. Then after extensive washing of the resins, we used denaturant to elute interacting partner proteins. Since we could not directly assess the phosphorylation status of the Sepharose-linked 14-3-3ζ pSer58 and nhpSer58 proteins after lysate incubation, we separately incubated free FLAG-tagged versions of these proteins in lysates for an identical time period, and we found by Western blotting of Phos-tag gels that the 14-3-3ζ pSer58 protein had been completely dephosphorylated, while the 14-3-3ζ nhpSer58 was unchanged (Figure S26). Being immobilized on the surface of Sepharose beads rather than free in solution could offer 14-3-3ζ pSer58 an added level of protection from phosphatases, and so we still analyzed the interactomes isolated from all three pull-downs with the expectation that the observed 14-3-3ζ pSer58 interactome would be a challenging-to-interpret mix of proteins binding both monomeric and dimeric 14-3-3ζ.

**Figure 8:**
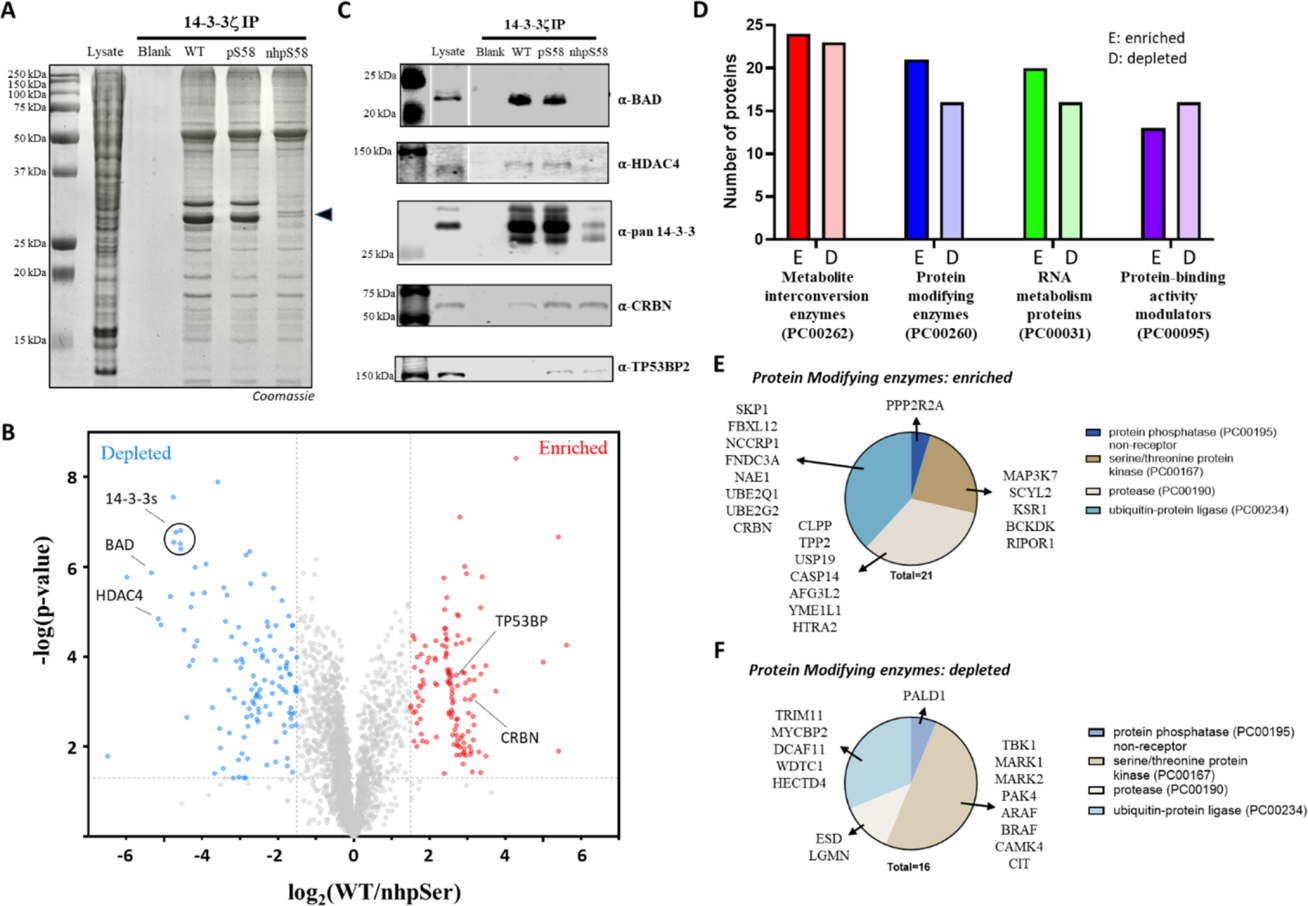
14-3-3ζ interactome rearrangement in response to monomerization by phosphorylation at Ser58. (A) Coomassie-stained SDS-PAGE gel of proteins in HEK293T lysate eluted from 14 to 3-3ζ (WT, pSer58 and nhpSer58)-conjugated sepharose resin. Lysate: soluble lysate prior to incubation with 14-3-3ζ-sepharose. Blank: proteins eluted from sepharose lacking 14-3-3ζ. The black triangle indicates endogenous 14-3-3 proteins at ∼30 kDa. (B) Volcano plot comparing the pool of proteins identified in the 14-3-3ζ WT and nhpSer58 pull-downs shown in panel A as determined by label-free quantitative mass spectrometry. Proteins identified in red as “enriched” were at least 2.8-fold (log_2_ 1.5) more abundant in the 14-3-3ζ nhpSer58 pool compared to that of 14-3-3ζ WT (p-value < 0.05). Similarly, proteins identified in blue as “depleted” were at least 2.8-fold (log_2_ 1.5) less abundant in the 14-3-3ζ nhpSer58 pool compared to that of 14-3-3ζ WT (p-value < 0.05). Protein IDs, enrichment values and associated p-values from both biological replicates can be found in Supporting Data Set 1, with volcano plots shown in Figure S27. Similar data comparing 14-3-3 WT/pSer58 pull-downs can be found in Supporting Data Set 2, with volcano plots comparing them also shown in Figure S27. (C) Western blots quantifying abundances of proteins from the eluted pools shown in panel A confirmed depletion of BAD, HDAC-4, and endogenous 14-3-3s and enrichment of CRBN and TP53BP2 in the 14-3-3ζ nhpSer58 pool relative to that of the 14-3-3ζ WT. (D) PANTHER analysis quantifying the most enriched and depleted protein classes identified in the 14-3-3ζ pull-down data sets. (E and F) Among the proteins identified in the “protein modifying enzyme” class whose bindings to 14-3-3 were enriched (E) or depleted (F) by monomerization (blue bars, panel D) were a variety of kinases, ubiquitin-ligase proteins, proteases and phosphatases, suggesting the alteration of key signaling systems when 14-3-3ζ is phosphorylated at Ser58.

SDS-PAGE analysis of each of the three eluates revealed the presence of many proteins (Figure 8A), and because no proteins were detected when blank Sepharose beads were used, we infer that the isolated proteins had been interacting with the immobilized forms of 14-3-3ζ—either directly or indirectly as part of multiprotein complexes. One prominent feature of the SDS gels was that both the WT and pSer58 pull-downs included major protein bands near 30 kDa, which did not appear in the nhpSer pull-downs, consistent with the hydrolysis of the pSer58 complicating the results by leading to the presence of wild-type protein. Using label-free quantitative mass spectrometry, we identified and quantified several thousand proteins in each of the pools that had been pulled-down by 14-3-3ζ WT, pSer58 and npSer58 variants (Figures 8B and S27 and Supporting Data Set 1). We used volcano plots to compare the interactomes of the WT and nhpSer58 variants, and most of the identified proteins (∼85%) associated with both forms to a similar extent. Using cutoff criteria of a ≥2.8-fold increase or decrease in peptide abundance between data sets, and a p-value <0.05, 223 proteins were significantly enriched and 176 were significantly depleted in the monomeric 14-3-3ζ nhpSer58 interactome relative to WT.

Satisfyingly, among the most depleted proteins were endogenous 14-3-3 proteins (by ∼20-fold, p-value <10^–6^) which are expected to heterodimerize with the immobilized 14-3-3ζ WT but not the nhpSer58 variant. This difference could account for the prevalent SDS-PAGE bands at ∼30 kDa that are present in the 14-3-3ζ WT but not the nhpSer58 interactome (Figure 8A), and as a confirmation of that, Western blotting of these pools with a pan-14-3-3 antibody revealed these bands to be endogenous 14-3-3 proteins (Figure 8C). That 14-3-3ζ pSer58 pulled-down quite similar quantities of endogenous 14-3-3 proteins as WT (Figures 8A and C and S27 and Supporting Data Set 1) confirms that enough of the immobilized 14-3-3ζ pSer58 protein was hydrolyzed over the course of lysate incubation to contaminate those results with proteins that bind to dimeric 14-3-3. A lower amount of intact pSer58 protein remaining on the column could also explain why ∼20% fewer proteins were enriched in pull-downs with 14-3-3ζ pSer58 compared to nhpSer58, and the overall enrichment scores of these interactors were lower (e.g., only 27 proteins were enriched >8-fold in the pSer58 data set compared to 55 with the nhpSer58 data set).

To validate some additional proteins that showed a significant preferential interaction with either WT or nhpSer 14-3-3ζ, we performed Western blots probing for two proteins that had been enriched and two that had been depleted. We confirmed that Bcl2-associated agonist of cell death (BAD) and histone deacetylase 4 (HDAC4) were depleted in the 14-3-3ζ npSer58 pull-downs compared to wild-type 14-3-3ζ, while cereblon (CRBN) and the tumor protein p53 binding protein 2 (TP53BP) were enriched (Figure 8C). Thus, the former two proteins (BAD, HDAC4) bind monomeric phosphorylated 14-3-3ζ less well than the WT dimer, while the latter two proteins (CRBN, TP53BP) preferentially interact with it.

Important to note is that the ability to detect proteins that preferentially interact with dimeric over monomeric 14-3-3ζ was highly compromised by using 14-3-3ζ pSer58, because its hydrolysis means that the dimeric form will also be present on the column and its partner proteins will be pulled-down. As one example (in addition to the 14-3-3 proteins), BAD was present in similar quantities (by both MS and Western blot) in the WT and pSer58 data sets but was ∼30-fold less abundant in the nhpSer58 pool (Figures 8C and S27). In contrast, as long as some unhydrolyzed pSer-modified protein remained on the column during the experiment, that protein would pull-down proteins that bind monomerized 14-3-3ζ and be useful for identifying such proteins. Since the pull-downs using both the 14-3-3ζ pSer58 and nhpSer58 variants were similarly effective at identifying CRBN and some other proteins as preferential binders, this implies that not all of the phosphorylated 14-3-3ζ pSer58 immobilized on the beads was hydrolyzed during the course of lysate incubation. Nevertheless, that only half as many proteins (27 versus 55) were enriched >8-fold in the pSer58 data set compared to with the nhpSer58 data set highlights the lower efficacy of using natively phosphorylated proteins even for these analyses and underscores the advantage of using the stably phosphorylated 14-3-3ζ variant.

##### Functional Analysis of the Rearranged 14-3-3 Interactome

We used the PANTHER (Protein Analysis Through Evolutionary Relationships) database to group proteins enriched and depleted in the 14-3-3ζ nhpSer58 pull-down data sets by protein type (i.e., class) and the biological pathways in which they function to gain insight into how 14-3-3 monomerization might lead to physiological changes. The top classes of proteins in both the enriched and the depleted pools were protein metabolite interconversion enzymes (PC00262) and protein-modifying enzymes (PC00260) (Figure 8D). Many of these protein-modifying enzymes are directly involved in controlling protein-signaling systems, and some such clients are bound upon monomerization and others are released. For example, several ubiquitin-protein ligase complex proteins (e.g., CRBN, SKP1, FXL12), MAP kinase-signaling proteins (e.g., MAP3K7 and KSR1), and proteases (e.g., HTRA2) were found enriched in the 14-3-3ζ nhpSer58 pull-down (Figure 8E), while several other ubiquitin-protein ligase complex proteins (e.g., TRIM11, DCAF11) and kinases (e.g PAK4, TBK1) were in the depleted pool (Figure 8F). Pathway analysis also identified 24 total proteins involved in “cell death” that were enriched in the 14-3-3ζ nhpSer58 pull-downs (e.g., programmed cell death proteins 4 and 10, TP53BP) and 11 in the depleted pool (e.g., BAD). That BAD is released from 14-3-3ζ upon monomerization provides a direct connection with apoptosis, as the release of BAD allows it to localize to the mitochondrial membrane, leading to BAX/BAK activation, cytochrome-c release, and cell death.^62^

##### Monomeric 14-3-3 Interaction with Cereblon

An intriguing protein identified as an interactor (direct or indirect) of phosphorylated monomeric 14-3-3 was cereblon (CRBN). CRBN acts as a substrate receptor for the E3 ubiquitin ligase complex CRL4^CRBN^ consisting of the ligase scaffold cullin-4 (CUL4), the RING-finger protein RING-box1 (RBX1), and the adapter damage-specific DNA binding protein 1 (DDB1).^63^ When substrate proteins are recruited to CRBN, they are ubiquitinated by the CRL4^CRBN^ complex, marking them for proteosome-mediated degradation. CRBN became a subject of intense immunomodulatory drug development when thalidomide was discovered to bind to and alter CRBN receptor specificity, resulting in the recruitment and degradation of “neosubstrates”.^63^ Proper regulation of CRBN activity and specificity is indeed essential to protein homeostasis and cellular function, but to our knowledge there are no reports of 14-3-3 (monomeric or dimeric) binding to CRBN or the CRL4^CRBN^ complex.

**Figure 9:**
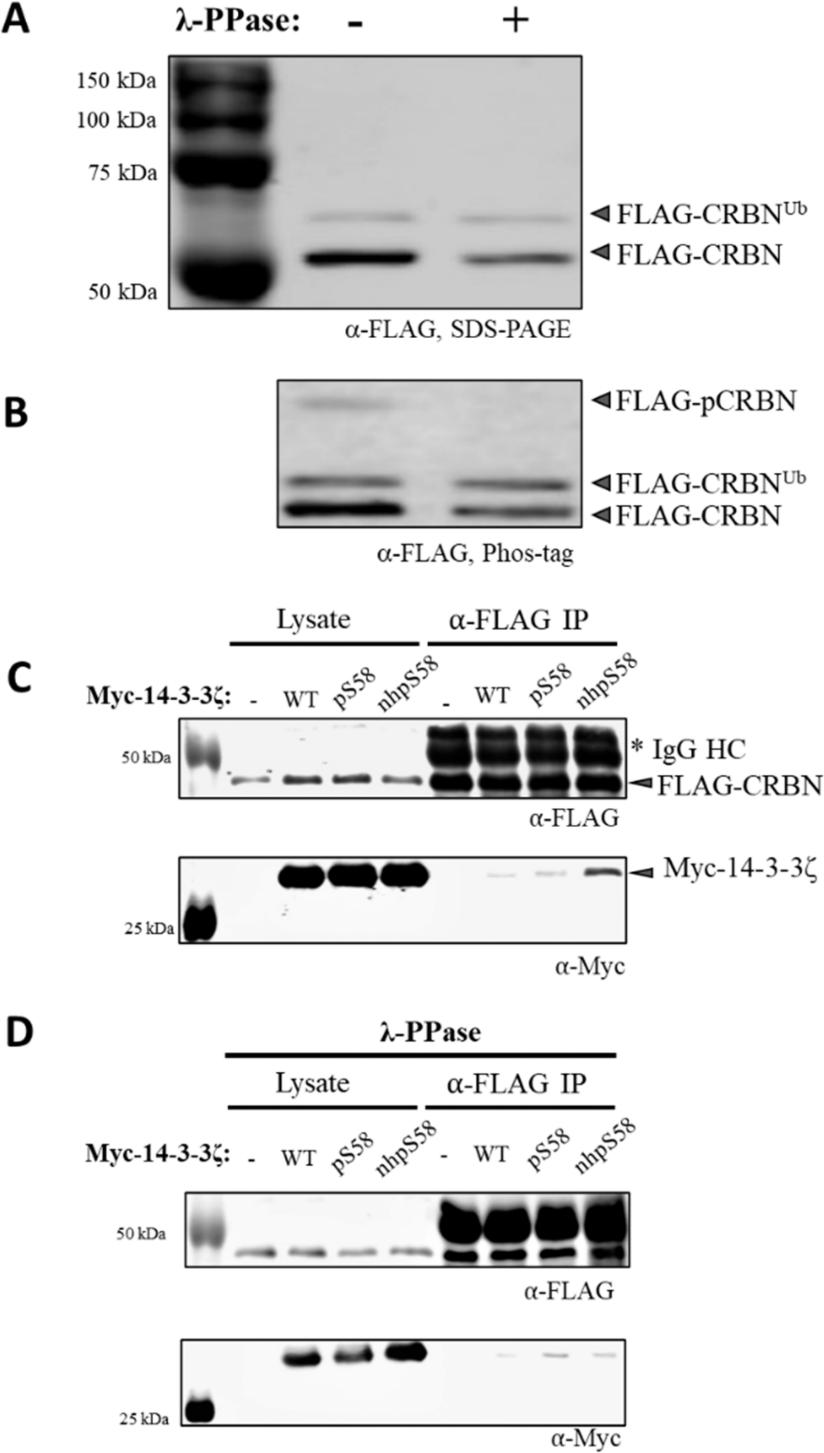
Reciprocal pull-downs confirm interaction of monomeric phosphorylated 14-3-3ζ with CRBN. HEK293T cells expressing FLAG-tagged CRBN were lysed and treated with and without λ-phosphatase, after which these lysates were run on (A) SDS-PAGE and (B) Phos-tag gels and then Western blotted with an α-FLAG antibody. The double banding pattern of CRBN in panel A is consistent with auto-ubiquitination.^63^ The Phos-tag gel in panel B shows a third band in lysates (FLAG-pCRBN) that disappears upon phosphatase treatment, indicating a small population of CRBN is phosphorylated during expression. (C and D) Myc-14-3-3ζ WT, pSer58 and nhpSer58 proteins expressed and purified from *E. coli* were added to these (C) untreated and (D) phosphatase treated HEK293T cell lysates containing FLAG-CRBN, after which α-FLAG resin was added to retrieve the FLAG-CRBN. Resin was washed and FLAG-CRBN was eluted with SDS loading buffer. The lysates as well as the eluted protein pools were run on SDS-PAGE gels and blotted with α-FLAG and α-myc antibodies. Approximately 10-fold more myc-14-3-3ζ nhpSer58 co-immunoprecipitated with CRBN from untreated lysates than myc-14-3-3ζ WT or pSer58 (panel C); this enrichment of myc-14-3-3ζ nhpSer58 was not observed when lysates containing FLAG-CRBN were pretreated with phosphatase (panel D).

Given the relevance of CRBN to disease and pharmacological development, we sought to further explore this interaction between monomeric 14-3-3 and CRBN (or its complex) by carrying out additional pull-down assays, but in “reverse” (Figure 9). To do this, N-terminally FLAG-tagged CRBN was expressed in HEK293T cells, after which the cells were harvested and lysed. Cell lysate was treated with and without λ-phosphatase, and then free (nonimmobilized) myc-tagged 14-3-3ζ proteins (wild-type, pSer58 and nhpSer58 expressed in and purified from *E. coli*) were added. After incubation, anti-FLAG antibody resin was used to retrieve the FLAG-CRBN along with any interacting myc-14-3-3 proteins. Western blots of the eluted proteins using α-FLAG and α-myc antibodies confirmed the isolation of FLAG-CRBN in all samples as expected, while myc-14-3-3ζ was only enriched with the nhpSer58 variant (Figure 9C). The pSer58 variant was not enriched here even though it selectively pulled-down CRBN in the above proteomics analyses (Figures S27B and D); this discrepancy can be explained by pSer58 on free 14-3-3ζ being dephosphorylated during incubation in cellular lysate but being partially protected from phosphatases when immobilized on resin. Interestingly, Phos-tag electrophoresis revealed a subpopulation of FLAG-CRBN to be natively phosphorylated in the lysates, and when treated with λ-phosphatase, this FLAG-CRBN was both dephosphorylated (Figure 9A and B) and lost its ability to pull-down the monomeric 14-3-3ζ nhpSer58 (Figure 9D). These observations confirm an interaction between CRBN (or its complex) with monomeric and phosphorylated 14-3-3ζ and confirm that this interaction is specific for a phosphorylated form of CRBN.

## Discussion

### Advantages and Limitations of PermaPhos

The ability to efficiently and site-specifically install a functional, stable pSer mimic into proteins in *E. coli* opens new doors to study phospho-protein function. We see several advantages of PermaPhos technology compared with existing strategies for producing phosphorylated proteins. First, it leverages GCE technologies for translational incorporation of nhpSer into (in principle) any site of a protein, in contrast to semisynthesis strategies that are generally limited to installing noncanonical amino acids (ncAAs) at the N- or C-termini of proteins. Second, PermaPhos overcomes the bottleneck of poor nhpSer uptake by cells by employing a biosynthetic pathway to produce free nhpSer amino acid from the central metabolite phosphoenolpyruvate, enabling a 40-fold increase in the production of nhpSer-containing proteins. Third, PermaPhos permits the scaling of nhpSer-protein production by removing costs associated with supplementing media with chemically synthesized nhpSer or even any precursor molecules. The reliance on supplementing media with ncAAs has been called the “Achilles’ heel” of GCE, preventing widespread industrial adaptation of GCE technologies, and PermaPhos overcomes that limitation for incorporating nhpSer into proteins.^64^ Finally, in terms of practical applications, the high fidelity of nhpSer as a pSer mimic means that PermaPhos provides a tractable alternative to making authentic pSer-containing proteins for those cases in which possible hydrolysis of the pSer residue hinders the targeted downstream characterizations.

Yet, PermaPhos could benefit from future improvements. For example, the current system was optimized for expression in a Release Factor-1 (RF1)-containing strain of *E. coli*, and so truncated protein is produced alongside full-length nhpSer-containing protein. This means that full-length target protein must be purified away from the truncated protein, which may be difficult even if C-terminal affinity tags can be used (i.e., for oligomeric proteins). Our attempts to adopt PermaPhos in a truncation-free expression host lacking RF1 (e.g., B95(DE3) Δ*A* Δ*fabR* Δ*serC*) resulted in an undesirable amount of natural amino acid mis-encoding at the TAG codon caused by near-cognate suppression (data not shown), a common challenge observed when adopting GCE systems in RF1-deficient cell lines.^65^ In addition, the Frb-v1 pathway operates best at ∼20–30 °C in Terrific Broth media and maximal protein is produced after extended expression times (20–24 h). Target proteins that require expression at temperatures outside this range, in alternative media (e.g., auto-induction or minimal media), or for shorter expression periods may be challenging to express with this initial version of PermaPhos. We also note the last step of nhpSer biosynthesis relies on a yet-to-be identified, endogenous transaminase (Figure 2A). Identifying this transaminase and coupling its overexpression with the current Frb-v1 pathway could afford additional improvements in target nhpSer-protein expression.

### NhpSer as a Mimic for pSer

Common concerns about nhpSer mimicry for pSer are its elevated p*K*_a2_, as well as the loss of the hydrogen bond-accepting oxygen.^45^ The range of p*K*_a2_ values reported for phosphonates prompted us to measure this directly on proteins, and the measured p*K*_a2_ of ∼7.0 implies that a majority of nhpSer molecules will be doubly deprotonated and match the protonation state of pSer at typical slightly basic physiological pH values. Furthermore, the isosteric nature of nhpSer and pSer means that even in protein contexts that raise or lower the p*K*_a2_ of pSer, the p*K*_a2_ of nhpSer should be similarly impacted and the quality of mimicry maintained. We also asked how often the γ-oxygen of pSer is used as a hydrogen bond acceptor in known crystal structures, and we found via an HBPlus^66^ analysis that of the 171 unique structurally known proteins (<75% sequence identity) with pSer that the γ-oxygen of pSer was involved in a putative hydrogen bond with a protein atom in only 35 of them (∼20%) (Supporting Data Set 2). Major pSer-binding protein families 14-3-3, BRCA1 C-terminal (BRCT) and WW domains (named for their two invariant tryptophan residues) do not use the γ-oxygen of pSer as a hydrogen bond acceptor to stabilize pSer-dependent interactions (Figure S28). In contrast, major phosphotyrosine-binding protein families, such as Src-homology 2 (SH2) and phosphotyrosine binding (PTB) domains, do make key interactions with the bridging oxygen of pTyr (Oη) in pTyr-dependent interactions^67^ and, indeed, peptides containing the analogous nonhydrolyzable pTyr mimic with a bridging CH_2_ group have markedly reduced affinities to SH2 and PTB proteins compared to the same peptides with pTyr.^68^

To experimentally test the extent to which nhpSer mimics the function of pSer, we carried out side-by-side comparisons of nhpSer- and pSer-protein function with three stringent test cases: the priming of GSK3β kinase activity, 14-3-3/HSPB6 phosphoserine-dependent complexation, and pSer-dependent monomerization of 14-3-3. For all three cases, we verified that nhpSer-protein functioned similarly to the same proteins with pSer, and no noteworthy differences between nhpSer- and pSer-protein were detected. Importantly, in all three cases proteins with Asp or Glu placed at the same sites failed to mimic the key functional properties of the pSer-containing proteins. That nhpSer can substitute for pSer to stabilize phosphorylation-dependent 14-3-3/client complexes creates new opportunities to study them in cellular environments. Whereas nhpSer is—compared to Asp or Glu—a much better mimic for pSer, it is not identical to pSer and so it must be expected that detailed analyses will in some cases reveal subtle structural and/or functional discrepancies in its behavior. For instance, in a recent elegant study by Stuber et al.,^69^ while a nhpSer65 variant of ubiquitin did adopt the functionally important pSer-dependent conformational state that was not adopted by the Asp/Glu variants, it did so with a lower occupancy than authentically phosphorylated ubiquitin. The authors speculated that a difference in nhpSer and pSer charge states could be causing the disparity; however, given our p*K*_a2_ measurement of nhpSer, the large majority of nhpSer should still be dianionic like pSer, and so additional mechanisms may be at play.

### Using PermaPhos to Study the Function of Phosphorylated, Monomeric 14-3-3ζ

Connecting specific PTMs on 14-3-3 with defined changes in function remains a major challenge. With regard to the impacts of 14-3-3ζ phosphorylation at Ser58, it is known to monomerize 14-3-3, but without well-established methods to make stably phosphorylated protein, prior work to study the function of Ser58 phosphorylation has largely relied on introducing a variety of point mutations at the dimer interface of 14-3-3, including phospho-mimicking Ser → Asp/Glu mutations that do not fully monomerize 14-3-3.^17,20^ While these studies have helped uncover how the dimeric versus monomeric state of 14-3-3 impacts the binding and regulation of a few select clients, the results were often not conclusive; they also left unresolved the role phosphorylation *per se* plays in altering 14-3-3 function, since the phosphate group at Ser58 could be directly involved in client interactions, and/or impact the conformation of monomeric 14-3-3. To help resolve these ambiguities, we used GCE to express both 14-3-3ζ pSer58 and nhpSer58 and confirmed their monomeric nature (Figure 7C). While we could have used them to study the binding properties of select clients, we sought for the first time to study client-binding effects at the interactome level. As anticipated, dephosphorylation of the pSer58 protein in cell lysates made those results less informative. On the other hand, the 14-3-3ζ nhpSer58 variant was resistant to dephosphorylation (Figure S26) and maintained a functional phospho-peptide-binding groove (Figure S25), allowing us to cleanly compare the interactomes of the phosphorylated monomeric versus the wild-type dimeric 14-3-3ζ.

These data provide the first clear glimpse into how this key PTM on 14-3-3 regulates its interactome; whereas most clients bound both WT and nhpSer58 variants similarly, a defined subset did not bind monomeric 14-3-3 (e.g., BAD) and another subset preferentially bound the phosphorylated, monomeric form (e.g., cereblon). Thus, the interactome of 14-3-3ζ undergoes a defined rearrangement when it is phosphorylated at Ser58. Comparing these data to prior work revealed little overlap regarding client-specific changes in 14-3-3 binding, which may not be surprising given the advantages of the stability and improved pSer mimicry of nhpSer. For example, we found vimentin and BAX bound dimeric and phosphorylated monomeric 14-3-3ζ similarly, in contrast to prior work indicating monomerization of 14-3-3 (via interfacial point mutations) should release both.^70,71^ Further, we found that Kinase Suppressor of Ras1 (KSR1) preferentially bound phosphorylated monomeric over dimeric 14-3-3ζ, in contrast to prior work using a Ser → Asp mutation to mimic the phosphorylation.^72^ Besides the types of mutations used to monomerize 14-3-3, additional factors such as the cell types and 14-3-3 isoforms used could contribute to the discrepancies.

Nevertheless, the snapshots of 14-3-3ζ interactomes presented here along with the ability to use nhpSer -containing 14-3-3 variants in future studies provide a foundation that will open the door to new research avenues and lead to a better understanding of how phosphorylation of 14-3-3 alters cellular signaling systems. A new observation in this regard was the identification that monomeric, phosphorylated 14-3-3ζ bound to CRBN (directly or indirectly); this complex was observed in the proteomic analyses of both biological replicate pull-downs as well as in reciprocal CRBN pull-downs (Figures 9 and S27). CRBN’s binding to phosphorylated monomeric 14-3-3ζ could impact its ability to act as a substrate adaptor protein for the CRL4^CRBN^ ubiquitination complex by changing its specificity/activity. Or, monomeric 14-3-3ζ with its exposed dimerization interface could bind CRBN to make 14-3-3ζ (and/or its bound clients) targets for ubiquitination. The latter possibility is consistent with prior work that suggested monomeric 14-3-3 has a shorter lifetime in cells than dimeric 14-3-3.^73^ We also found this monomeric 14-3-3ζ/CRBN interaction was markedly reduced when lysates were pretreated with phosphatase and CRBN was dephosphorylated. The most commonly detected site of phosphorylation on cereblon is Ser25,^9^ but Ser25 is not located within an obvious 14-3-3 binding motif and we found no prior work investigating how Ser25 phosphorylation impacts CRL4^CRBN^ function. The discovery that monomeric 14-3-3ζ forms a complex with CRBN (or CRL4^CRBN^) brings to light new mechanisms by which protein ubiquitination and 14-3-3 signaling pathways can be mediated, and it makes possible new strategies that leverage these inducible interactions for targeted protein degradation therapies. Prior studies suggested monomerization of 14-3-3 is a key step in priming cells for apoptosis under oxidative stress conditions,^11,62,74,75^ and so it is plausible monomeric phosphorylated 14-3-3 plays an important role in altering ubiquitination and protein degradation systems as part of this transition, in conjunction with the release of BAD from 14-3-3.

### Outlook

PermaPhos provides a tractable and scalable strategy for producing site-specific, permanently phosphorylated proteins in *E. coli*. This access to nhpSer proteins should expand opportunities to discover new phosphorylation-dependent mechanisms of protein regulation. With seven isoforms of 14-3-3 that each have at least 20 other known sites of Ser/Thr phosphorylation, the door is open to similar interactome analyses that address long-standing questions about how these PTMs affect 14-3-3 signaling systems. Such strategies can be easily extended to other phosphorylated proteins as well. Finally, the PermaPhos system will also enable transfection and microinjection studies of *E. coli* produced nhpSer proteins into eukaryotes as an avenue to more effectively learn how specific phosphorylation events regulate signaling systems *in vivo*.

## Methods

Brief methods are described below. More detailed descriptions are provided in the Supporting Information.

### Strains

BL21(DE3) Δ*serB* was a gift from Jesse Rinehart. BL21(DE3) Δ*serC* was a gift from Jason Chin and was also made in-house using standard λ-red recombineering.^76^ B95(DE3) Δ*A* Δ*fabR* Δ*serB* was generated at the OSU Unnatural Protein Facility as previously described.^22^ DH10b was purchased from Thermo Fisher Scientific. The PPY strain used to generate SLiCE^77^ cloning extract was a gift from Yongwei Zhang (Albert Einstein College of Medicine).

### Molecular Biology Reagents

Oligonucleotide primers and double stranded DNA fragments were synthesized by Integrated DNA Technologies (Coralville, IA). Molecular biology reagents including restriction enzymes, T4 ligase and polymerases were purchased either from Thermo Fisher Scientific or New England Biolabs. DNA Miniprep, Midiprep, PCR cleanup and gel extraction kits were purchased from Machery Nagel.

### Generating the Frb-v1 nhpSer Biosynthetic Pathway

#### Selection of Attenuated T7 Promoters

A library of mutant T7 promoters with altered transcriptional activity was generated and placed in front of a fluorescent reporter sfGFP gene. Mutant promoters with targeted transcriptional efficiency were selected in a similar manner as previously described, except that the expression strain was BL21(DE3) Δ*serC*.^42^

#### FrbABCDE Library Assembly

The FrbABCDE library assembly strategy was adapted by Zhao and colleagues^42^ such that all permutations of attenuated transcriptional promoters in front of each of the five Frb genes were made via Golden Gate assembly (5^5^ = 3125 combinations). Each gene was flanked by its own promoter and terminator so that each is transcribed independently. The plasmid backbone contained a CDF origin of replication and spectinomycin resistance to ensure compatibility and stable propagation with the nhpSer GCE machinery (pUC origin/kanamycin resistance) and target protein expression plasmids (p15a origin/ampicillin resistance).

#### Screening for Functional FrbABCDE Assemblies

The pCDF-FrbABCDE_lib was screened for assemblies able to synthesize nhpSer for translational incorporation into sfGFP-150TAG. To do this, the pCDF-FrbABCDE_lib was electroporated with the nhpSer GCE machinery plasmid, pSF-nhpSer (see below), and the reporter expression plasmid, pRBC-sfGFP-150TAG, into BL21(DE3) Δ*serC* cells. Cells were plated on inducing LB/agar plates, and 92 top fluorescent clones were selected. Plasmid DNA of the top 6 clones were isolated and pathway functionality was retested in subsequent expressions at 50 mL scale, in triplicate. Promoter strengths were identified by sequencing.

### Quantification of sfGFP Expression

Yield of sfGFP expressed per liter culture was calculated by measuring the in-cell fluorescence of sfGFP and subtracting the contribution of cell autofluorescence (measured from the same density of cells not expressing any sfGFP construct). Fluorescence values were converted to mass of sfGFP per liter culture based on a standard curve of purified sfGFP. All values reported are the average of three independent replicate cultures, and error bars represent the standard deviation.

#### Protein Expression and Purification

Detailed expression and purification protocols for each target protein can be found in the Supporting Information. Brief descriptions for expressing pSer and nhpSer proteins are described below. Sequences of each expressed protein are also provided in the Supporting Information.

#### nhpSer-Protein Expression

The pSF-nhpSer machinery plasmid for selective nhpSer incorporation was a kind gift from Jason Chin.^34^ A modified form of this plasmid (pERM-nhpSer, Kan resistance, pUC origin of replication) was built with additional levels of transcriptional insulation to ensure independent expression of each component: SepRS-2^33^ expression was controlled by a constitutive Gln *R*S promoter and a rrnB T1 transcriptional terminator, the Sep-tRNA^v2.0^_CUA_^35^ controlled by a constitutive lpp promoter and rrnC terminator, and the EF-Sep^38^ controlled by a lac promoter and T7 terminator, and an *E. coli serB* was expressed under a strong constitutive oxb20 promoter and rrnG terminator.

##### Expression Using nhpSer Media Supplementation

Fresh transformations were performed for all expressions in this study. Expressions with nhpSer media supplementation were performed essentially as previously described.^33^ Briefly, BL21(DE3) Δ*serC* cells were cotransformed with pRBC-sfGFP-150TAG or pRBC-sfGFP-134/150TAG and pSF-nhpSer (or pERM-nhpSer). Cells were grown and protein expressed in 2xYT media induced with the addition of 1 mM IPTG and 2 mM nhpSer (DL-AP4, Abcam) at 37 °C for 18 h.

##### Expression Using the Frb-v1 Pathway

The pCDF-Frb-v1.0, pSF-nhpSer (or pERM-nhpSer) and the appropriate pRBC plasmid were cotransformed simultaneously by electroporation into BL21(DE3) Δ*serC* cells. Expressions were performed in Terrific Broth (TB: 12 g/L tryptone, 24 g/L yeast extract, 0.5% glycerol, 72 mM K_2_HPO_4_, 17 mM KH_2_PO_4_) and initiated by the addition of 0.5 mM IPTG once cells reached an OD_600_ ∼ 1.0. Cultures were harvested 20–24 h after the addition of IPTG.

### pSer-Protein Expression

The pKW2-EFSep machinery plasmid (a kind gift from Jason Chin) was used for pSer incorporation, which expresses SepRS-2 under a constitutive Gln *R*S promoter, the Sep-tRNA_CUA_ G4 under a constitutive lpp promoter, and the EF-Sep under the control of a lac promoter.^33^ Proteins with pSer were expressed exactly as previously described^22^ in either BL21(DE3) Δ*serB* or B95(DE3) Δ*A* Δ*fabR* Δ*serB*. The latter, which is a partially recoded derivative of BL21(DE3),^78^ lacks Release Factor-1 and therefore was used to avoid truncated protein.^22^

#### λ-Phosphatase Hydrolysis

8.3 μM *bd*SUMO-HSPB6^11−20^ fusion protein containing pSer or nhpSer at site S16 of HSPB6 was reacted with 3 units of λ phosphatase (NEB) according to the manufacturer’s guidelines. Reactions were quenched at the indicated time points by acetone precipitation. Precipitated proteins were pelleted at 20k rcf at 4 °C, dried and resuspended in 1× SDS sample buffer for SDS and Phos-tag gel analysis.

#### ^31^P Nuclear Magnetic Resonance (NMR)

*bd*SUMO-HSPB6^11−20^ proteins in 25 mM Tris pH 7.4, 25 mM NaCl at 5 mM concentration were diluted 10-fold in a buffer with pH ranging from 4 to 10. This dilution buffer contained 100 mM sodium acetate (p*K*_a_ 4.8), 100 mM Bis-Tris (p*K*_a_ 6.5), 100 mM Tris (p*K*_a_ 8.1), 100 mM CHES (p*K*_a_ 9.3), 25 mM NaCl, and 10% D_2_O, and its pH was between 4 and 10. By including four reagents with p*K*_a_’s ranging from 4.8 to 9.3, buffering was effective from pH 4 to 10. Thus, all proteins for NMR analysis, regardless of pH, were in the identical buffering matrix. After dilution, any insoluble protein was precipitated by centrifugation. Samples were then transferred to 5 mm NMR tubes (Norell) and experiments were conducted on a Bruker Advance III 500 MHz NMR Spectrometer outfitted with a 5 mm BBOF probe. Data were collected with a spectral window of 99.5774 ppm, 65,536 real plus imaginary points, a D1 of 2.0 s, and between 1,024 and 13,000 scans. All NMR experiments were collected at 25 °C. NMR data were processed (apodized, zero filled, Fourier transformed, and phased) and analyzed in Bruker Topspin. Labeled peaks were exported to Graphpad Prism 8 and plotted against the pH of the buffer; the plotted points were interpolated to a Sigmoidal, 4 PL function, and the p*K*_a2_ was determined at the calculated inflection point ±95% Confidence Interval.

#### Sedimentation Equilibrium Analytical Ultracentrifugation (AUC)

Protein samples for AUC were serially diluted to an Abs at 280 nm of 1.0 (14.3 μM), 0.5 (7.2 μM), and 0.25 (3.6 μM) into 50 mM Tris pH 7.4, 150 mM NaCl, 1 mM TCEP. AUC experiments were performed using a Beckman Coulter Optima XL-A ultracentrifuge equipped with absorbance optics (Brea, CA). Protein-partial-specific volumes as well as buffer densities and viscosities were estimated using the software sednterp.^79^ Experiments were performed using an An-60-ti rotor at three speeds: 8,000, 10,000, and 15,000 rpm all at 5 °C. Scans at a wavelength of 280 nm were taken every 3 h to check that equilibrium had been achieved, with final scans taken a minimum of 30 h after reaching speed. Data were fit to a simple heterodimerization model (A + B ↔ AB) using fixed masses of 33031 Da for a HSPB6 dimer and 52395 Da for a 14-3-3ζ dimer with the Heteroanalysis software (version 1.1.60, University of Connecticut). Proteins were loaded on SDS-PAGE and Phos-tag gels before and after AUC experiments to confirm sample integrity was maintained during data acquisition. Calculated ln *K*_a_ values for pSer- and nhpSer-containing complexes were compared by student’s unpaired two-tailed *t* test (*p* = 0.05) to determine statistical significance.

#### Size Exclusion Chromatography Coupled to Multiangle Light Scattering (SEC-MALS)

Experimental molecular weights were obtained by size exclusion chromatography (SEC) using an AKTA FPLC (GE Healthcare) coupled to a DAWN multiangle light scattering (MALS) and Optilab refractive index system (Wyatt Technology). Size exclusions were conducted on a Superdex 200 10/300 GL column (Cytiva Life Sciences) pre-equilibrated in 50 mM Tris pH 7.4, 150 mM NaCl, 0.5 mM TCEP at room temperature. Protein samples were prepared at 50 μM and injected at a flow rate of 0.8 mL/min. Duplicate data sets were analyzed using the ASTRA software package, version 8 (Wyatt Technology).

#### GSK3β Kinase Reactions

80 μM of target protein (Linker Np-sfGFP or full-length Np) was incubated with 80 nM of GSK3β in a buffer containing 50 mM Tris pH 7.4, 150 mM NaCl (350 mM NaCl for Linker Np-sfGFP), 10 mM MgCl_2_, and 1 mM ATP. Reactions were incubated at 37 °C for 20 h. Reactions were then quenched for analysis by boiling in 1× SDS sample buffer and loaded on SDS-PAGE and Phos-tag gel, or frozen at −20 °C in preparation for mass spectrometry.

### Mass Spectrometry of Phosphorylated sfGFP

#### Intact sfGFP

A Waters nanoAcquity UPLC system (Waters, Milford, MA) was coupled online to an Orbitrap Fusion Lumos Tribrid ETD mass spectrometer (Thermo Fisher Scientific). Protein samples were diluted 25 times in 0.1% formic acid. Proteins were loaded onto a trap 2G nanoAcquity UPLC Trap C4 Column (180 μm, 50 mm, 5 μm, 300 Å) at a flow rate of 5 μL/min for 5 min. The separation was performed on an Acquity UPLC BEH C4 column (100 μm, 100 mm, 1.7 μm, 300 Å). Column temperature was maintained at 37 °C using the integrated column heater. Solvent A was 0.1% formic acid in LC-MS grade water, and solvent B was 0.1% formic acid in LC-MS grade acetonitrile. The separation was performed at a flow rate of 0.5 μL/min, and using linear gradients of 3–10% B for 3 min, 10–30% B for 17 min, 30–90% B for 3 min, 90% B for 4 min, 95–3% B for 1 min, and 3% B for 7 min. The total method length was 35 min. The outlet of the column was connected to the Thermo Nanospray Flex ion source, and +2300 V was applied to the needle.

The MS acquisition method was optimized for the target sfGFPs. The optimum setting for in-source dissociation was found to be 15%. Full MS spectra were acquired over *m*/*z* 400–2000 because this mass range contained all charge states observed for the target proteins. Scans with resolution 240000 at *m*/*z* 200 with accumulation of 10 microscans were sufficient to resolve the isotopic distribution.

Raw data were deconvoluted using UniDec software.^80^ The charge range and mass range were set 5 to 50 and 5 kDa to 50 kDa, respectively. The automatic *m*/*z* peak width was determined to be ∼0.07 Th. Split Gaussian/Lorentzian was selected as the peak shape function. Deisotoping was switched off. Exported deconvoluted spectra for different files were juxtaposed in OriginPro 2021 (v9.8).

#### Trypsin Digestion

Protein samples were diluted 25-fold in 50 mM ammonium bicarbonate. To prevent disulfide bonds of the proteins, the samples were incubated at 56 °C for 1 h with 5 mM dithiothreitol (ThermoFisher). Then, the samples were incubated with 10 mM iodoacetamide (MilliPore Sigma) for 1 h at room temperature in the dark in order to carbamidomethylate cysteine residues. Samples were digested overnight at 37 °C using Trypsin Gold (Mass Spectrometry grade, Promega). After digestion, samples were spun down at 12000 rcf for 30 s to collect condensate, and the digestion was stopped by addition of 0.5% (v/v) trifluoroacetic acid. Samples were centrifuged at 12000 rcf for 10 min and then transferred to LC vials.

#### Bottom-up Mass Spectrometry

Peptides were loaded onto a trap 2G nanoAcquity UPLC Trap C18 Column (180 μm, 50 mm, 5 μm, 130 Å) at a flow rate of 5 μL/min for 5 min. The separation was performed on a commercially available Acquity UPLC Peptide BEH C18 column (100 μm, 100 mm, 1.7 μm, 130 Å). The LC gradient and other parameters were identical to those of the method described above for the top-down analysis.

MS1 spectra were acquired at a resolution of 120,000 (at *m*/*z* 200) in the Orbitrap using a maximum IT of 50 ms and an automatic gain control (AGC) target value of 2E5. For MS2 spectra, up to 10 peptide precursors were isolated for fragmentation (isolation width of 1.6 Th, maximum IT of 10 ms, AGC value of 2e4). Precursors were fragmented by electron transfer dissociation (ETD) and analyzed in the Orbitrap at a resolution of 30,000 at *m*/*z* 200. Reagent injection time and other ETD parameters were set to be selected automatically based on the calibration parameters. The dynamic exclusion duration of fragmented precursor ions was set to 30 s.

Raw files were processed in Thermo Proteome Discoverer 2.3. Precursor ion mass tolerance was set to 5 ppm, while fragment ion mass tolerance was 0.02 Da. The SequestHT search engine was used to search against the database containing target sfGFP sequences and variation databases. Variation databases were created for the incorporation sites to test for noncognate incorporation. Additional dynamic modifications were created for aspartate and asparagine conversion into phosphoserine (+51.9714, + 52.9554) and nonhydrolyzable phosphoserine (+49.9922, +50.9672). c and z ions only were considered for peptide spectrum matching. MS1 precursor quantification was used for label-free quantitation of the peptides.

### Mass Spectrometry of Linker-Np

Amicon Ultra 3000 Da cutoff centrifugal filter units were used to buffer exchange Linker-Np samples into 200 mM ammonium acetate, after which the proteins were diluted to 10 μM in 15% acetonitrile and 0.1% formic acid. All samples were analyzed on an Agilent 6545XT equipped with an e-MSion ExD cell (model AQ-250) and ionized through direct infusion into a dual AJS electrospray source or sprayed with a custom static nano electrospray source. The instrument was operating in dynamic range mode (2 GHz), and spectra were collected at a rate of 1 spectrum per second. A scan range of 400–2,400 *m*/*z* was used to detect intact masses in MS1 mode and 120–2,400 *m*/*z* in MS2. The Linker-Np precursors were isolated and fragmented through a targeted acquisition run. Electron capture dissociation (ECD) was used to fragment the gas phase protein ions. An additional 10 V of collision energy was required to fragment the multiply phosphorylated Linker-Np proteins. MS2 fragmentation spectra were acquired and averaged for approximately 6 min.

The Agilent Mass Hunter BioConfirm B.09 software was used to deconvolute the *m*/*z* spectra and determine the intact protein masses. Fragmentation spectra were analyzed with ExD Viewer v4.1.14. The fragment ions were compared to theoretical fragmentation data based on the known sequence of Linker-Np. Fragment ions were matched by considering the experimental mass to charge values and isotopic distribution. The maximum error allowed for mass to charge matching was 20 ppm. The experimental spectra were searched for b, y, c, and z ions, corresponding to ECD and CID-type product ions. The presence of a phosphorylation was determined by a mass shift of +79.9 relative to the theoretical mass. Non-hydrolyzable phosphoserine moieties were identified by matching the theoretical methyl phosphonate fragment in position S188 or S206.

#### Mammalian Cell Culture

##### HEK293T Cultures for Proteomics Studies

HEK293T cells were seeded onto 16 × 150 mm plates containing DMEM (Corning, 10-017-CV) at a density of ∼8 × 10^6^ cells per plate and were allowed to grow until ∼90% confluency. ∼5 × 10^8^ cells total were harvested from these plates, pooled, and flash frozen with liquid N_2_. Cell pellets were stored at −80 °C until 14-3-3ζ affinity enrichment experiments.

##### Expression of FLAG-Cereblon in HEK293T Cells

HEK293T cells were seeded into 2 × 150 mm plates containing DMEM and were grown overnight at 37 °C at 95% humidity. Cells were then transfected with a pAcBac plasmid containing an N-terminally FLAG-tagged human cereblon (CRBN) under a CMV promoter. Transfections were carried out using jetPRIME transfection reagent (Polyplus, 101000015) according to the manufacturer’s guidelines. ∼4 × 10^8^ cells were harvested 16–18 h post transfection and frozen with liquid N_2_ until used in co-immunoprecipitation assays later.

#### Affinity Enrichment Proteomics

##### Preparation of HEK293T Lysate

HEK293T lysate was prepared by sonication of ∼5 × 10^8^ HEK293T cells resuspended in 5 mL of 50 mM Tris pH 7.5, 150 mM NaCl, and 50 nM Calyculin A.

##### 14-3-3ζ Affinity Enrichment

NHS-Sepharose was cross-linked to 14-3-3ζ proteins (wild-type, pSer58 and nhpSer58) at 50 μM concentration according to the manufacturer’s guidelines (Cytiva) in 50 mM HEPES pH 8.5, 150 mM NaCl for 2–3 h with rocking at 25 °C; as a blank, NHS-Sepharose was coupled to Tris (10 mM) supplemented in the same buffer. Coupling was quenched with 1 M ethanolamine. Columns were then washed and equilibrated in 50 mM Tris pH 7.5, 150 mM NaCl, 50 nM Calyculin A and then incubated with HEK293T lysate for 2 h at 25 °C. Columns were washed with 100 × bed volume of 50 mM Tris pH 7.5, 350 mM NaCl, 50 nM Calyculin A. Columns were then eluted with 1.4 × bed volume of 6 M Guanidine-HCl, and the liquid phase was ethanol-precipitated by the addition of EtOH to a final concentration of 95%. Proteins were then pelleted, dried and stored at −80 °C until mass spectrometry experiments. Affinity enrichments were performed in biological duplicates. Biological replicate 1 was done with 80 μL of bed volume of NHS-Sepharose, and biological replicate 2 was performed using 400 μL of bed volume.

##### Affinity Enrichment Mass Spectrometry and Data Analysis

After ethanol precipitation, the protein pellets were dissolved in 50 mM ammonium bicarbonate solution containing 0.1% Rapigest and 5 mM dithiothreitol (Thermo Fisher Scientific) and were incubated at 65 °C for 1 h in order to reduce the disulfide bonds. Then, the samples were incubated with 10 mM iodoacetamide (MilliPore Sigma) for 45 min at RT in the dark to irreversibly alkylate cysteine residues. Samples were digested overnight at 37 °C using Mass Spectrometry grade Trypsin Gold (Promega). After digestion, samples were spun down at 12,000*g* for 30 s to collect the condensate, and neat trifluoroacetic acid was added to 0.5% (v/v) in order to stop the digestion and to degrade the Rapigest surfactant, and the samples were incubated at 37 °C for 45 min. Samples were centrifuged at 12,000*g* for 10 min, and the supernatant was transferred to LC vials.

All samples were analyzed by mass spectrometry in triplicate. For each analysis, a Waters nanoAcquity UPLC system (Waters, Milford, MA) was coupled online to an Orbitrap Fusion Lumos Tribrid ETD mass spectrometer (Thermo Fisher Scientific). Peptides were loaded onto a trap 2G nanoAcquity UPLC Trap C18 column (100 mm × 50 mm × 5 μm) at a flow rate of 5 μL/min for 5 min. The separation was performed on a commercially available Acquity UPLC Peptide BEH C18 column (100 μm × 100 mm × 1.7 μm). The column temperature was maintained at 37 °C using the integrated column heater. Solvent A was 0.1% formic acid in LC-MS grade water, and solvent B was 0.1% formic acid in LC-MS grade acetonitrile. The separation was performed at a flow rate of 0.5 μL/min using linear gradients of 3–10% B for 3 min, 10–30% B for 102 min, 30–90% B for 3 min, steady 90% B for 1 min, followed by 90–3% B for 3 min, and steady 3% B for 8 min. The total method length was 120 min. The outlet of the column was connected to the Thermo Nanospray Flex ion source, and +2300 V was applied to the stainless-steel needle.

MS1 spectra were acquired at a resolution of 120,000 (at *m*/*z* 200) in the Orbitrap using a maximum IT of 50 ms and an automatic gain control (AGC) target value of 1E5. For MS2 spectra, up to 8 peptide precursors were isolated for fragmentation (isolation width of 1.6 Th, maximum IT of 10 ms, AGC value of 1e4). Precursors were fragmented using higher energy C-trap dissociation (HCD) with 32% collision energy and analyzed in the Orbitrap at a resolution of 30,000 at *m*/*z* 200. The dynamic exclusion duration of fragmented precursor ions was set to 60 s. Raw files were processed in Thermo Proteome Discoverer 2.4. Precursor ion mass tolerance was set to 5 ppm, while fragment ion mass tolerance was 0.02 Da. The SequestHT search engine was used to search against the database consisting of the Swissprot Human reference proteome (UP000005640) and an in-house contaminant database. Methionine oxidation was set as a dynamic modification. Acetylation, Methionine-loss + Acetylation, and Methionine-loss were set as protein N-terminal dynamic modifications. Carbamidomethylation of cysteines was assigned as a static modification. b- and y-ions only were considered for peptide spectrum matching. MS1 precursor quantification was used for label-free quantitation of the peptides. The protein and peptide false discovery rate (FDR) was set to 0.01% (high confidence). Further statistical analysis was conducted using MetaboAnalyst version 5.0 (metaboanalyst.ca). Missing values of protein abundances were imputed using the 1/5 Limit of Detection (LoD) protocol on Metaboanalyst, and protein abundances with relative standard deviation (RSD) > 20% were excluded from analysis. Volcano plots comparing affinity enrichment from wild-type (WT), pSer58 and nhpSer58 14-3-3ζ columns were generated using MetaboAnalyst. All data points were exported from the MetaboAnalyst Web server and graphed using GraphPad Prism. Nonredundant gene IDs corresponding to enriched proteins (log_2_(fold-change) > 1.5, −log(p-value) > 1.31) and depleted proteins (log_2_(fold-change) < −1.5, −log(p-value) > 1.31) were exported from the Thermo Proteome Discoverer software and annotated using the Panther Database. Proteins which were enriched in one biological replicate but depleted in another were omitted from analysis by Panther.

#### Co-immunoprecipitation of FLAG-CRBN and 14-3-3ζ

##### Preparation of HEK293T Lysate Containing FLAG-Cereblon and Myc-14-3-3ζ Variants

∼4 × 10^7^ HEK293T cells having expressed N-terminally FLAG-tagged cereblon from a CMV promoter for 24 h were resuspended in 3.5 mL of 50 mM Tris pH 7.5, 150 mM NaCl and lysed by sonication. Insoluble cell debris was pelleted at 21,000 rcf for 20 min. Clarified HEK293T cell lysates were then partitioned into two halves: to one half, Calyculin-A (50 μM) was added, and to the other, 0.1 μM λ-phosphatase (NEB) and 10 mM MnCl_2_ were added, and the mixture was incubated for 2 h.

##### Co-immunoprecipitation of FLAG-Cereblon and Myc-14-3-3ζ Variants

20 μM Myc-14-3-3ζ (WT, pS58 or nhpS58) was added to 400 μL of HEK293T lysates prepared as described above. Myc-14-3-3ζ variants were allowed to incubate with the lysate for 1 h. Then, 35 μL bed volume of ANTI-FLAG M2 Affinity Gel (Sigma-Aldrich, A2220) equilibrated with 50 mM Tris pH 7.5, 150 mM NaCl was added to each sample. After incubation, the ANTI-FLAG resin was washed with 5 × 500 μL of 50 mM Tris pH 7.5, 150 mM NaCl. Proteins were eluted by boiling in 35 μL of 1 × Laemli’s buffer for 5 min, and the resulting supernatant was used for analysis by Western blot.

#### Western Blots

##### General Procedure for Western Blots

Samples were run on SDS-PAGE Phos-tag gels. Prior to transfer, Phos-tag gels were washed 3 times with 10 mL of 10 mM EDTA and 1 times with transfer buffer. Gels were then transferred onto Immobilon-FL PVDF membranes (Millipore, IPFL00010) using the wet transfer method in transfer buffer (25 mM Tris, 192 mM Glycine, 20% methanol pH 8.6) at 30 V, overnight in 4 °C. After transfer, membranes were blocked with 5 mL of Intercept TBS Blocking Buffer (LI-COR, 927-60001) for 1.5 h at 25 °C. All primary and secondary antibodies were diluted into a 50:50 mixture of TBST (20 mM Tris pH 7.5, 150 mM NaCl, and 0.1% Tween-20) and Intercept TBS Blocking Buffer. Membranes were washed 3 times after primary antibody incubation, and 4 times after secondary antibody incubation for 10 min per wash. All secondary antibodies were diluted 1:10000 prior to use and incubated with the membrane for 1 h at 25 °C. All Western blots were imaged on a LI-COR Odyssey Clx with Image Studio version 5.2 using infrared detection at 700 nm wavelength.

##### Detection of Proteins from 14-3-3ζ Affinity Enrichment

PVDF membranes were incubated with (1:500 dilution) Anti-BAD Antibody conjugated to Alexa-Fluor 680 (C-7) (Santa Cruz Biotechnology, sc-8044 AF680), (1:500 dilution) Anti-TP53BP2 Antibody conjugated to Alexa-Fluor 680 (Santa Cruz Biotechnology, sc-398311 AF680), (1:500 dilution) Anti-pan14-3-3 Antibody (Santa Cruz Biotechnology, sc-1657), and (1:1000 dilution) Anti-Cereblon Antibody (Cell Signaling Technology, D8H3S) overnight at 4 °C. Alexa-Fluor conjugated antibodies were directly detected on the blots, after overnight incubation following washing of the membrane. Blots treated with either Anti-pan14-3-3 Antibody or Anti-Cereblon Antibody were washed after overnight incubation and then incubated with IRDYE 680RD Goat Anti-Mouse Secondary Antibody (LI-COR, 926-68070) or IRDYE 680RD Goat Anti-Rabbit Secondary Antibody (LI-COR, 926-68071).

##### Stability Assessment of Phosphorylated 14-3-3ζ in HEK293T Lysate

FLAG-14-3-3ζ variants (WT, pS58 or nhpS58) were added to HEK293T lysate to a final concentration of 1 μM. Samples were taken at time 0 (directly after addition of FLAG-14-3-3ζ proteins) or after 120 min of incubation with lysate at 25 °C. 2 μL of each sample was loaded onto SDS-PAGE and Phos-tag gel and transferred onto PVDF membranes. After transfer, membranes were incubated with Anti-FLAG M2 Antibody (Sigma-Aldrich, F3165) (1:1000 dilution) for 1 h at 25 °C. After incubation with primary antibody, membranes were washed and incubated with IRDYE 680RD Goat Anti-Mouse Secondary Antibody for 1 h at 25 °C.

##### Assessment of FLAG-Cereblon Phosphorylation Status

Lysates (with and without λ-phosphatase treatment) were prepared as described above from cells expressing FLAG-Cereblon and the subjected to SDS-PAGE and Phos-tag electrophoresis. Gels were transferred to PVDF membranes and probed with the same Anti-FLAG M2 Antibody as described above (1:1000 dilution).

##### Detection of FLAG-Cereblon and Myc-14-3-3ζ Variants from Co-immunoprecipitation

Samples were loaded onto SDS-PAGE gels and transferred to membranes similarly as described above. Membranes were probed with either Anti-Myc Antibody (Cell Signaling Technology, 2272S) (1:1000 dilution) or Anti-FLAG M2 Antibody (1:1000 dilution). Membranes were washed and then incubated with either IRDYE 680RD Goat Anti-Rabbit Secondary Antibody or IRDYE 680RD Goat Anti-Mouse Secondary Antibody.

### HBplus Analysis of pSer-Containing Structures

Structures determined at <2.5 Å resolution containing phosphoserine (residue ID: SEP) within a peptide chain were pulled from the Protein Data Bank (971 entries). The PISCES server^81^ was used to filter PDBs with <75% sequence identity, producing a list with 171 unique structures containing pSer. The PDB files for these structures were downloaded and HBplus^66^ was used to assess hydrogen bonds for pSer residues in each structure using default parameters, except that the maximum donor to acceptor (D–A) distance was set to 3.5 Å.

## Supporting Information Available

Detailed methods and Supporting Figures and Tables containing: SEC-MALS statistics, SDS-PAGE and Phos-tag gels of phosphoserine containing proteins, efficiency of nhpSer incorporation into sfGFP, generation of T7 promoter variants, MS/MS analyses, pSer and nhpSer incorporation into a HSPB6 peptide, ^31^P NMR spectra, mechanism of GSK3β activation, GSK3β phosphorylation of full-length SARS-CoV-2 nucleocapsid protein, SEC-MALS and equilibrium AUC analyses of 14-3-3/HSPB6 complexes, SDS-PAGE and Phos-tag analyses of pSer/nhpSer containing HSPB6 proteins post AUC analyses, R18 peptide binding to wild-type and phosphorylated monomeric 14-3-3ζ, stability of 4-3-3ζ pSer58/nhpSer58 proteins in cell lysates, volcano plots comparing wild-type, pSer58 and nhpSer58 14-3-3ζ interactomes, and contributions of the γ-oxygen of pSer to protein–protein interactions in phospho-serine binding protein families. Also included are protein sequences used in this study (PDF)
Supporting Data Set 1: Processed proteomics data sets with enrichment scores and p-values for all identified proteins (XLSX)
Supporting Data Set 2: HBplus analysis of the Protein Data Bank evaluating the contribution of pSer γ-oxygens to protein–protein interactions (XLSX)
Raw peptide intensities for proteomics data sets (XLSX)
