# Supplementary material for: Autonomous Synthesis of Functional, Permanently Phosphorylated Proteins for Defining the Interactome of Monomeric 14-3-3ζ
Source: ACS Cent Sci. 2023 Apr 10;9(4):816–35. doi: 10.1021/acscentsci.3c00191 (PMC10141581; doi:10.1021/acscentsci.3c00191)
Supplement: Supplementary file 1 — oc3c00191_si_001.pdf [file oc3c00191_si_001.pdf]

SUPPORTING INFORMATION

Autonomous synthesis of functional, permanently phosphorylated proteins for defining the interactome of monomeric 14-3-3ζ

Phillip Zhu[1], Stanislau Stanisheuski[2], Rachel Franklin[1], Amber Vogel[1], Cat Hoang Vesely[1], Patrick Reardon[1], Nikolai N. Sluchanko,[4] Joseph S. Beckman[1,3], P. Andrew Karplus[1], Ryan A. Mehl[1], and Richard B. Cooley[1*]

[1] Oregon State University, Department of Biochemistry and Biophysics, 2011 Agricultural and Life Sciences, Corvallis, OR 97331 USA

[2] Oregon State University, Department of Chemistry, 153 Gilbert Hall, Oregon State University, Corvallis, Oregon 97331 USA

[3] e-MSion Inc., 2121 NE Jack London St, Corvallis, Oregon 97330 USA

[4] A.N. Bach Institute of Biochemistry, Federal Research Center of Biotechnology of the Russian Academy of Sciences, 119071, Moscow, Russia

* To whom correspondence should be addressed: Oregon State University, Department of Biochemistry and Biophysics, 2011 Agricultural and Life Sciences, Corvallis, OR 97331

Telephone: (541) 737-4870 Fax: (541) 737-0481 Email: rick.cooley@oregonstate.edu

---

Supporting Methods……………………………………………………….……………………p. 2

Supporting Tables……………………………………………………….……………………p. 11

Supporting Figures……………………………………………………………………...p. 13

Supporting Protein Sequences…………………………………………………………p. 45

Primer Table………………………………………………………………………………p. 48

References…………………………………………………………………………………..p. 50

SUPPORTING METHODS

Generating the Frb-v1 nhpSer biosynthetic pathway.

Assembly of T7 promoter mutant library. The sequence of six nucleotides within the T7 promoter known to control transcriptional efficiency[1] were mutated to all combinations (4^6 = 4096 mutants) and then cloned in front of a sfGFP reporter gene in the pET28 plasmid backbone. This was done by amplifying DNA fragments off the pET28-sfGFP plasmid (Addgene #85492) using primer pairs P1/P2 and P3/P4 (see Primer Table below) to create two overlapping fragments that were then recombined using SLiCE[2] into pET28-sfGFP digested with BglII and NcoI. The assembled library of promoter mutants was electroporated into DH10b cells, which were recovered for 1 hr in SOC media and then transferred to a liquid culture of 2xYT media (16 g/L tryptone, 10 g/L yeast extract, 5 g/L NaCl) containing 50 μg/mL kanamycin to grow transformants overnight at 37 °C. A small portion of the recovered cells was also plated on LB/agar with kanamycin, and counted the next day revealing 5 x 10^4 transformants were obtained during library assembly and transformation for complete library coverage. The resulting pET28-T7lib-sfGFP library was miniprepped from cells grown in overnight liquid culture.

Selection of T7 promoter mutants. The pET28-T7lib-sfGFP library was transformed into BL21(DE3) Δ*serC* cells by electroporation. After 1 hr of recovering in SOC medium, cells were plated on LB/agar plates containing 1 mM IPTG, and grown for 24 hrs at which time 94 colonies displaying various levels of fluorescence were used to inoculate 0.5 mL of ZY-non-inducing media (ZY-NIM)[3]. Cells were grown at 37 °C with shaking at 300 rpm overnight, at which point 20 μL of each culture was used to inoculate 0.5 mL of ZY-auto-inducing media (ZY-AIM).[3] After 24 hrs of growth at 37 °C, end-point in-cell sfGFP fluorescence was measured with a microplate reader. Twenty-five variants displaying a fluorescence range spanning two-orders of magnitude were selected for DNA isolation by miniprep. Isolated DNA was re-transformed into DH10b cells. To confirm expression reproducibility at larger scales, isolated variants were again transformed back into BL21(DE3) Δ*serC* cells and expressed in duplicate at the 50 mL scale in ZY-AIM media. Variants that did not reproduce the same sfGFP expression level as observed in the first round

were discarded. After a third round of expression at 50 mL scale, T7 promoter variants that displayed reproducible sfGFP expression levels at high (34%), medium (11%), low (4%) and trace (1%) levels, with wild-type designated as 100%, were sequenced and selected for downstream Frb pathway assembly.

FrbABCDE library assembly strategy. The FrbABCDE library assembly strategy was adapted from work by Zhao and colleagues[1] such that all permutations of transcriptional promoters in front of each of the five Frb genes were made via Golden Gate assembly (5^5 = 3125 combinations). Each gene was flanked by its own promoter and terminator so that each is transcribed independently. The plasmid backbone contained a CDF origin of replication and spectinomycin resistance to ensure compatibility and stable propagation with the nhpSer GCE machinery (pUC origin/kanamycin resistance) and target protein expression plasmids (p15a origin/ampicillin resistance). Since it has not yet been established whether FrbB or FrbE, or both, is responsible for the isocitrate dehydrogenase-like activity of the fourth step of the pathway, both were included in the library assembly.

Library assembly. Each of the five Frb genes from *Streptomyces rubellomurinus* were codon optimized for *E. coli*, synthesized, subcloned into NcoI/XhoI digested pET28 using SLiCE, and sequence verified. Sequences of each Frb protein are included below. Each Frb gene was then PCR amplified using primers that flanked the T7 promoter and terminator (see Primer Table below). In each PCR reaction the forward primer was a mixture of five that encoded each of the four transcriptional mutants identified above plus wild-type, and a unique Type IIS BsaI restriction site. The reverse primer for each fragment also contained a unique BsaI restriction site. The CDF origin and spectinomycin resistance gene from pCDFduet (Novagen) were also amplified as a single fragment with flanking BsaI restriction sites. All BsaI overhangs were designed so that each of the five *promoter-FrbX-terminator* fragments and the CDF-Spec^R backbone could be ligated directionally into a single circular plasmid. Forward primer mix P7-P11 and reverse primer P12 were used to amplify FrbA, P13-P17 and P18 for FrbB, P19-P23 and P24 for FrbC, P25-P29 and P30 for FrbD, P31-P35 and P36 for FrbE, and lastly P5 and P6 for CDF-Spec^R . To assemble these 6 fragments into a circular plasmid, approximately 620, 1100 and 590 ng of gel-purified CDF-Spec^R (3200bp), FrbA

(2900bp) and FrbB (1355bp) DNA fragments, respectively, were mixed with 20 U of BsaI-HF v2 and 2000 U of T4 ligase (NEB) in 1x ligase buffer in a total of 25 μL total volume. Similarly, 610, 500 and 610 ng of FrbC (1418bp), FrbD (1127bp) and FrbE (1394bp), respectively, were mixed with the same amounts of BsaI and T4 ligase in a separate tube. The two reaction mixtures were then incubated in a PCR thermocycler using the following protocol: Step 1: 37 ^{o}C for 5 min, Step 2: 16 ^{o}C for 5 min, repeat 15 times. Then, the two mixtures were combined and the reaction continued as follows: Step 1: 37 ^{o}C for 5 min, Step 2: 16 ^{o}C for 5 min, repeat 15 times; Step 3: 80 ^{o}C 20 min, Step 4: 12 ^{o}C 5 min. The ligation mixture was directly transformed into DH10b cells by electroporation, recovered in SOC for 1 hr and then grown in 2xYT containing spectinomycin to select for transformants. A small portion of the recovered cells was plated on LB/agar with spectinomycin, and counted the next day revealing 390,000 transformants were obtained for approximately 125-fold coverage of the library. DNA from cells grown overnight was miniprepped yielding the pCDF-FrbABCDE_lib DNA. Plasmid from 10 individual clones was miniprepped and digested with NcoI and NheI. Of these, 8 showed the predicted DNA fragment pattern following digestion demonstrating faithful assembly of the targeted library.

Screening for functional FrbABCDE assemblies. The pCDF-FrbABCDE_lib was screened for assemblies able to synthesize nhpSer for translational incorporation into sfGFP-150TAG. Approximately 0.5 μg of pCDF-FrbABCDE_lib was electroporated with the nhpSer GCE machinery plasmid, pSF-nhpSer (see below), and the reporter expression plasmid, pRBC-sfGFP-150TAG, into BL21(DE3) Δ*serC* cells. The Δ*serC* mutation ensures the expression host cannot biosynthesize pSer, eliminating the chances that pSer could get incorporated into sfGFP by the GCE machinery. Thus, in this system cells will fluoresce only if nhpSer is biosynthesized by the FrbABCDE pathway and then incorporated at the 150TAG codon of sfGFP. This was confirmed with a negative control transformation in which pCDF-FrbABCDE_lib was replaced with an "empty" pCDFduet vector. After transformation, cells were recovered for 1 hr in SOC media at 37 ^{o}C and plated onto LB/agar plates containing 0.5% glycerol, 1mM IPTG, 50 μg/mL ampicillin, 25 μg/mL kanamycin and 50 μg/ml spectinomycin. Plates were incubated at 37 ^{o}C for 18 hrs, and then another 18 hrs

at room temperature. No fluorescence was observed for colonies containing the "empty" pCDFduet vector. From cells transformed with pCDF-frbABCDE_lib, 92 fluorescent clones were selected to inoculate 0.5 mL ZY-NIM, which were grown for 16 hrs at 37 ºC shaking at 300 rpm. Next, these cells were used to inoculated 0.5 mL 2xYT + 0.5% glycerol, which were grown at 37 ºC for 3 hrs at which time expression was induced with 1 mM IPTG and temperature lowered to 30 ºC. After 24 hr, in-cell fluorescence was measured and the top 6 clones were selected for follow up analysis. Plasmid DNA was isolated from these top 6 clones, and the pCDF-FrbABCDE pathway plasmids were isolated by transformation into DH10b cells, plating on LB/agar with spectinomycin only and selecting single colonies for subsequent plasmid isolation. T7 promoter identities for these six isolates were determined by sequencing. Pathway functionality of the six pathways were re-tested in subsequent expressions at 50 mL scale, in triplicate.

General protocol for expression of nhpSer containing proteins

Overview of plasmid architecture. nhpSer incorporation using the Frb-v1 pathway requires three plasmids. (1) pRBC/pRBCduet: expresses the TAG-codon interrupted target gene of interest with expression driven by the T7 promoter. Contains p15a origin of replication conferring ampicillin resistance.[3] (2) pSF-nhpSer or pERM-nhpSer. The pSF-nhpSer GCE machinery plasmid expresses the SepRS-2[4] under a constitutive GlnRS promoter with the native GlnS terminator, the Sep-tRNA$^{v2.0}_{CUA}$ under a constitutive lpp promoter (no terminator),[5] the EF-Sep under the control of a lac promoter (no terminator),[6] and *E. coli serB* under an oxb18 constitutive recA promoter. The Sep-tRNA$^{v2.0}_{CUA}$ was used here for its enhanced orthogonality.[5] Constitutive expression of *serB* is used to enhance hydrolysis of intracellular pSer amino acid. Contains pUC origin of replication conferring kanamycin resistance. A modified form of this plasmid (pERM-nhpSer, Kan resistance) was built with additional levels of transcriptional insulation to ensure independent expression of each component: SepRS-2[4] expression was controlled by a constitutive GlnRS promoter and a rrnB T1 transcriptional terminator, the Sep-tRNA$^{v2.0}_{CUA}$ [5] is controlled by a constitutive lpp promoter and rrnC terminator, the EF-Sep[6] is controlled of a lac promoter and T7 terminator, and the *E. coli serB* was

expressed under a stronger constitutive oxb20 promoter and rrnG terminator. (3) pCDF-FrbABCDE Frb-v1 plasmid: expresses all five Frb enzymes. Each gene is flanked by its own T7 promoter variant and T7 terminator as indicated in main text. Contains CloDF origin of replication conferring spectinomycin resistance. BL21(DE3) Δ*serC* cells were used for all nhpSer protein expressions.

General protocol for expression of nhpSer containing proteins with Frb-v1. Fresh transformations were performed for every expression. The pCDF-FrbABCDE Frb-v1.0 pathway, pSF-nhpSer (or pERM-nhpSer) and appropriate pRBC plasmids were co-transformed simultaneously by electroporation into BL21(DE3) Δ*serC* cells. The cells were recovered for 60-90 min in SOC media at 37 °C, and plated onto LB/agar plates containing 50 μg/mL ampicillin, 25 μg/mL kanamycin and 50 μg/ml spectinomycin. After overnight growth at 37 °C, cells were scraped and used to inoculate 2xYT media supplemented with 0.5% glycerol and the same antibiotics. These cultures were grown for 3-5 hours at 37 °C, at which point they were used to inoculate Terrific Broth (TB) expression cultures (TB: 12 g/L tryptone, 24 g/L yeast extract, 0.5% glycerol, 72 mM K_2HPO_4, 17 mM KH_2PO_4). Baffled flasks were used to ensure high rates of aeration, and Antifoam B (Sigma #A5757) was used to limit foam buildup. After inoculation, TB expression cultures were grown at 37 °C until OD_{600} reached ~1.0, then 0.5 mM IPTG was added and the temperature was adjusted to 20-30 °C depending on the target protein. Cultures were harvested 20-24 hrs after the addition of IPTG.

Expression of nhpSer containing proteins by media supplementation. BL21(DE3) Δ*serC* cells were co-transformed with pRBC-sfGFP-150TAG or pRBC-sfGFP-134/150TAG and pSF-nhpSer, and grown overnight LB/agar with appropriate antibiotics. After overnight growth, a starter culture of 2xYT was inoculated with several colonies and grown at 37 °C for 3-4 hrs. 1 mL of this starter culture was used to inoculate a fresh 50 mL 2xYT culture, which was grown to an OD ~0.6-0.8 at 37 °C, and then expression was induced with the addition of 1 mM IPTG and 2 mM nhpSer (DL-AP4, Abcam). Cells were harvested after 18 hrs of expression at 37 °C.

General protocol for expression of pSer containing proteins.

Overview of plasmid architecture. pSer incorporation requires two plasmids. (1) pRBC/pRBCduet: as described above. (2) pKW2-EFSep: expresses the SepRS-2 under a constitutive GlnRS promoter, the Sep-$tRNA_{CUA}$ G4 under a constitutive lpp promoter, and the EF-Sep under the control of a lac promoter.[4] Contains a pBR322 origin of replication and confers chloramphenicol resistance. Proteins were expressed in either BL21(DE3) Δ*serB* or B95(DE3) Δ*A* Δ*fabR* Δ*serB*.[3] The latter, which is a partially recoded derivative of BL21(DE3),[7] lacks Release Factor-1 and therefore was used to avoid truncated protein.[3]

General protocol for expression of pSer containing proteins. Fresh transformations were performed for every expression. Proteins were expressed exactly as described previously.[3] Briefly, BL21(DE3) Δ*serB* or B95(DE3) Δ*A* Δ*fabR* Δ*serB* cells were co-transformed with the appropriate pRBC/pRBCduet vector and the pKW2-EFSep vector, grown overnight on LB/agar plates with ampicillin and chloramphenicol, and then multiple colonies were used to inoculate an overnight ZY-non inducing media culture. After overnight growth at 37 ^{o}C, these non-inducing cells were used to inoculate a ZY-auto inducing culture, which was grown to an OD ~1.0 at 37 ^{o}C, at which point the temperature was adjusted as needed for target protein expression. Cells were harvested ~18-24 hrs later after temperature adjustment.

Expression and purification details of specific target proteins.

sfGFP: The pRBC-sfGFP wt, 150TAG and 134/150TAG plasmids containing C-terminal His_6 affinity purification tags were as previously described.[3] pSer and nhpSer containing proteins were expressed as described above at 37 ^{o}C. Cells were lysed in Wash Buffer (50 mM Tris, 500 mM NaCl, 5% glycerol, 5 mM imidazole pH 7.4) containing a phosphatase inhibitor mixture (50 mM NaF, 5 mM sodium pyrophosphate and 0.5 mM sodium orthovanadate). Protein was bound to TALON metal affinity resin, washed with 20-50 column volumes of Wash Buffer, and eluted with Wash Buffer + 300 mM imidazole. Proteins were desalted using a PD-10 column (GE Healthcare) into 50 mM Tris pH 7.4, 150 mM NaCl, frozen in liquid N_2 and stored at -80 ^{o}C.

bdSUMO-HSPB6$^{11\text{-}20}$ peptide fusion protein: A codon optimized HSPB6 gene fragment (residues 11-20) containing either a codon for Ser or a TAG codon at S16 were cloned into pRBC with an N-terminal flanking *bd*SUMO fusion tag (residues 19-97)[8] and a C-terminal, TEV cleavable flanking sfGFP-His$_6$ fusion tag. Wildtype, pSer and nhpSer variants were expressed as described above at 30 ^{o}C. *bd*SUMO-HSPB6$^{11\text{-}20}$-sfGFP-His$_6$ proteins were purified as described above for sfGFP, eluted with 300 mM imidazole and desalted into 25 mM Tris pH 7.4, 25 mM NaCl. Desalted proteins were then treated with His$_6$ tagged TEV protease overnight at 4 ^{o}C at a 1:100 molar ratio protease:substrate and the *bd*SUMO-HSPB6$^{11\text{-}20}$ protein was isolated by reverse affinity. Proteins were concentrated to 55 mg/mL (~5 mM), flash frozen in liquid N_2 and stored at -80 ^{o}C. Total *bd*SUMO-HSPB6$^{11\text{-}20}$-sfGFP-His$_6$ yields after purification were ~ 100 mg/L culture for pSer and ~70 mg/L culture for nhpSer containing proteins.

14-3-3ζ / HSPB6 complexes. The genes for human 14-3-3ζ (residues 2-229) and HSPB6 (2-153) were codon optimized, synthesized and cloned into orf1 and orf2, respectively, of pRBCduet. HSPB6 was fused at its N-terminus with a His$_6$-*bd*SUMO tag while the 14-3-3ζ protein had no purification or solubility tag. Mutations S16D and S16TAG were introduced into HSPB6 using SLiCE. Expressions were performed as described above, but at 25 ^{o}C, and purified similarly as sfGFP, except that protein complexes were eluted by proteolytic on-column cleavage with 30 nM *bd*SENP1 for 1 hr at 4 ^{o}C.[9] Proteins were gel-filtered on a Superdex-75 column (GE Healthcare) in 25 mM Tris and 150mM NaCl pH 7.4, flash frozen with liquid N_2 and stored at -80 ^{o}C. Total protein yields after purification were ~ 10 mg/ L culture for the pSer dependent complex and ~ 8 mg/ L culture for the nhpSer dependent complex.

14-3-3ζ variants: The same codon optimized 14-3-3ζ gene (residues 2-229) used for generating complexes with HSPB6 was cloned into pRBC with an N-terminal His$_6$-*bd*SUMO fusion tag. Mutations S58E and S58TAG were introduced using SLiCE. Proteins were expressed at 25 ^{o}C, purified as done for the 14-3-3ζ / HSPB6 complexes, eluted using the same *bd*SENP1 on-column cleavage method, and then gel-filtered on a Superdex S-75 column in 25 mM Tris and 150 mM NaCl pH 7.4. Total protein yields after purification were ~ 12 mg/L culture for the pSer variant and ~ 8 mg/L culture for the nhpSer variant.

SARS-CoV-2 Nucleocapsid protein: Linker Np-sfGFP fusion proteins: A codon optimized SARS-CoV-2 Nucleocapsid Protein gene corresponding to residues 175-245 containing either a codon for Ser or a TAG codon at either S188 or S206 were cloned into pRBC with an N-terminal flanking *bd*SUMO fusion tag (residues 19-97) and a C-terminal, TEV cleavable flanking sfGFP-His_6 fusion tag. Proteins were expressed at 25 ^oC and purified similarly, except that untagged 0.5 μM *bd*SENP1 was added to the lysate while proteins bound to Ni-NTA resin for 1 hr to cleave off the *bd*SUMO tag. After sufficient washing to remove the cleaved *bd*SUMO and *bd*SENP1, Linker Np-sfGFP-His_6 protein was eluted with 300 mM imidazole and desalted into 50 mM Tris pH 7.4, 350 mM NaCl prior to GSK3β reactions. pSer variants at both sites yielded ~ 90 mg/L culture and nhpSer variants at both sites yielded ~ 70 mg/L culture. For mass spectrometry analysis, Linker Np-sfGFP-His_6 proteins were proteolyzed by TEV (1:100 ratio) overnight at 4 ^oC, and the Linker Np peptides were isolated by reverse affinity chromatography.

SARS-CoV-2 Nucleocapsid protein: Full-length: A codon optimized SARS-CoV-2 nucleocapsid protein (residues 2-420) gene containing either a codon for Ser or a TAG codon at either S188 or S206 were cloned into pRBC containing an N-terminal His6-*bd*SUMO using SLiCE. Protein variants were expressed as described above at 25 ^oC, and then purified by Ni-NTA affinity chromatography. Prior to elution, the resins were washed with 50 bed volumes of High Salt Wash Buffer (50 mM Tris pH 7.4, 3 M NaCl, 20 mM imidazole, 50 mM NaF, 5 mM sodium pyrophosphate and 0.5 mM sodium orthovanadate) to remove bound nucleic acids.[10] Proteins were then eluted by proteolysis from Ni-NTA using *bd*SENP1 in 50 mM Tris pH 7.4, 150 mM NaCl, 20 mM imidazole, 50 mM NaF, 5 mM sodium pyrophosphate and 0.5 mM sodium orthovanadate. Dimeric proteins were purified by gel-filtration in 50 mM Tris pH 7.4, 150 mM NaCl and concentrated to 100 μM in preparation for GSK3β reactions. Concentrated proteins were flash frozen in liquid N_2 and stored at -80 °C until use. pSer variants at both sites yielded ~ 12 mg/L culture and nhpSer variants at both sites yielded ~ 7 mg/L culture.

GSK3β: A codon optimized gene containing GSK3β (residues 2-420) containing the S9A mutation (to prevent auto-inhibition) was cloned into a pET28 vector harboring an N-terminal His_6-*bd*SUMO using

SLiCE. Proteins were expressed in *E. coli* BL21(DE3) in TB medium supplemented with 50 μg/mL Kanamycin; expression of the GSK3β fusion protein was induced by 0.3 mM IPTG at OD 0.6-0.8 and continued at 18 °C for 16 h. Protein was purified by Ni-NTA and eluted with *bd*SENP1 cleavage in 50 mM Tris pH 7.4, 150 mM NaCl, 10% glycerol. Protein was frozen in liquid N_2 and stored at -80 ºC until use.

FLAG- or Myc-14-3-3ζ variants: The FLAG- or Myc- tags were cloned into the region between the C-terminal cleavage site of bdSUMO (GG), and the N-terminus Met of 14-3-3ζ in our original expression constructs for the 14-3-3ζ variants using SLiCE. Proteins were expressed and purified similarly to the 14-3-3ζ variants described above.

SUPPORTING TABLES

Table S1. SEC-MALS statistics for 14-3-3ζ ΔC/HSPB6 complexes shown in Main Text Figure 5C. Theoretical masses: 14-3-3ζ ΔC dimer = 52.4 kDa, HSPB6 dimer = 33.0 kDa, 14-3-3ζ ΔC dimer + HSPB6 dimer complex = 85.4 kDa.

	Mw (kDa)	Uncertainty	Calculated mass (µg)	Mass fraction (%)
WT_1433_run1	51.7	0.20%	106.76	100
WT_1433_run2	51.5	0.30%	96.33	100
Average	51.6		101.55	100
Standard deviation	0.1		7.38	0
% Standard deviation	0.2		7.26	0
Minimum	51.5		96.33	100
Maximum	51.7		106.76	100

	Mw (kDa)	Uncertainty	Calculated mass (µg)	Mass fraction (%)
HSPB6 WT run 1	34.4	0.30%	108.17	100
HSPB6 WT run 2	34.5	0.30%	98.02	100
Average	34.4		103.1	100
Standard deviation	0.1		7.18	0
% Standard deviation	0.3		6.96	0
Minimum	34.4		98.02	100
Maximum	34.5		108.17	100

	Mw (kDa)	Uncertainty	Calculated mass (µg)	Mass fraction (%)
pSer Complex run 1	79.1	0.90%	393.4	100
pSer Complex run 2	78.9	0.90%	358.77	100
Average	79		376.09	100
Standard deviation	0.1		24.49	0
% Standard deviation	0.2		6.51	0
Minimum	78.9		358.77	100
Maximum	79.1		393.4	100

	Mw (kDa)	Uncertainty	Calculated mass (µg)	Mass fraction (%)
nhpSer Complex run 1	80.1	1.00%	389.63	100
nhpSer Complex run 2	80.2	1.00%	384.83	100
Average	80.1		387.23	100
Standard deviation	0		3.4	0
% Standard deviation	0.1		0.88	0
Minimum	80.1		384.83	100
Maximum	80.2		389.63	100

Table S2. SEC-MALS statistics for 14-3-3ζ S58 variants showin in Main Text Figure 6C. Theoretical masses: 14-3-3ζ ΔC dimer = 52.4 kDa, 14-3-3ζ ΔC monomer = 26.2 kDa

	Mw (kDa)	Uncertainty	Calculated mass (µg)	Mass fraction (%)
WT_run1	51.4	1.30%	88.82	96.2
WT_run2	51.1	1.40%	83.89	95.9
Average	51.2		86.36	96
Standard deviation	0.3		3.49	0.2
% Standard deviation	0.5		4.04	0.2
Minimum	51.1		83.89	95.9
Maximum	51.4		88.82	96.2

	Mw (kDa)	Uncertainty	Calculated mass (µg)	Mass fraction (%)
S58E_run1	50.4	0.20%	95.61	100
S58E_run2	50	0.30%	94.32	100
Average	50.2		94.96	100
Standard deviation	0.3		0.92	0
% Standard deviation	0.5		0.97	0
Minimum	50		94.32	100
Maximum	50.4		95.61	100

	Mw (kDa)	Uncertainty	Calculated mass (µg)	Mass fraction (%)
pSer58_run1	25.3	2.00%	85.76	100
pSer58_run2	25.9	2.30%	106.09	100
Average	25.6		95.92	100
Standard deviation	0.5		14.38	0
% Standard deviation	1.8		14.99	0
Minimum	25.3		85.76	100
Maximum	25.9		106.09	100

	Mw (kDa)	Uncertainty	Calculated mass (µg)	Mass fraction (%)
nhpSer58_run1	26.5	1.40%	107.23	100
nhpSer58_run2	26.6	1.00%	101.02	100
Average	26.5		104.13	100
Standard deviation	0		4.39	0
% Standard deviation	0.1		4.22	0
Minimum	26.5		101.02	100
Maximum	26.6		107.23	100

SUPPORTING FIGURES

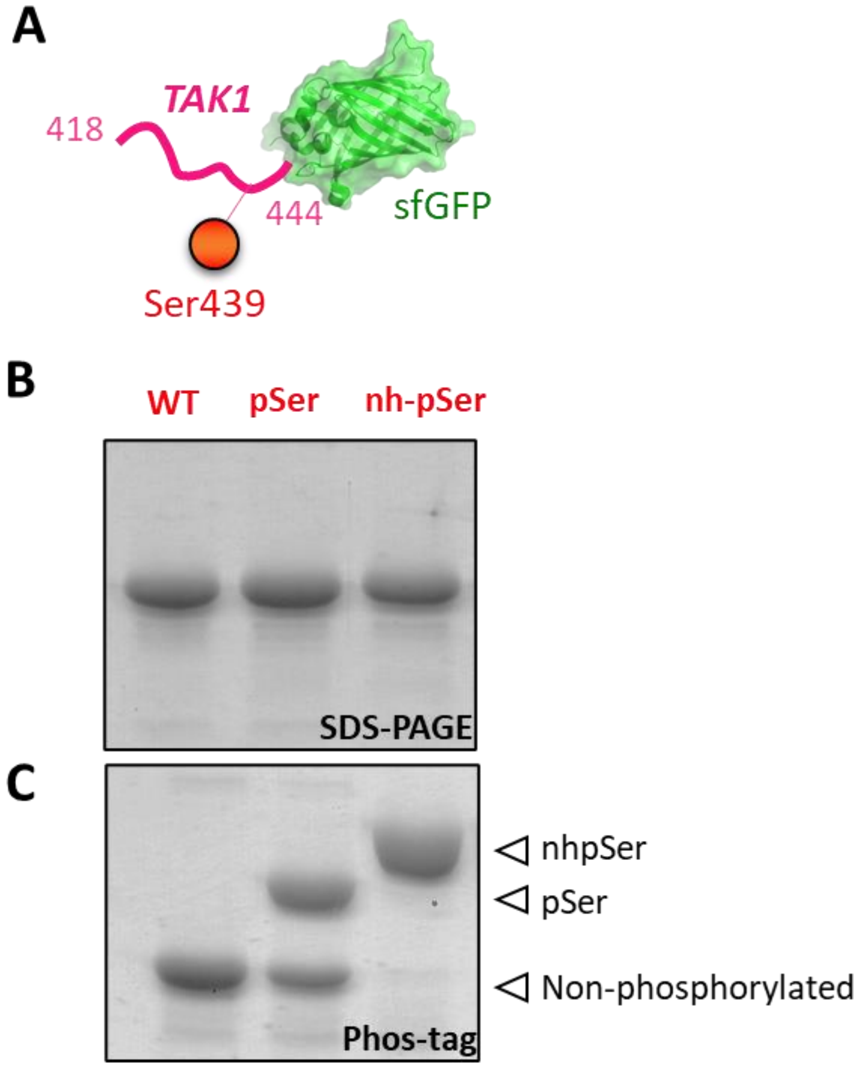

Figure S1. Phosphoserine containing proteins can be hydrolyzed during expression in *E. coli*. (A) Construct of human TAK1 (also known as Mitogen-activated protein kinase kinase kinase 7; Uniprot no. O43318) with residues 418-444 fused to sfGFP. Ser439 of TAK1 is a well-established site of phosphorylation.[11] (B) SDS-PAGE gel of TAK1$^{418\text{-}444}$—sfGFP with Ser (WT), pSer and nhpSer incorporated at site S439 via GCE. pSer incorporation was achieved as previously described by Zhu et al.[3], while nhpSer incorporation was achieved using PermaPhos Frb-v1 as described in this manuscript. (C) Phos-tag gel of the same proteins shown in panel B. In Phos-tag gel electrophoresis, phosphorylated proteins migrate slower than the non-phosphorylated counter-parts.[12] NhpSer-containing proteins migrate slower than the equivalent pSer-containing proteins.[13] Phos-tag electrophoresis of the pSer-incorporated protein shows ~50% of the protein was hydrolyzed during expression/purification.

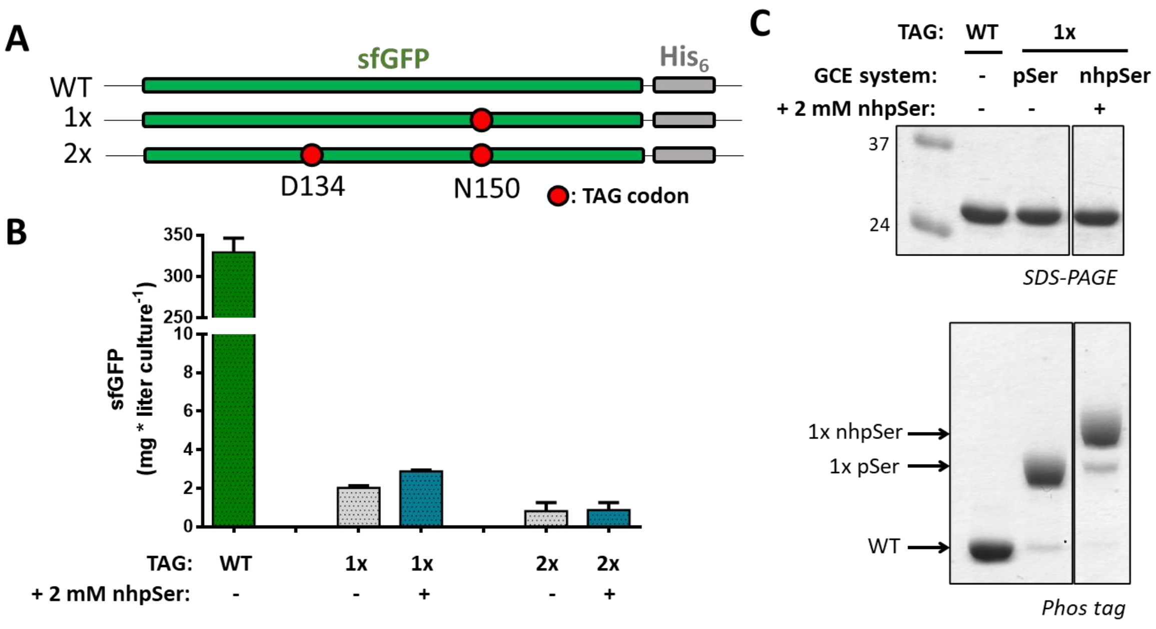

Figure S2. Efficiency of nhpSer incorporation into sfGFP using the previously published GCE system.[4] (A) sfGFP fluorescent reporter constructs containing one (1x, 150TAG) and two (2x, 134/150TAG) amber stop codons. (B) sfGFP expression yields based on in-cell fluorescence measurements using the current nhpSer GCE in which nhpSer is supplemented to the media at 2 mM final concentration. Expressions were performed in BL21(DE3) Δ*serC* cells using 2xYT media and 1 mM IPTG to induce expression. Cells were cultured for 18 hrs at 37 ^{o}C prior to fluorescence measurements. Yield of sfGFP per liter culture was calculated by subtracting the contribution of auto-fluorescence from cells not expressing any sfGFP construct from the fluorescence of the indicated cultures, and then fluorescence values were converted to sfGFP concentration based on a standard curve of purified sfGFP. Error bars represent standard deviations of expressions performed in triplicate. (C) SDS-PAGE and Phos tag gels of purified sfGFP wild-type, pSer150 and nhpSer150 (produced via media supplementation). The slower electrophoretic mobility of sfGFP-nhpSer150 compared to sfGFP-pSer150 is consistent with previous reports.[13] The same data are shown in the main text Fig. 3B.

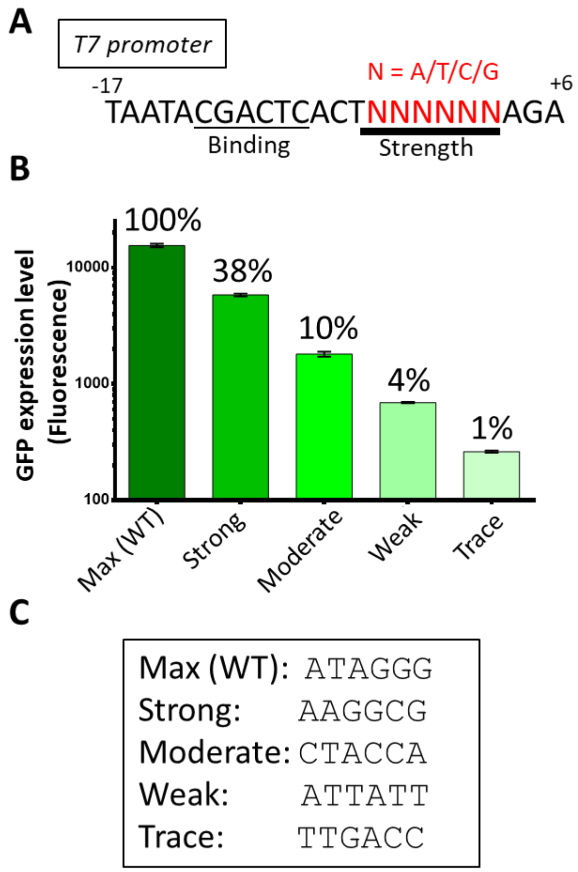

Figure S3. Generating T7 promoter variants with attenuated transcriptional strengths. (A) Sequence of the T7 transcriptional promoter highlighting the region regulating transcription initiation strength.[1] Nucleotides highlighted in red were randomized to all four nucleotides and placed in front of an sfGFP reporter gene. (B) After screening the library for promoter variants conferring attenuated sfGFP expression, four were identified displaying end point yields spanning two orders of magnitude. Error bars represent standard deviation from three replicate cultures. (C) Sequences of the randomized region (red, panel A) for five T7 promoters used in this study to generate the FrbABCDE library.

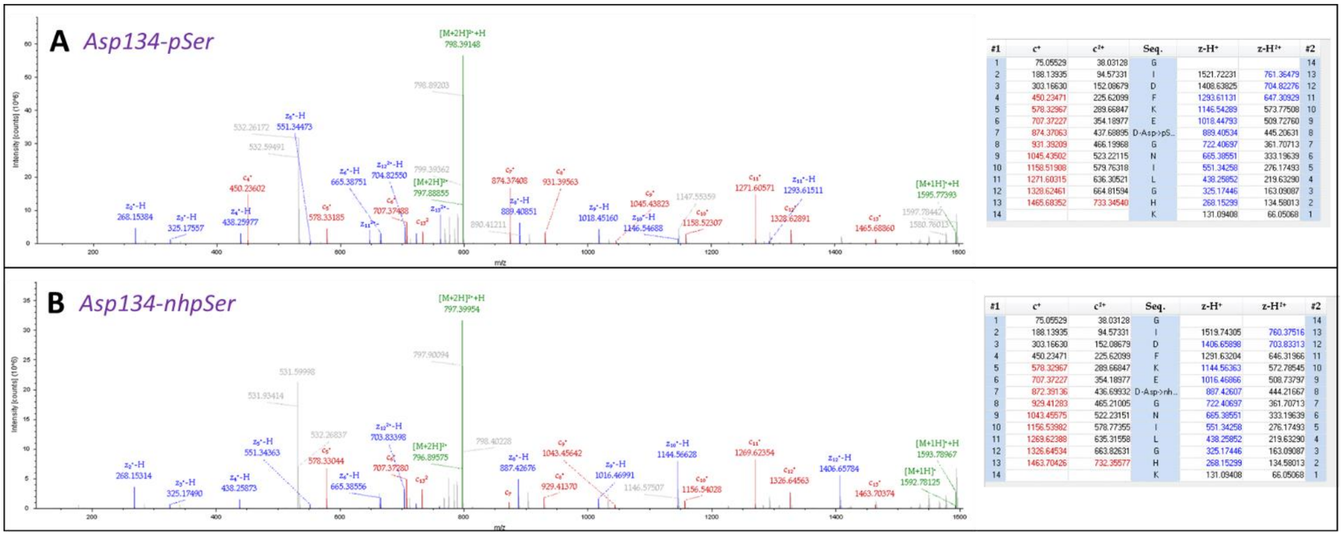

#1	c⁺	c²⁺	Seq.	z-H⁺	z-H²⁺	#2
1	75.05529	38.03128	G			14
2	188.13935	94.57331	I	1521.72231	761.36479	13
3	303.16630	152.08679	D	1408.63825	704.82276	12
4	450.23471	225.62099	F	1293.61131	647.30929	11
5	578.32967	289.66847	K	1146.54289	573.77508	10
6	707.37227	354.18977	E	1018.44793	509.72760	9
7	874.37063	437.68895	D-Asp->pS...	889.40534	445.20631	8
8	931.39209	466.19968	G	722.40697	361.70713	7
9	1045.43502	523.22115	N	665.38551	333.19639	6
10	1158.51908	579.76318	I	551.34258	276.17493	5
11	1271.60315	636.30521	L	438.25852	219.63290	4
12	1328.62461	664.81594	G	325.17446	163.09087	3
13	1465.68352	733.34540	H	268.15299	134.58013	2
14			K	131.09408	66.05068	1

#1	c⁺	c²⁺	Seq.	z-H⁺	z-H²⁺	#2
1	75.05529	38.03128	G			14
2	188.13935	94.57331	I	1519.74305	760.37516	13
3	303.16630	152.08679	D	1406.65898	703.83313	12
4	450.23471	225.62099	F	1291.63204	646.31966	11
5	578.32967	289.66847	K	1144.56363	572.78545	10
6	707.37227	354.18977	E	1016.46866	508.73797	9
7	872.39136	436.69932	D-Asp->nh...	887.42607	444.21667	8
8	929.41283	465.21005	G	722.40697	361.70713	7
9	1043.45575	522.23151	N	665.38551	333.19639	6
10	1156.53982	578.77355	I	551.34258	276.17493	5
11	1269.62388	635.31558	L	438.25852	219.63290	4
12	1326.64534	663.82631	G	325.17446	163.09087	3
13	1463.70426	732.35577	H	268.15299	134.58013	2
14			K	131.09408	66.05068	1

Figure S4. Bottom-up MS/MS fragmentation of trypsin digested peptides confirming incorporation of (A) pSer at site D134 of 2x-TAG sfGFP made using the pSer GCE system and (B) nhpSer at site D134 of the 2x-TAG sfGFP using the nhpSer Frb-v1 GCE system.

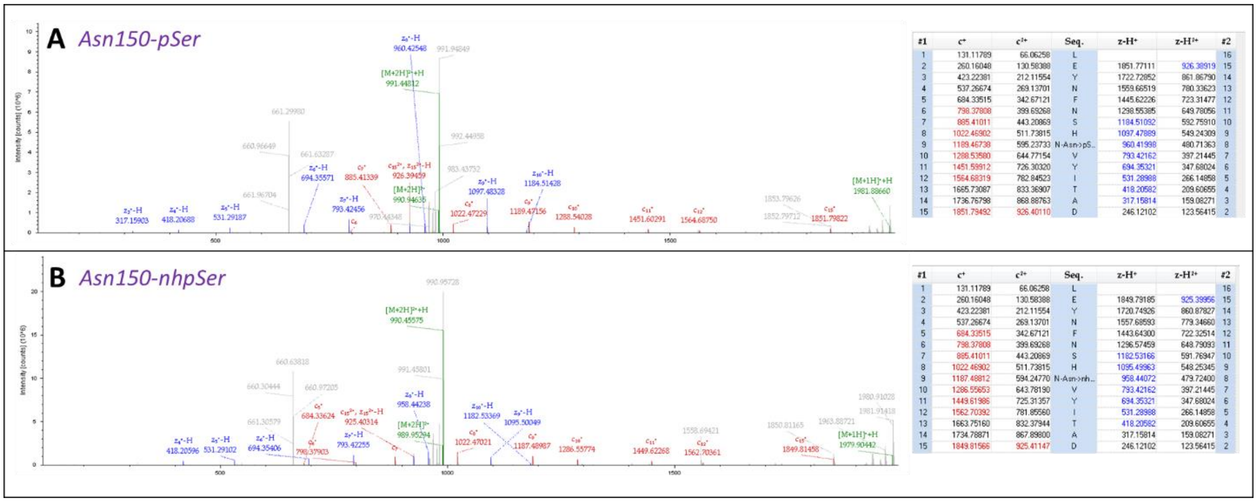

#1	c⁺	c²⁺	Seq.	z-H⁺	z-H²⁺	#2
1	131.11789	66.06258	L			16
2	260.16048	130.58388	E	1851.77111	926.38919	15
3	423.22381	212.11554	Y	1722.72852	861.86790	14
4	537.26674	269.13701	N	1559.66519	780.33623	13
5	684.33515	342.67121	F	1445.62226	723.31477	12
6	798.37808	399.69268	N	1298.55385	649.78056	11
7	885.41011	443.20869	S	1184.51092	592.75910	10
8	1022.46902	511.73815	H	1097.47889	549.24309	9
9	1189.46738	595.23733	N-Asn->pS...	960.41998	480.71363	8
10	1288.53580	644.77154	V	793.42162	397.21445	7
11	1451.59912	726.30320	Y	694.35321	347.68024	6
12	1564.68319	782.84523	I	531.28988	266.14858	5
13	1665.73087	833.36907	T	418.20582	209.60655	4
14	1736.76798	868.88763	A	317.15814	159.08271	3
15	1851.79492	926.40110	D	246.12102	123.56415	2

#1	c⁺	c²⁺	Seq.	z-H⁺	z-H²⁺	#2
1	131.11789	66.06258	L			16
2	260.16048	130.58388	E	1849.79185	925.39956	15
3	423.22381	212.11554	Y	1720.74926	860.87827	14
4	537.26674	269.13701	N	1557.68593	779.34660	13
5	684.33515	342.67121	F	1443.64300	722.32514	12
6	798.37808	399.69268	N	1296.57459	648.79093	11
7	885.41011	443.20869	S	1182.53166	591.76947	10
8	1022.46902	511.73815	H	1095.49963	548.25345	9
9	1187.48812	594.24770	N-Asn->nh...	958.44072	479.72400	8
10	1286.55653	643.78190	V	793.42162	397.21445	7
11	1449.61986	725.31357	Y	694.35321	347.68024	6
12	1562.70392	781.85560	I	531.28988	266.14858	5
13	1663.75160	832.37944	T	418.20582	209.60655	4
14	1734.78871	867.89800	A	317.15814	159.08271	3
15	1849.81566	925.41147	D	246.12102	123.56415	2

Figure S5. Bottom-up MS/MS fragmentation of trypsin digested peptides confirming incorporation of (A) pSer at site N150 of 2x-TAG sfGFP made using the pSer GCE system and (B) nhpSer at site N150 of the 2x-TAG sfGFP using the nhpSer Frb-v1 GCE system.

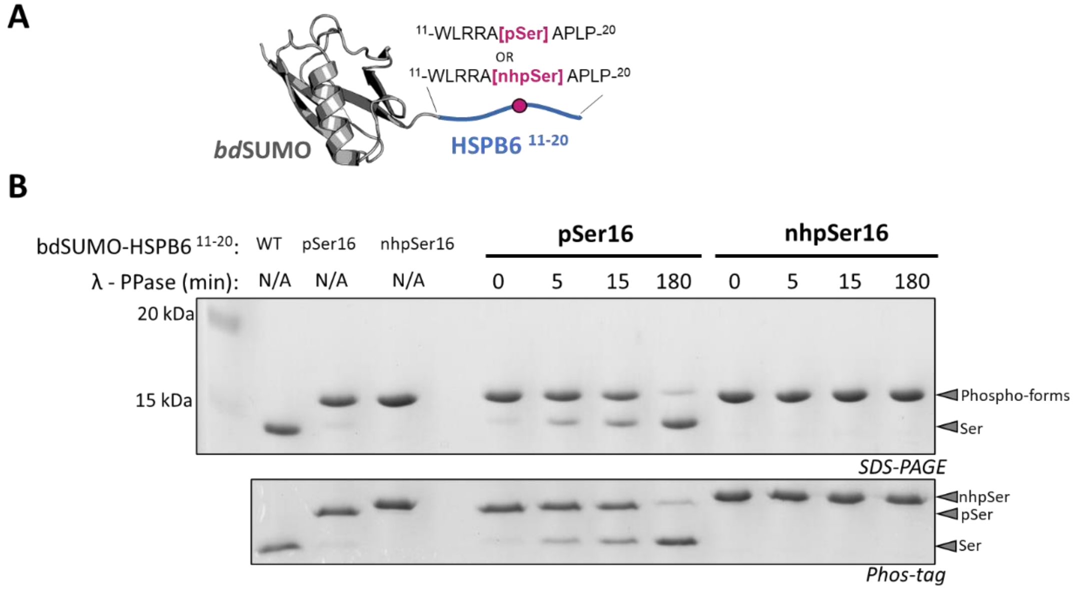

Figure S6. nhpSer protein made via Frb-v1 GCE is resistant to phosphatase activity. (A) pSer or nhpSer (as well as Ser) was incorporated into site S16 of a HSPB6 peptide consisting of residues 11-20, which was fused at its N-terminus to *bd*SUMO. (B) SDS-PAGE (top) and Phos-tag (bottom) of purified *bd*SUMO-HSPB6$^{11\text{-}20}$ proteins with Ser16, pSer16 and nhpSer16 are shown in the first three lanes. Upon incubation with λ-phosphatase (PPase), the pSer16 containing protein was hydrolyzed as evidenced by it migrating with the electrophoretic mobility of wild-type protein, while the nhpSer protein mobility remained constant. Interestingly, the phospho-proteins migrate more slowly on SDS-PAGE compared to wild-type. Slower electrophoretic mobility of phospho-proteins compared to non-phosphorylated proteins in SDS-PAGE has been well-documented previously.[3] Reasons for this are not well understood but presumably the negatively charged phospho-group limits SDS binding capacity to the protein, lowering its charge to mass ratio and causing it to migrate more slowly.

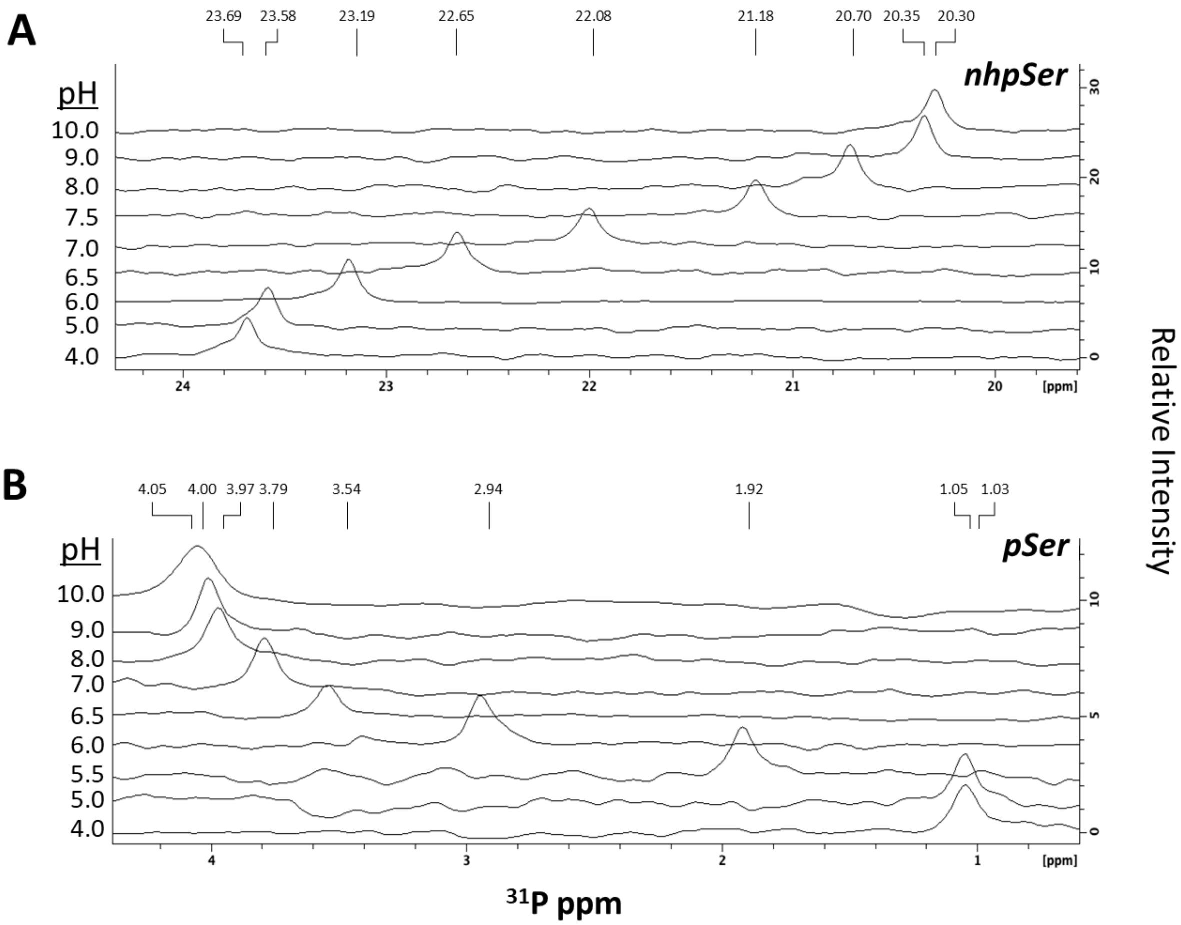

Figure S7. ^{31}P NMR spectra of *bd*SUMO-HSPB6$^{11\text{-}20}$ containing (A) nhpSer and (B) pSer at site S16 of HSPB6$^{11\text{-}20}$ at pH's ranging from 4.0 to 10.0. Chemical shifts are indicated above each peak.

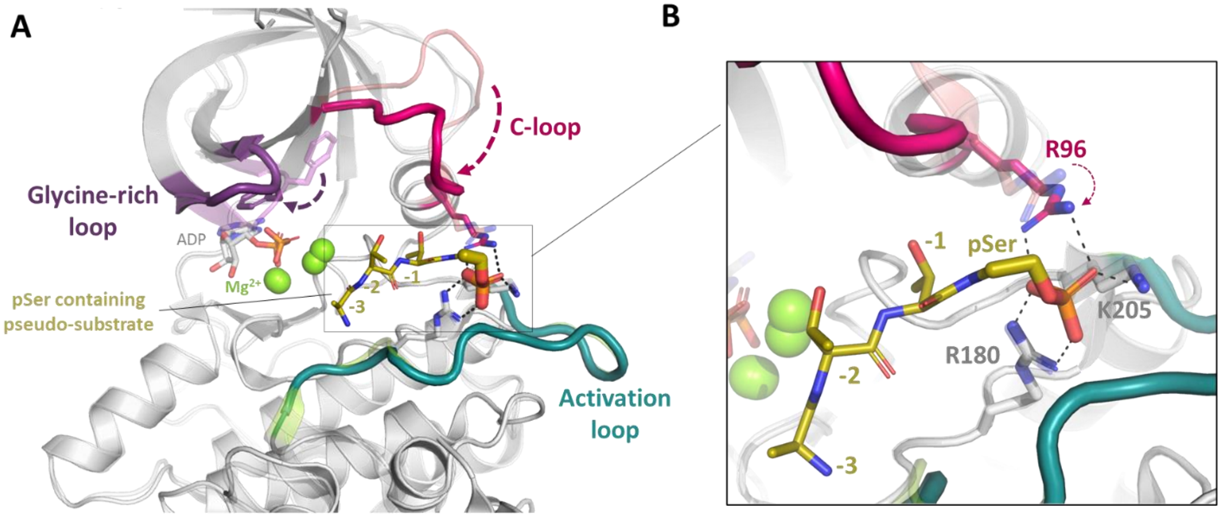

Figure S8. Mechanism of GSK3β activation upon binding to pSer-containing substrates. (A) Overlayed structures of apo GSK3β (PDB 1pyx) and GSK3β bound to a phosphorylated pseudo-substrate (PDB 4nm3) showing that binding of the peptide (yellow) induces conformational changes in the C-loop (pink) and glycine rich loop (purple). (B) Zoom in showing that the movement of the C-loop upon pSer-substrate binding positions Arg96 to coordinate the phospho group of the substrate peptide, and places the -4 residue (not resolved in crystal structure) in the proximity of the active site where magnesium (green spheres) coordinates ATP.

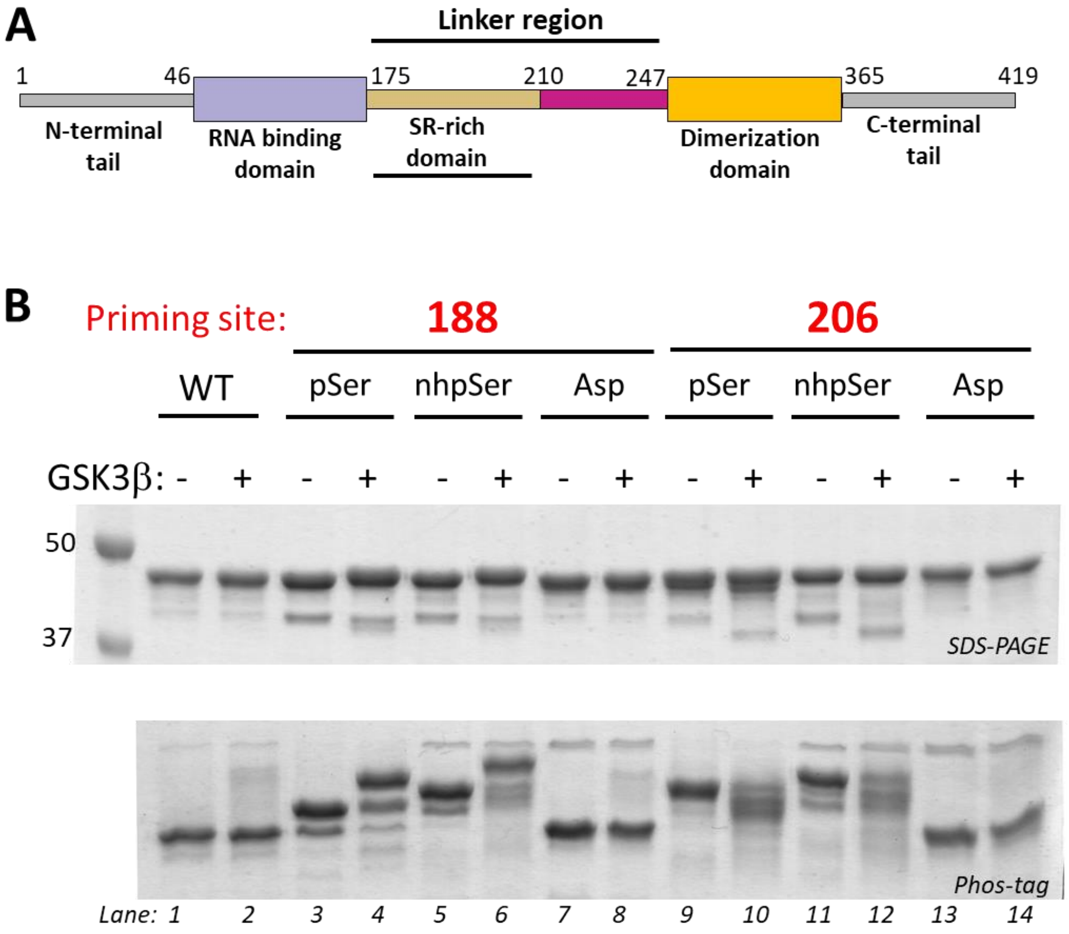

Figure S9. nhpSer can mimic pSer-dependent GSK3β phosphorylation of full-length SARS-CoV-2 nucleocapsid protein. (A) The SARS-CoV-2 Nucleocapsid Phosphoprotein (Np) contains a Serine-Arginine rich region (SR) in its linker region (Linker-Np, residues 175-247) that connects the N-terminal RNA binding domain and the C-terminal dimerization domain. (B) Ser, Asp, pSer and nhpSer were incorporated at sites S188 and S206 in full-length Np, purified, and mixed with ATP, Mg^{2+}, and GSK3β as described in the Methods. SDS-PAGE and Phos-tag gels of reaction products confirm phosphorylation of Linker-Np by GSK3β only in the pSer and nhpSer primed Linker-Np proteins, and not the Ser or Asp proteins, as evidenced by the gel-shifts observed in the Phos-tag gel.

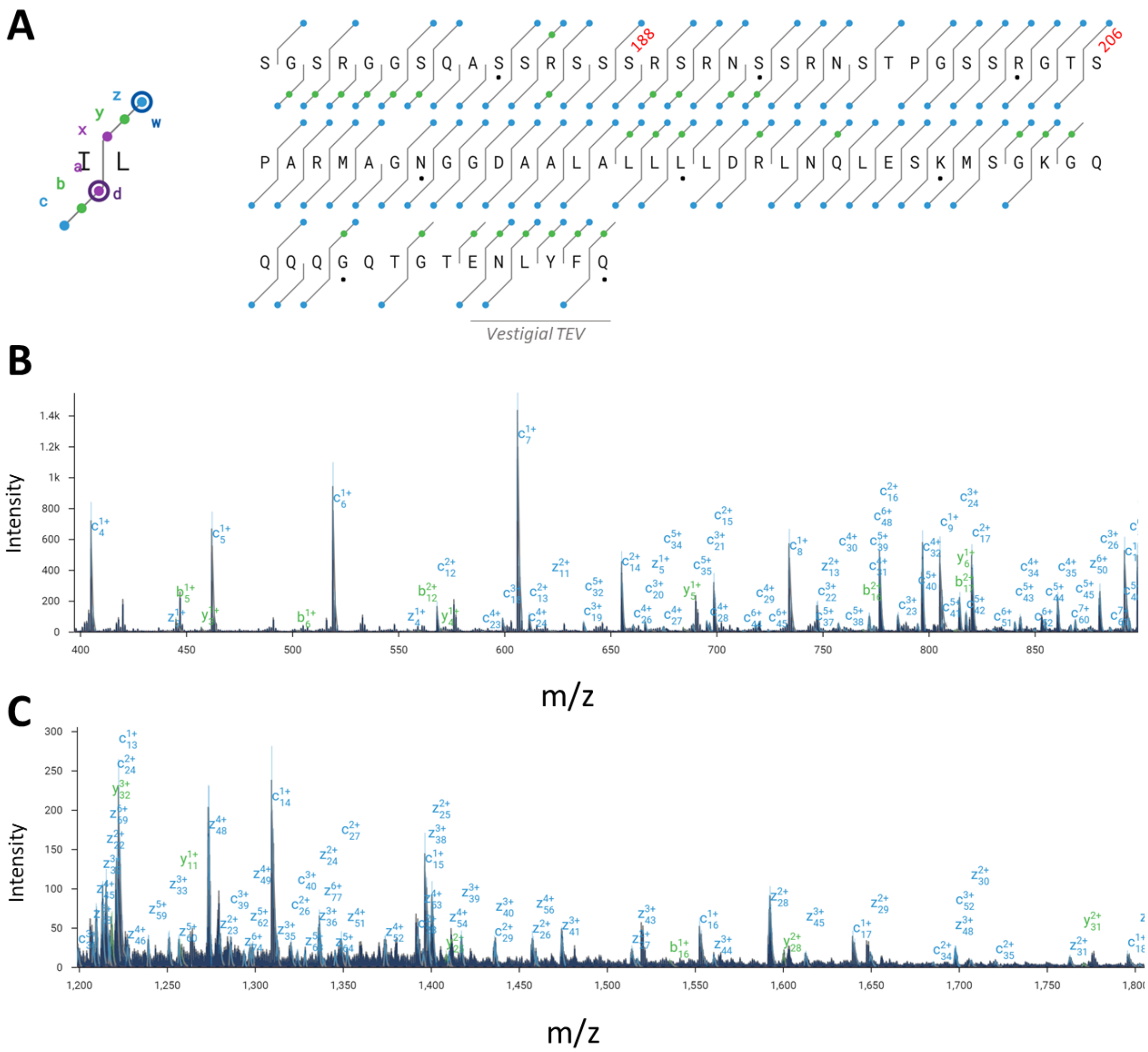

Figure S10 MS/MS analysis of wild-type Linker-Np protein. (A) Sequence map depicting the detected ion fragments. The blue circles indicate c and z ions, and the green circles indicate b and y ions as shown on the left. Ser188 and Ser206 are labeled accordingly. Block dots represent every 10 residues. The last six residues (ENLYFQ) are part of the TEV protease recognition sequence remaining after removal of sfGFP (Fig. 5C). The top-down analysis yielded an overall sequence coverage of 91%. (B-C). MS/MS fragmentation mass spectra with peaks labeled according to their assigned identity.

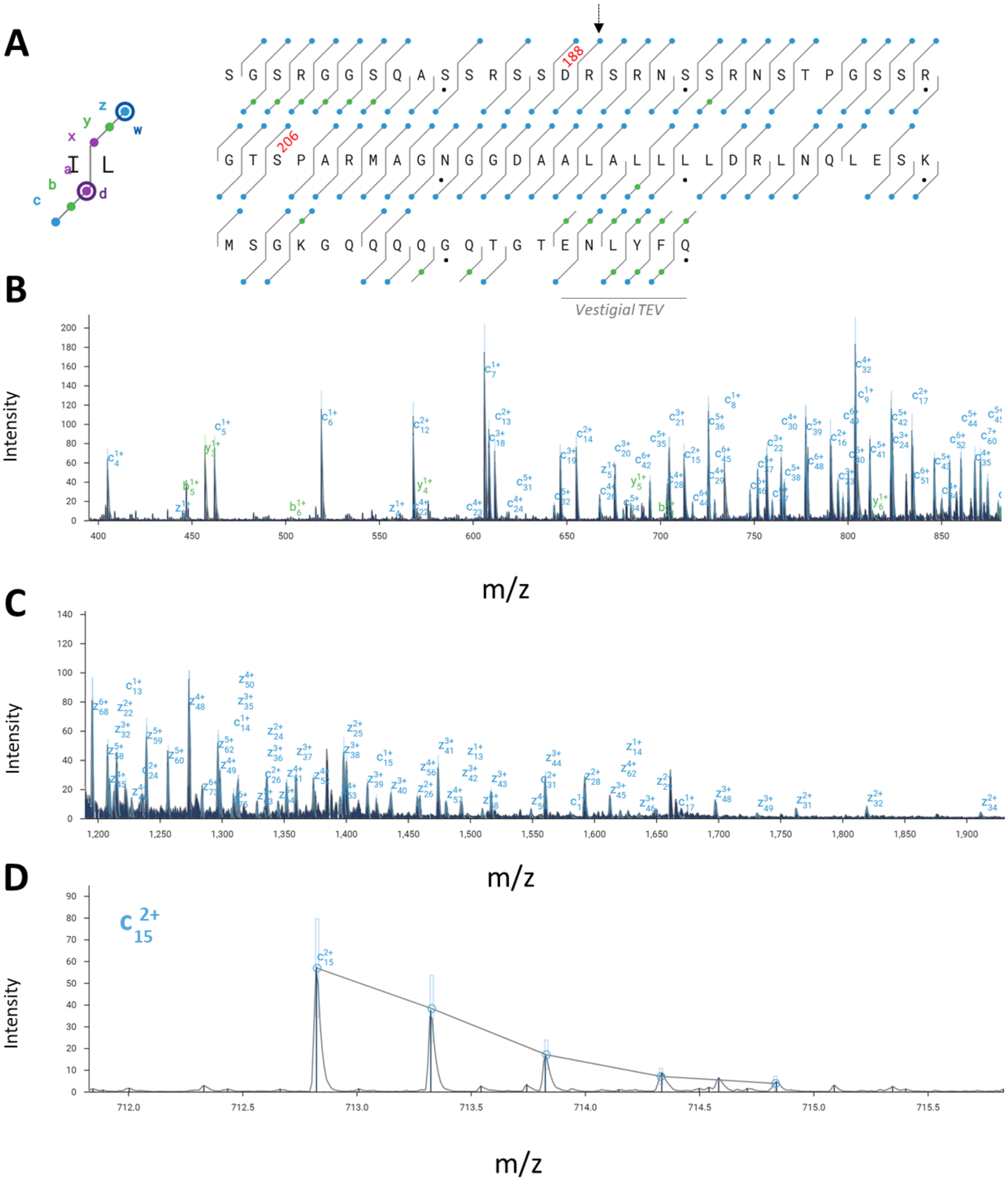

Figure S11. MS/MS analysis of S188D Linker-Np protein. (A) Sequence map depicting the detected ion fragments. The blue circles indicate c and z ions, and the green circles indicate b and y ions as shown on the left. Asp188 and Ser206 are labeled accordingly. Block dots represent every 10 residues. The last six residues (ENLYFQ) are part of the TEV protease recognition sequence remaining after removal of sfGFP (Fig. 5C). The top-down analysis yielded an overall sequence coverage of 87%. Black arrow points to the fragmented peptide ion shown in panel D. (B-C). Representative MS/MS fragmentation mass spectra with peaks labeled according to their assigned identity. (D) Zoom in of the c^{2+}/15 fragmented peptide ion confirms the location of Asp at position 188.

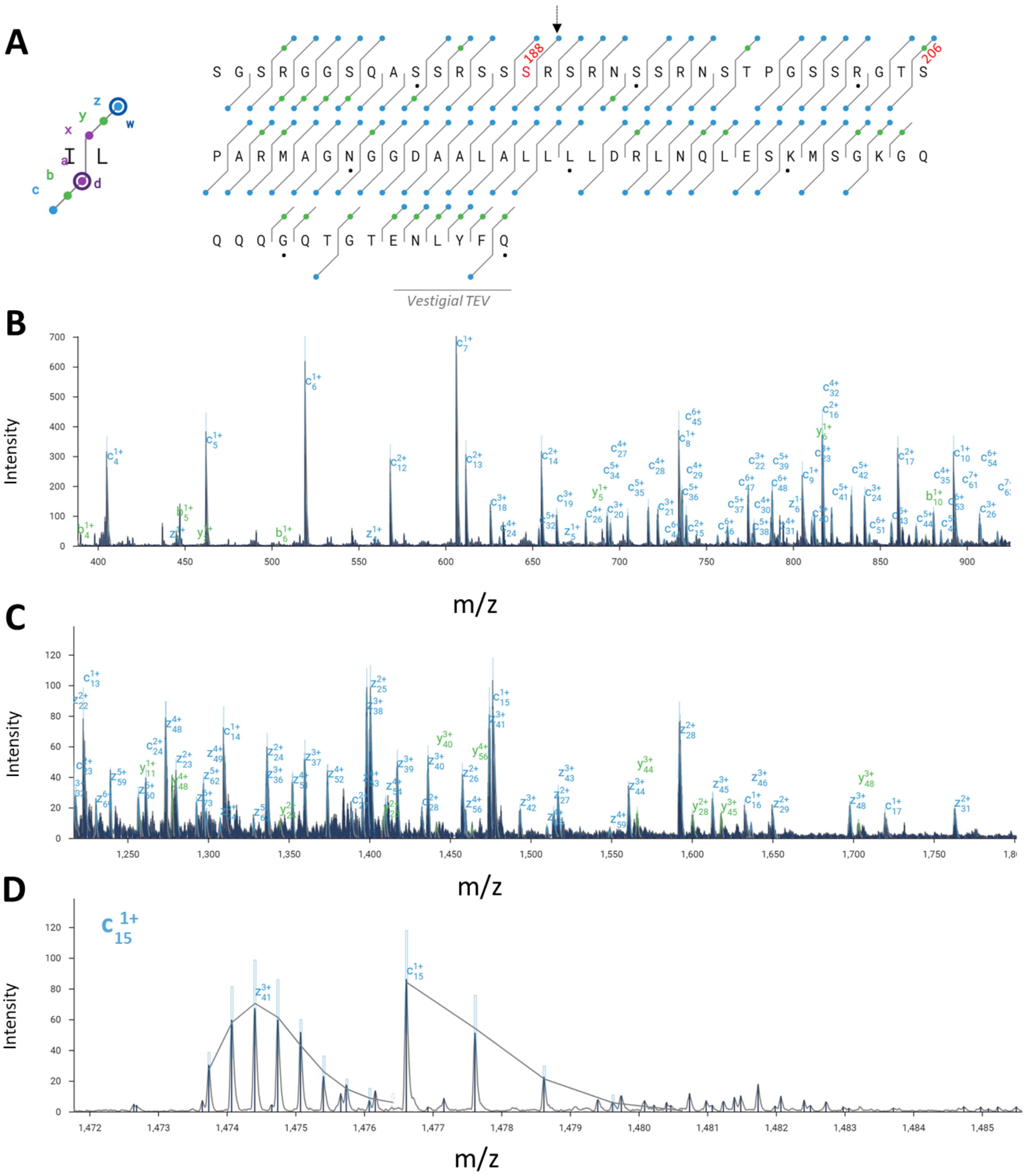

Figure S12. MS/MS analysis of pSer188 Linker-Np protein. (A) Sequence map depicting the detected ion fragments. The blue circles indicate c and z ions, and the green circles indicate b and y ions as shown on the left. Ser188 and Ser206 positions are labeled accordingly. Block dots represent every 10 residues. The last six residues (ENLYFQ) are part of the TEV protease recognition sequence remaining after removal of sfGFP (Fig. 5C). The top-down analysis yielded an overall sequence coverage of 87%. Black arrow points to the fragmented peptide ion shown in panel D. (B-C). Representative MS/MS fragmentation mass spectra with peaks labeled according to their assigned identity. (D) Zoom in of the $c^{1+}/15$ fragmented peptide ion confirms the location of pSer at position 188.

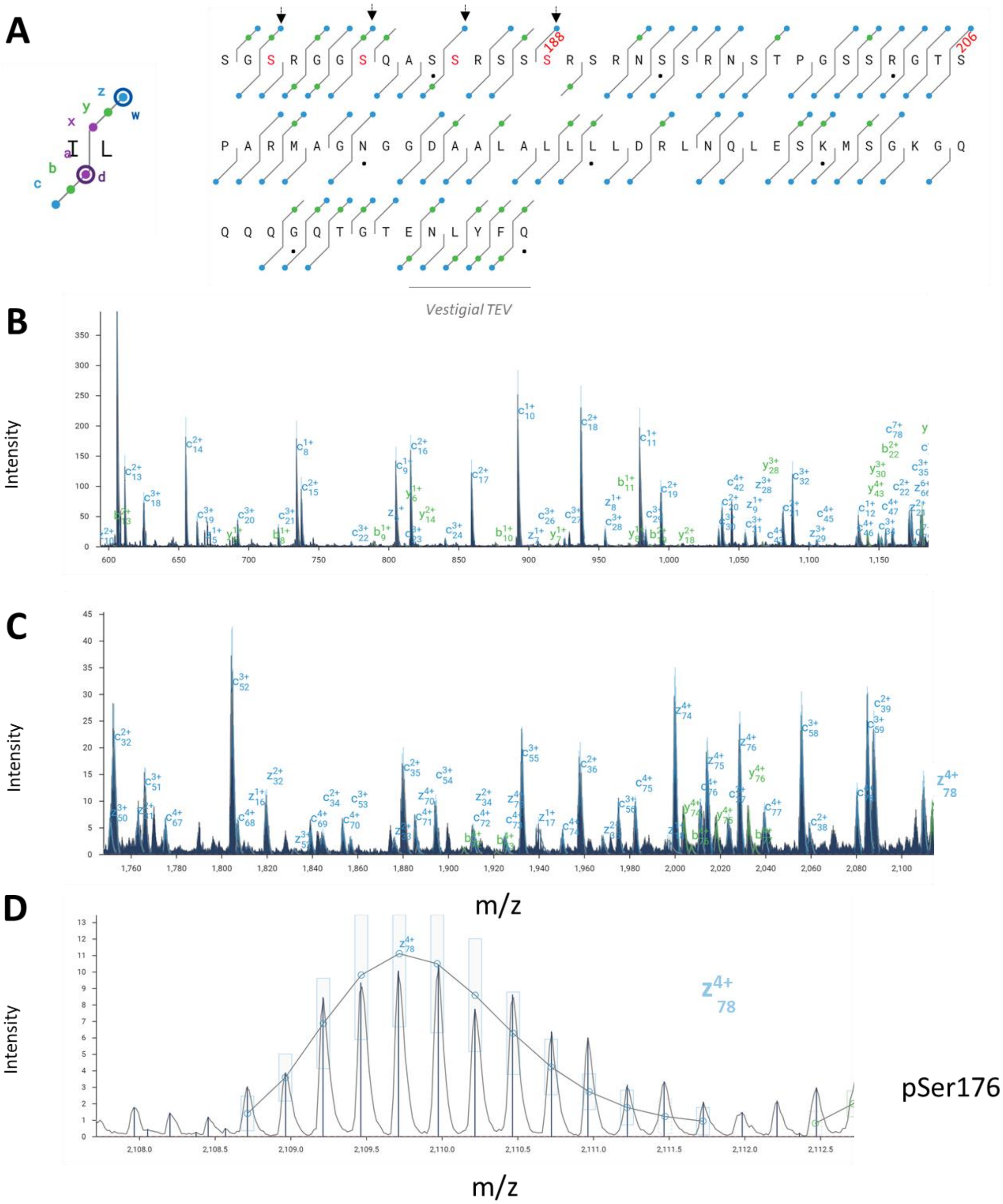

(continued on next page)

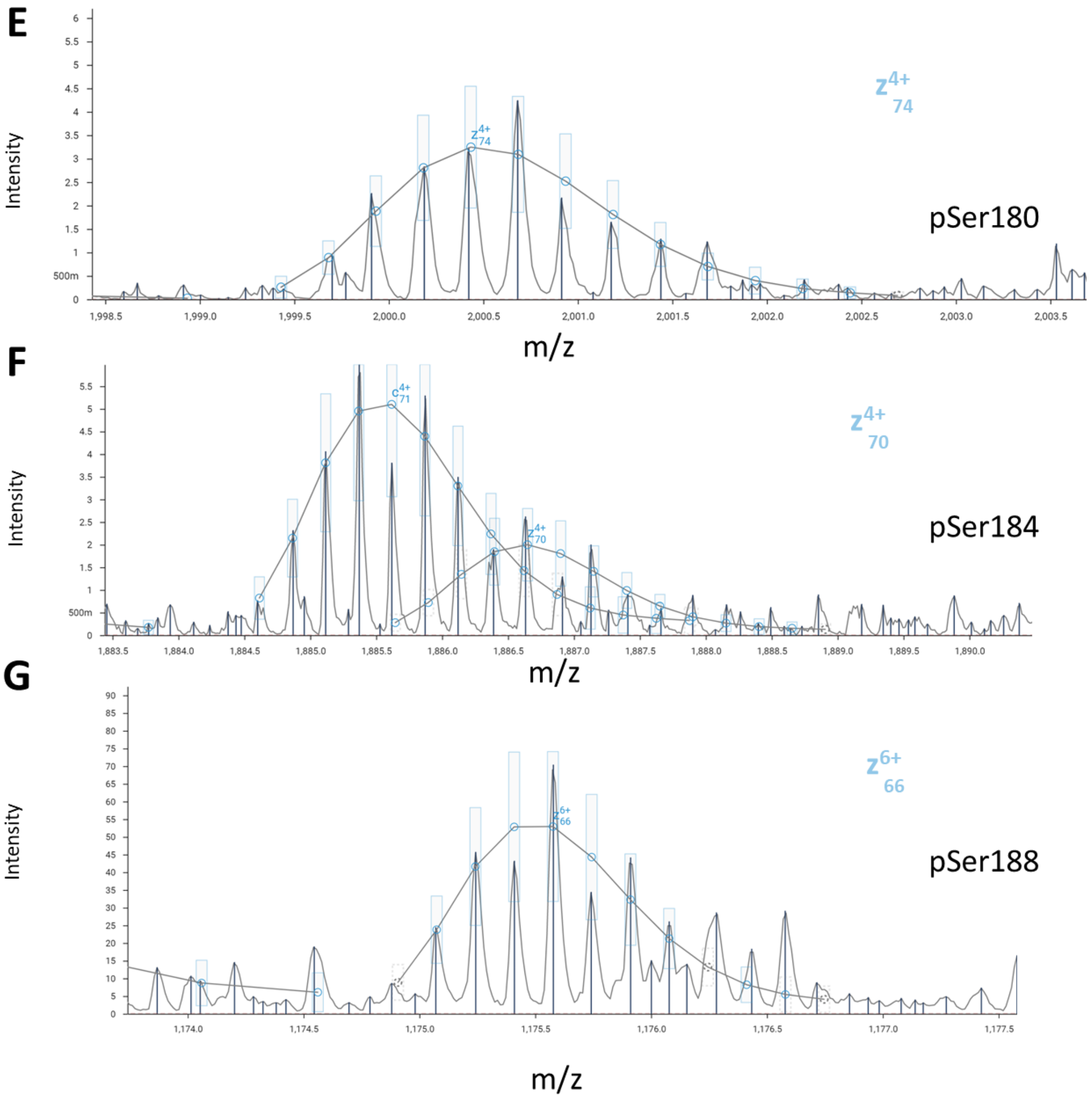

Figure S13. MS/MS analysis of pSer188 Linker-Np protein after reaction with GSK3β. (A) Sequence map depicting the detected ion fragments. The blue circles indicate c and z ions, and the green circles indicate b and y ions as shown on the left. Ser188 and Ser206 positions are labeled accordingly. Block dots represent every 10 residues. The last six residues (ENLYFQ) are part of the TEV protease recognition sequence remaining after removal of sfGFP (Fig. 5C). The top-down analysis yielded an overall sequence coverage of 80%. Black arrow points to the fragmented peptide ions shown in panels D-G. (B-C). Representative MS/MS fragmentation mass spectra with peaks labeled according to their assigned identity. (D-G) Zoom in of the z^{4+}/78, z^{4+}/74, z^{4+}/70, z^{6+}/66 fragmented peptide ion confirms the location of pSer at position 176, 180, 184 and 188, respectively.

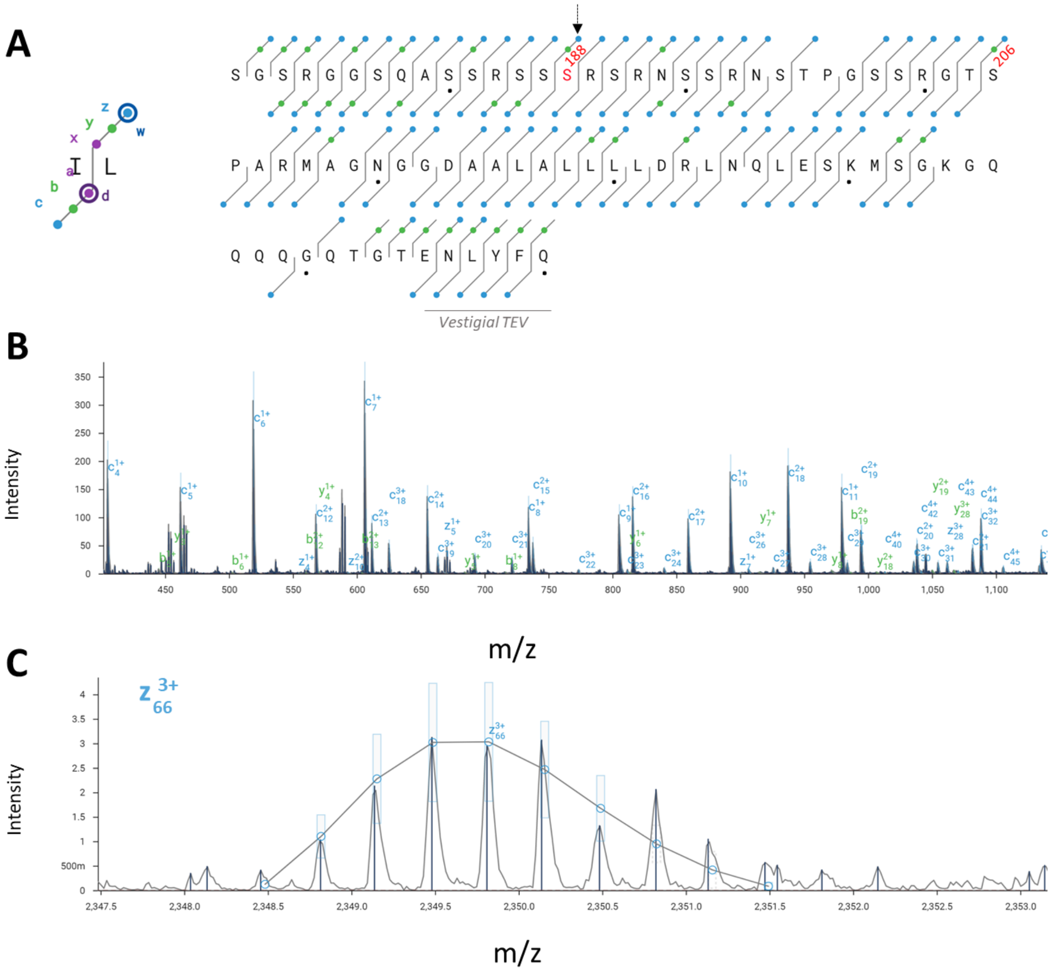

Figure S14. MS/MS analysis of nhpSer188 Linker-Np protein. (A) Sequence map depicting the detected ion fragments. The blue circles indicate c and z ions, and the green circles indicate b and y ions as shown on the left. Ser188 and Ser206 positions are labeled accordingly. Block dots represent every 10 residues. The last six residues (ENLYFQ) are part of the TEV protease recognition sequence remaining after removal of sfGFP (Fig. 5C). The top-down analysis yielded an overall sequence coverage of 87%. Black arrow points to the fragmented peptide ion shown in panel C. (B). Representative MS/MS fragmentation mass spectra with peaks labeled according to their assigned identity. (D) Zoom in of the z^{3+}/66 fragmented peptide ion confirms the location of nhpSer at position 188.

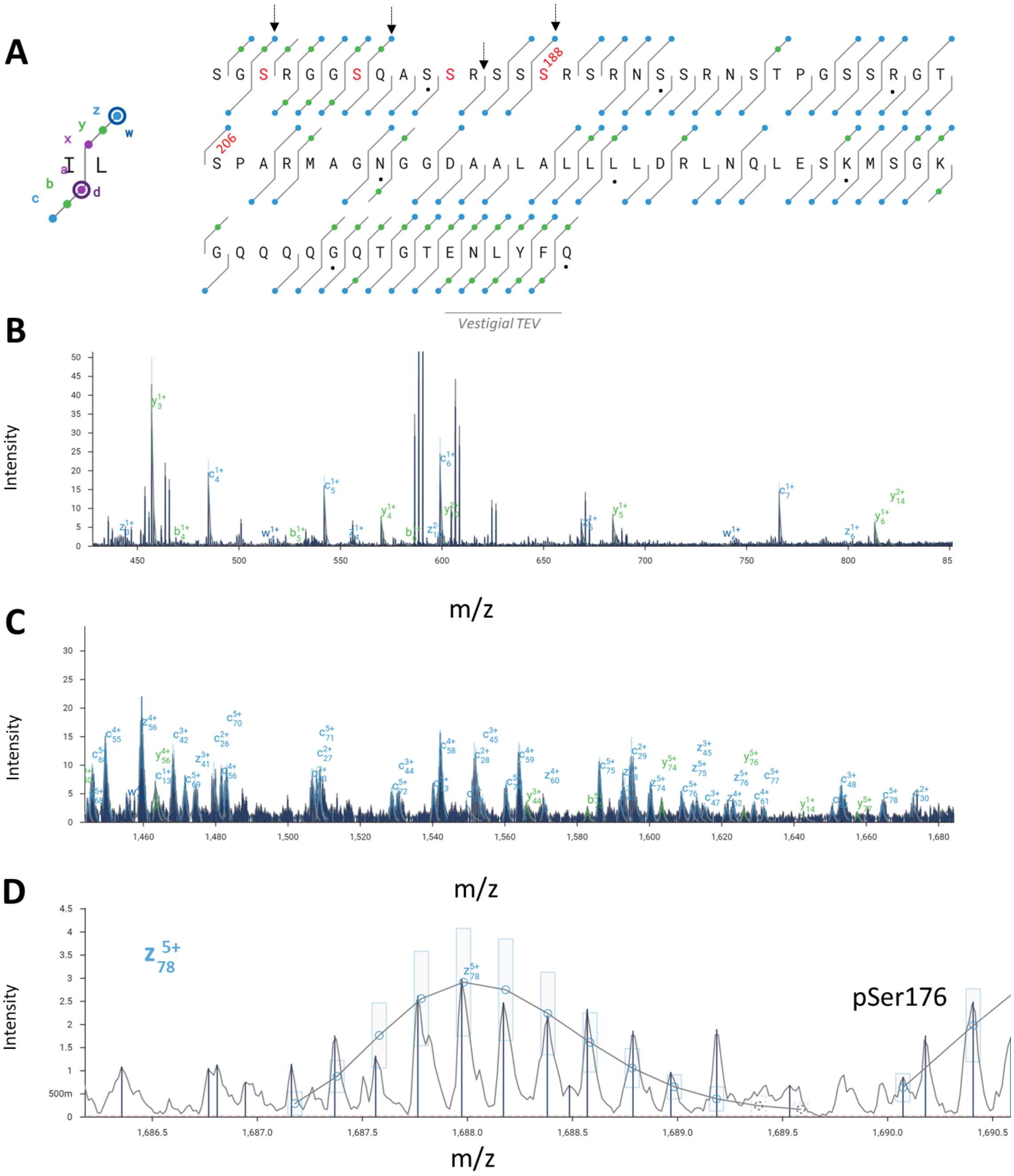

(continued on next page)

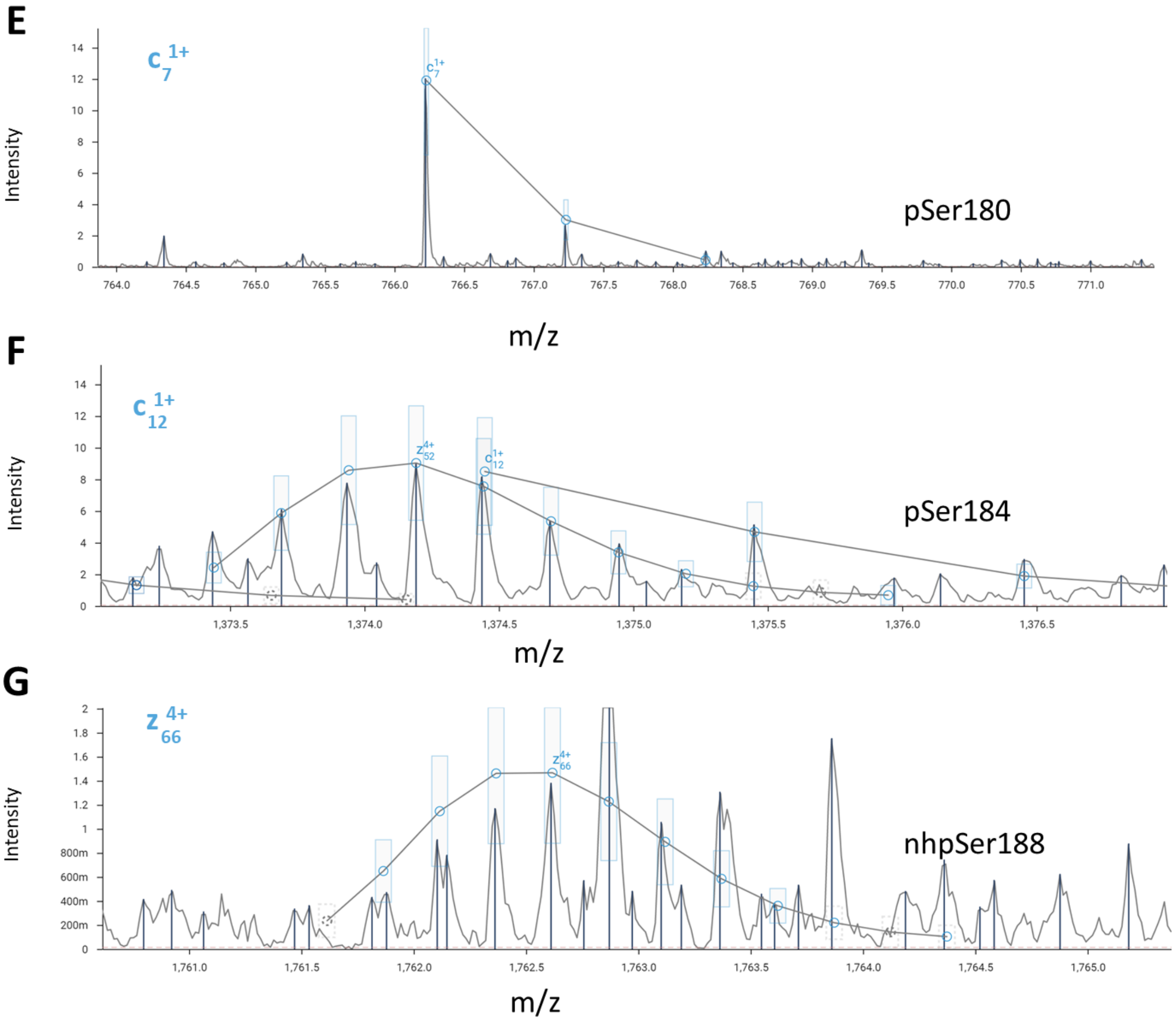

Figure S15. MS/MS analysis of nhpSer188 Linker-Np protein after reaction with GSK3β. (A) Sequence map depicting the detected ion fragments. The blue circles indicate c and z ions, and the green circles indicate b and y ions as shown on the left. Ser188 and Ser206 positions are labeled accordingly. Block dots represent every 10 residues. The last six residues (ENLYFQ) are part of the TEV protease recognition sequence remaining after removal of sfGFP (Fig. 5C). The top-down analysis yielded an overall sequence coverage of 82%. Black arrow points to the fragmented peptide ions shown in panels D-G. (B-C). Representative MS/MS fragmentation mass spectra with peaks labeled according to their assigned identity. (D-G) Zoom in of the z^{5+}/78, c^{1+}/7, c^{1+}/12, z^{4+}/66 fragmented peptide ion confirms the location of pSer at position 176, 180, and 184, and nhpSer at188, respectively.

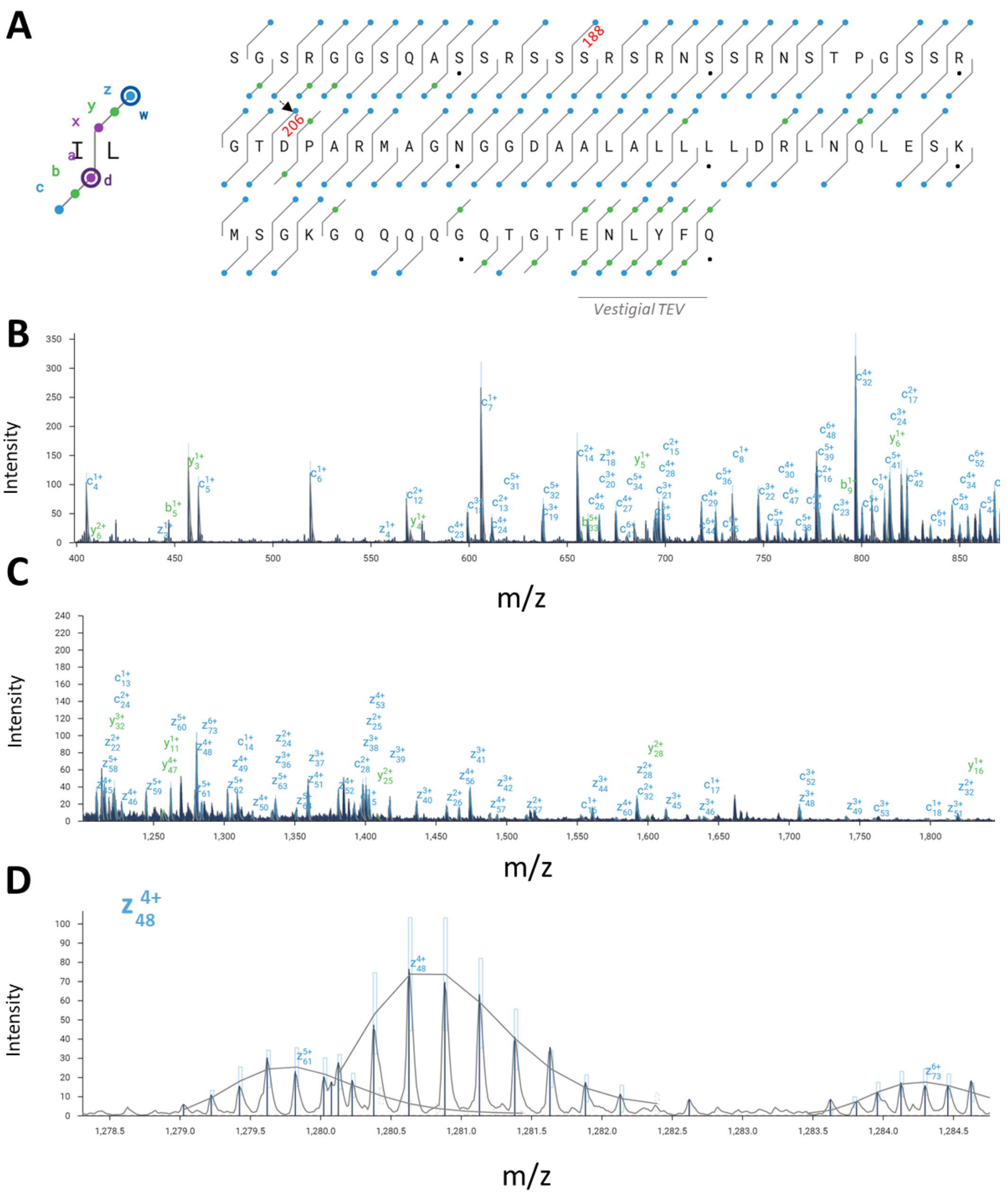

Figure S16. MS/MS analysis of S206D Linker-Np protein. (A) Sequence map depicting the detected ion fragments. The blue circles indicate c and z ions, and the green circles indicate b and y ions as shown on the left. Ser188 and Asp206 are labeled accordingly. Block dots represent every 10 residues. The last six residues (ENLYFQ) are part of the TEV protease recognition sequence remaining after removal of sfGFP (Fig. 5C). The top-down analysis yielded an overall sequence coverage of 92%. Black arrow points to the fragmented peptide ion shown in panel D. (B-C). Representative MS/MS fragmentation mass spectra with peaks labeled according to their assigned identity. (D) Zoom in of the z^{4+}/48 fragmented peptide ion confirms the location of Asp at position 206.

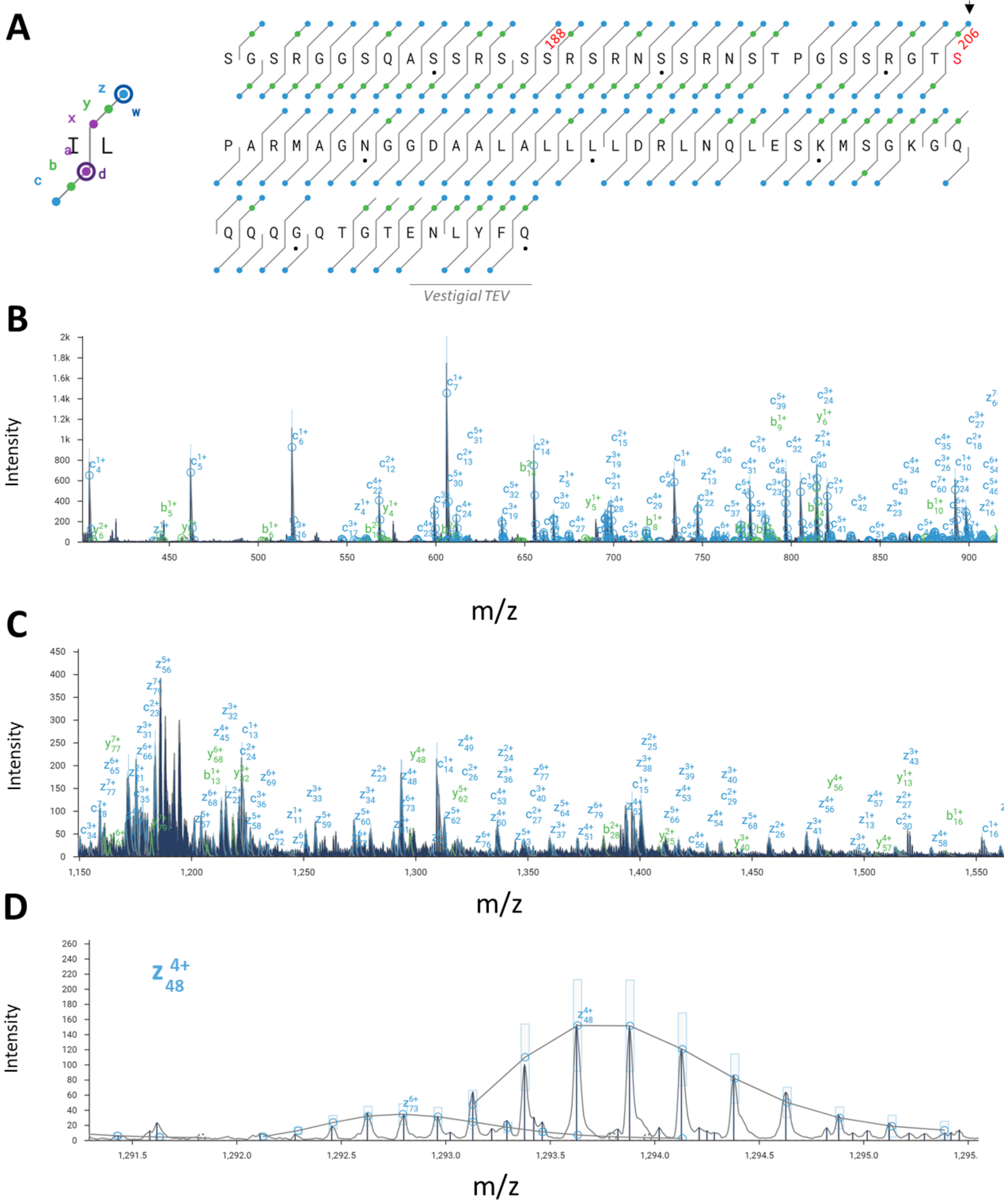

Figure S17. MS/MS analysis of pSer206 Linker-Np protein. (A) Sequence map depicting the detected ion fragments. The blue circles indicate c and z ions, and the green circles indicate b and y ions as shown on the left. Ser188 and Ser206 positions are labeled accordingly. Block dots represent every 10 residues. The last six residues (ENLYFQ) are part of the TEV protease recognition sequence remaining after removal of sfGFP (Fig. 5C). The top-down analysis yielded an overall sequence coverage of 96%. Black arrow points to the fragmented peptide ion shown in panel D. (B-C). Representative MS/MS fragmentation mass spectra with peaks labeled according to their assigned identity. (D) Zoom in of the z^{4+}/48 fragmented peptide ion confirms the location of pSer at position 206.

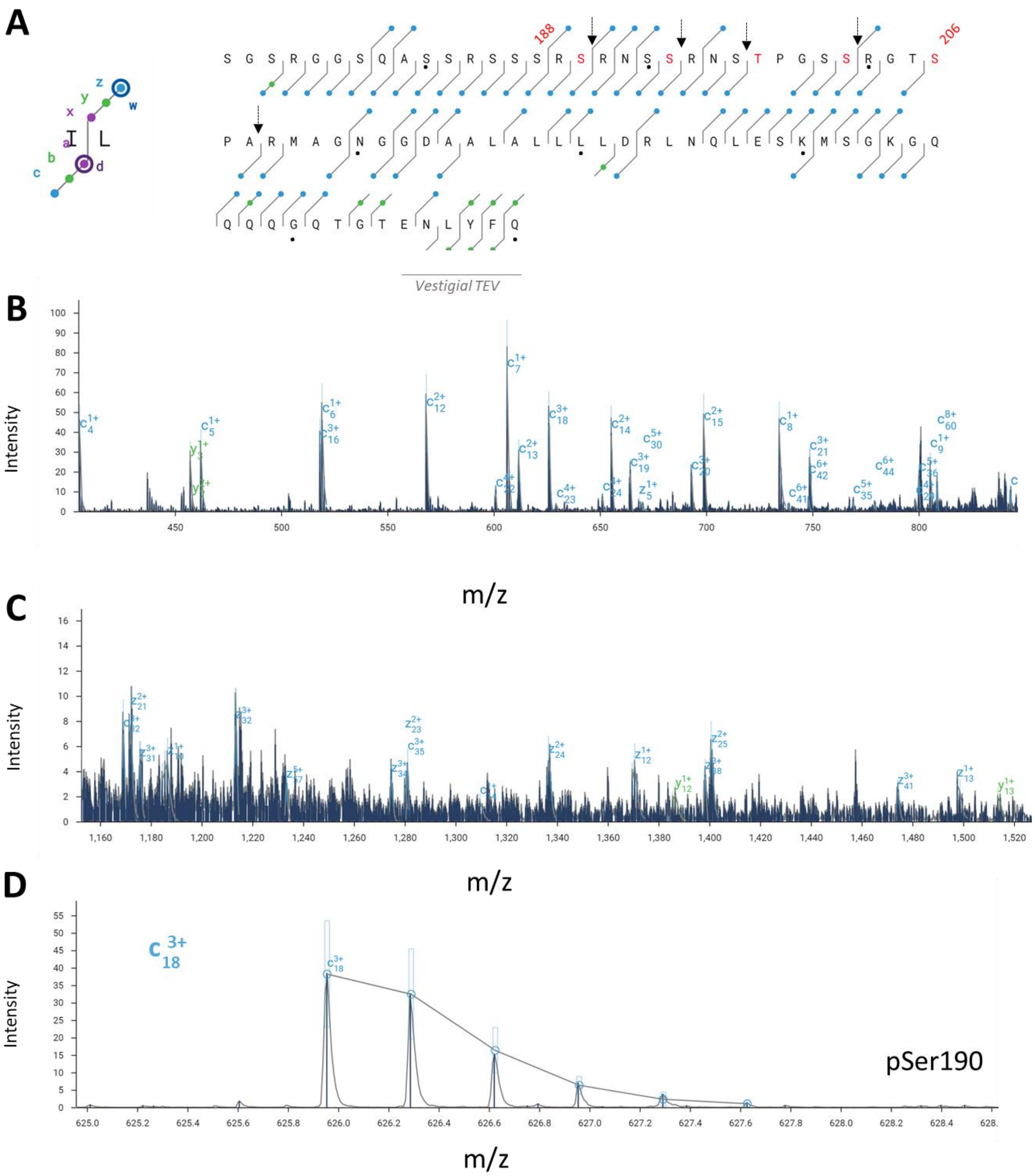

(continued on next page)

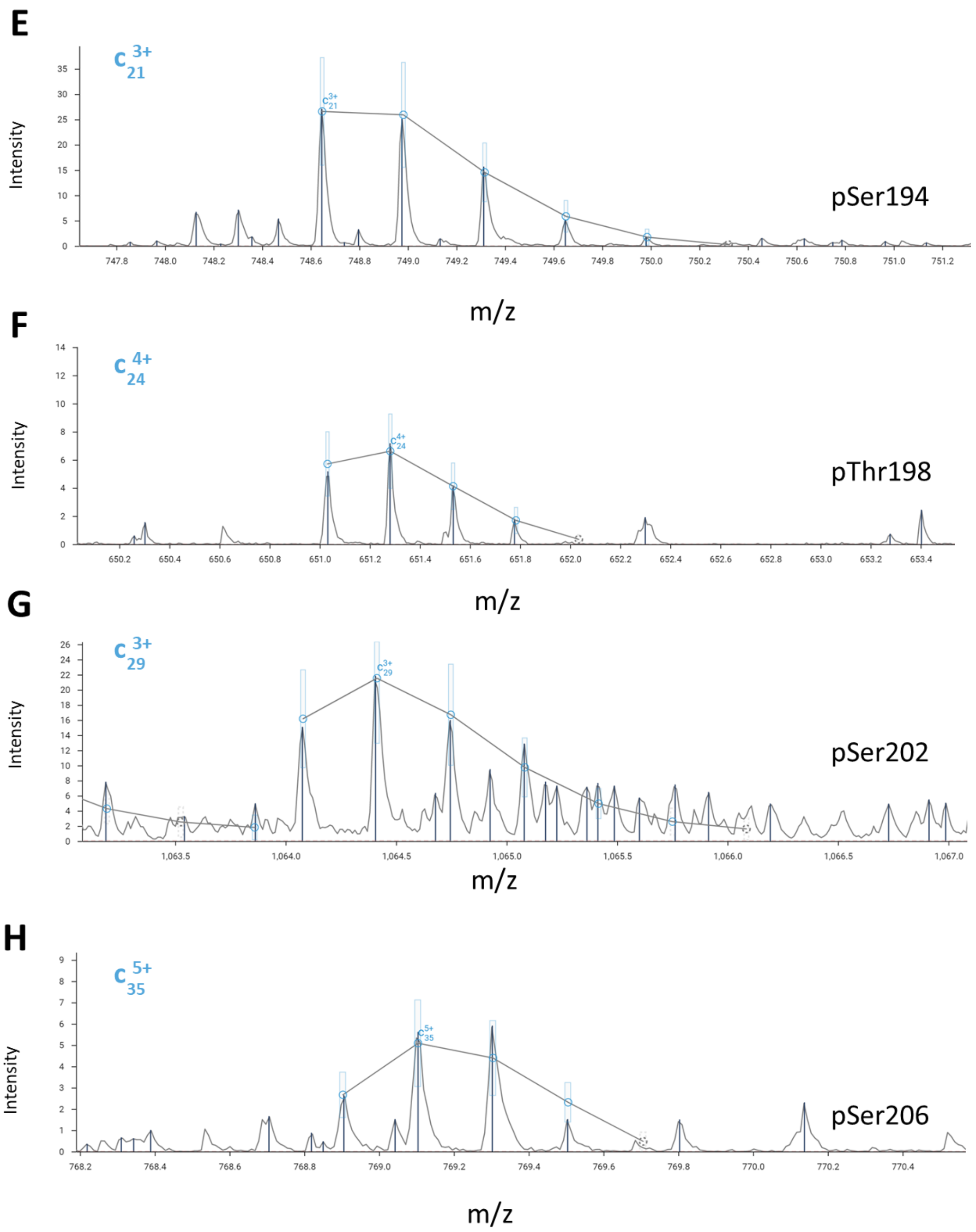

Figure S18. MS/MS analysis of pSer206 Linker-Np protein after reaction with GSK3β. (A) Sequence map depicting the detected ion fragments. The blue circles indicate c and z ions, and the green circles indicate b and y ions as shown on the left. Ser188 and Ser206 positions are labeled accordingly. Block dots represent every 10 residues. The last six residues (ENLYFQ) are part of the TEV protease recognition sequence remaining after removal of sfGFP (Fig. 5C). The top-down analysis yielded an overall sequence coverage of 78%. Black arrow points to the fragmented peptide ions shown in panels D-H. (B-C). Representative MS/MS fragmentation mass spectra with peaks labeled according to their assigned identity. (D-H) Zoom in of the c^{3+}/18, c^{3+}/21, c^{4+}/24, c^{3+}/29, and c^{5+}/35 fragmented peptide ion confirms the location of phosphorylation at positions 190, 194, 198, 202 and 206 respectively.

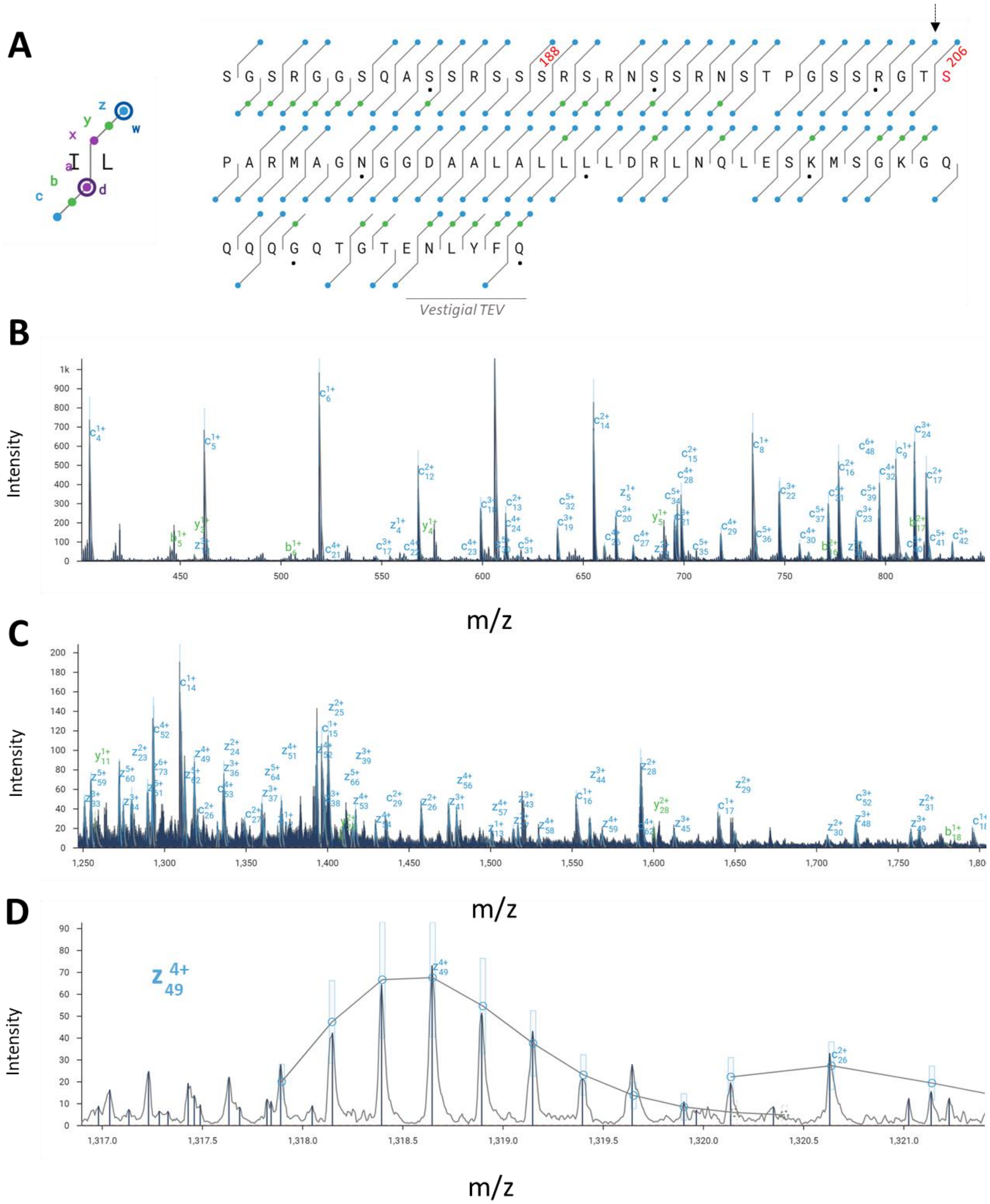

Figure S19. MS/MS analysis of nhpSer206 Linker-Np protein. (A) Sequence map depicting the detected ion fragments. The blue circles indicate c and z ions, and the green circles indicate b and y ions as shown on the left. Ser188 and Ser206 positions are labeled accordingly. Block dots represent every 10 residues. The last six residues (ENLYFQ) are part of the TEV protease recognition sequence remaining after removal of sfGFP (Fig. 5C). The top-down analysis yielded an overall sequence coverage of 94%. Black arrow points to the fragmented peptide ion shown in panel D. (B-C). Representative MS/MS fragmentation mass spectra with peaks labeled according to their assigned identity. (D) Zoom in of the z^{4+}/49 fragmented peptide ion confirms the location of nhpSer at position 206.

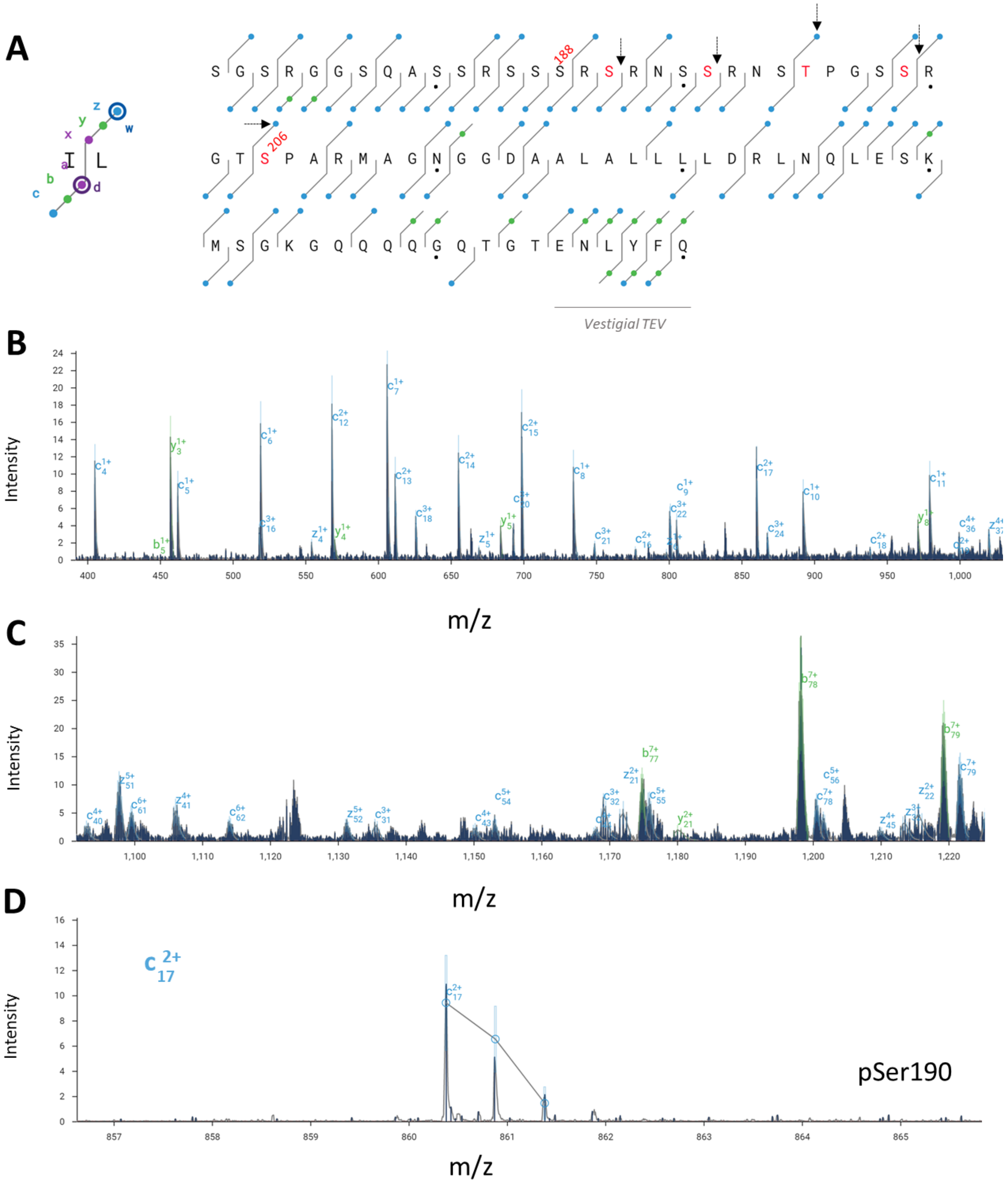

(continued on next page)

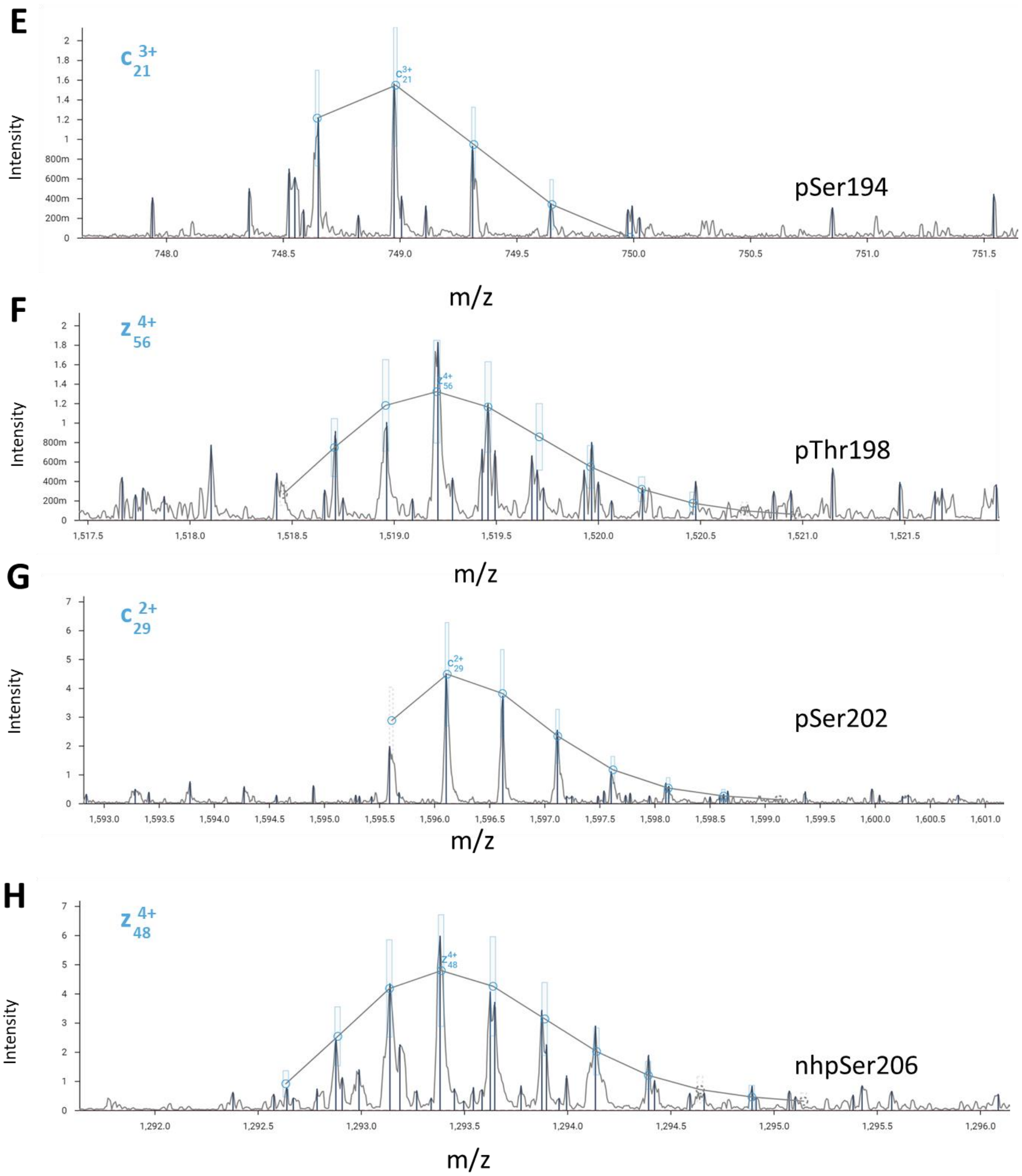

Figure S20. MS/MS analysis of nhpSer206 Linker-Np protein after reaction with GSK3β. (A) Sequence map depicting the detected ion fragments. The blue circles indicate c and z ions, and the green circles indicate b and y ions as shown on the left. Ser188 and Ser206 positions are labeled accordingly. Block dots represent every 10 residues. The last six residues (ENLYFQ) are part of the TEV protease recognition sequence remaining after removal of sfGFP (Fig. 5C). The top-down analysis yielded an overall sequence coverage of 78%. Black arrow points to the fragmented peptide ions shown in panels D-H. (B-C). Representative MS/MS fragmentation mass spectra with peaks labeled according to their assigned identity. (D-H) Zoom in of the c^{2+}/17, c^{3+}/21, z^{4+}/56, c^{2+}/29 and z^{4+}/48 fragmented peptide ion confirms the location of phosphorylation at positions 190, 194, 198, and 202, and nhpSer at 206, respectively.

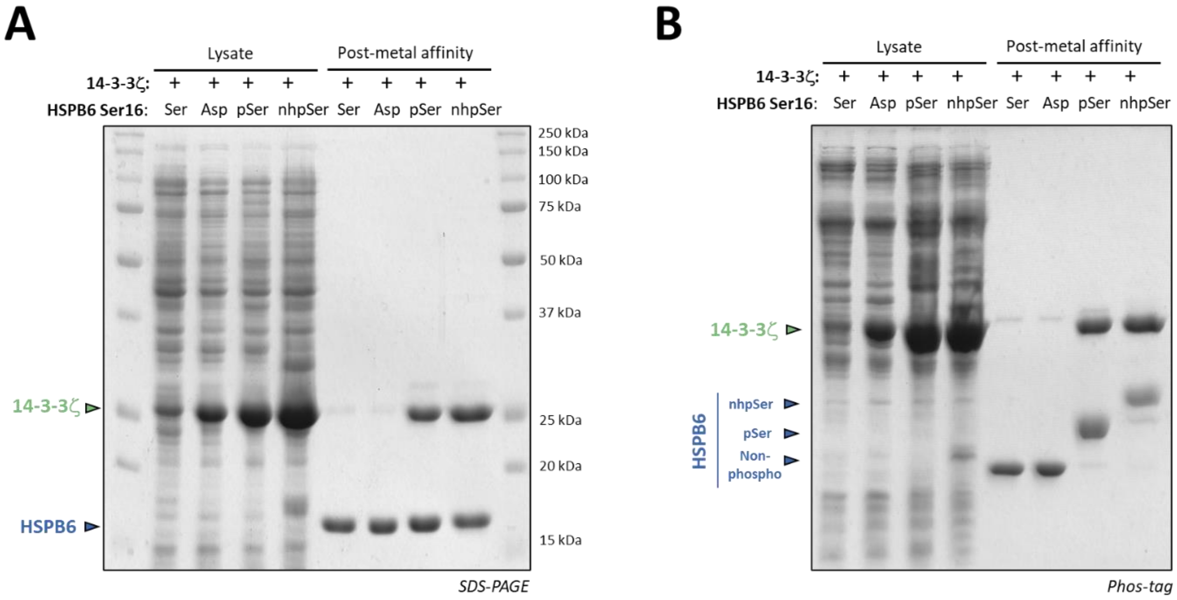

Figure S21. SDS-PAGE and Phos-tag gel analyses of 14-3-3ζ/HSPB6 (full-length) pull down experiments shown in Fig. 6B. (A) SDS-PAGE of soluble cell lysates confirm expression of 14-3-3ζ in all expressions with HSPB6, indicating that the lack of 14-3-3ζ in the purified wild-type (Ser) and S16D (Asp) HSPB6 samples was due to their inability to complex with 14-3-3ζ. (B) Phos-tag gel of the same samples shown in panel A, confirming pSer and nhpSer incorporation as shown in Fig. 6B.

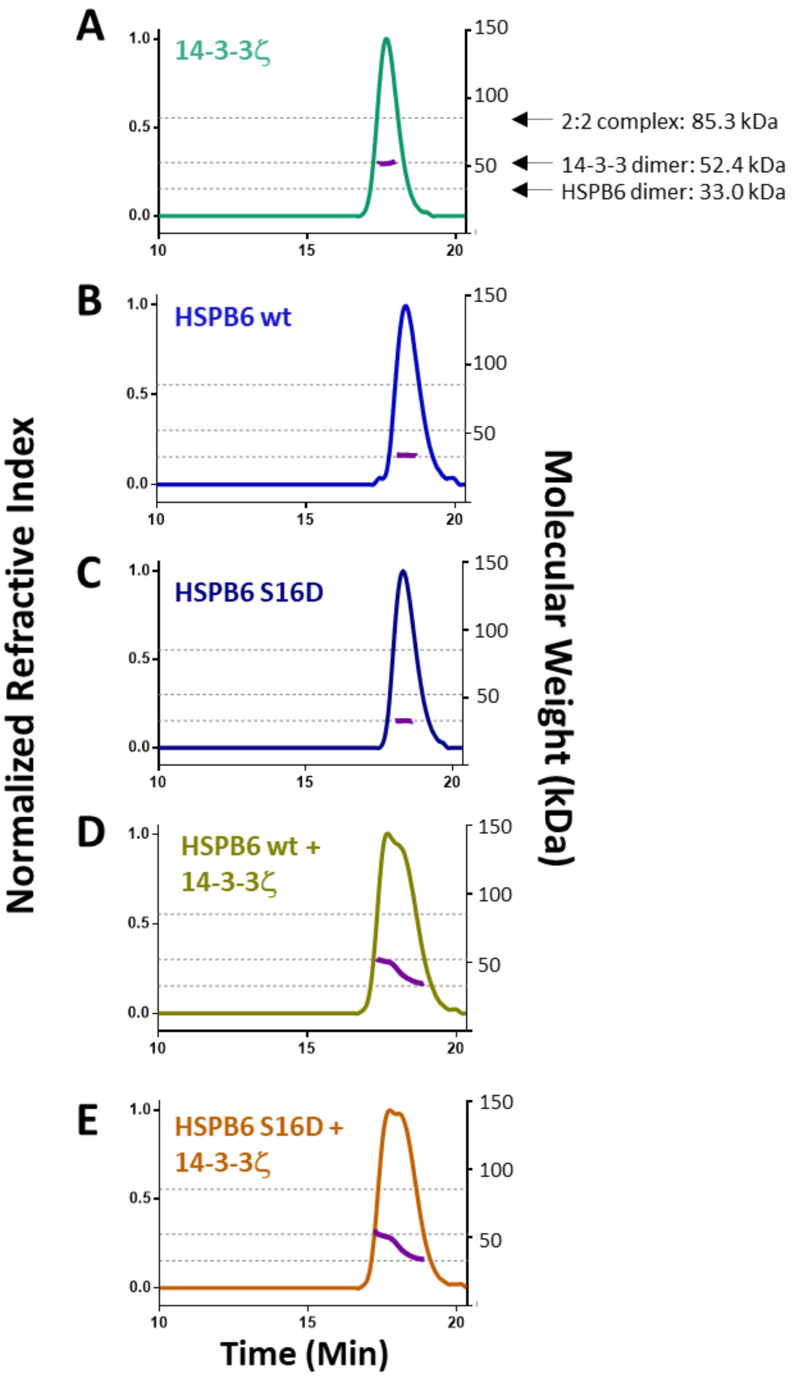

Figure S22. SEC-MALS analyses of wild-type and S16D HSPB6 with 14-3-3ζ confirm they do not form a stable complex. (A) wild-type 14-3-3ζ only, (B) full-length HSPB6 wt only, (C) HSPB6 S16D only, (D) an equimolar mixture of 14-3-3ζ and HSPB6 wt, (E) and an equimolar mixture of 14-3-3ζ and HSPB6 S16D. Dotted lines from top to bottom in each panel correspond to the theoretical molecular weights of the 2:2 complex, the 14-3-3ζ dimer and the HSPB6 dimer.

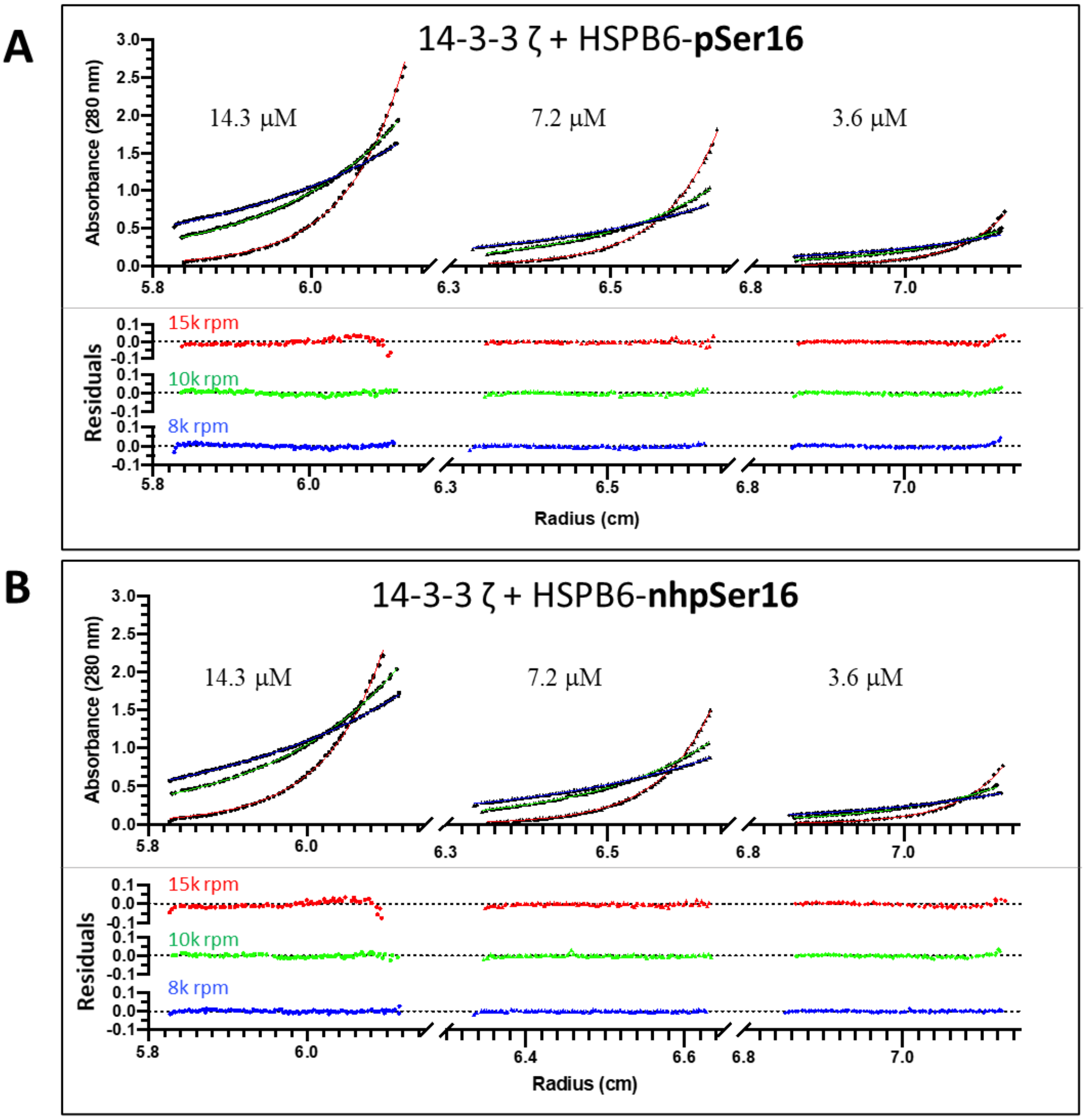

Figure S23. Equilibrium analytical ultracentrifugation analyses of the (A) pSer16- and (B) nhpSer16-HSPB6/14-3-3 complexes at 14.3 μM concentration (left column), 7.2 μM (middle column) and 3.6 μM (right column) at 15k (red), 10k (green) and 8k (blue) rpm. Residuals from fitting the equilibrium data to a two-state A+B <-->AB model are shown in the bottom row of each panel, in which the pSer and nhpSer complexes fit with dissociation constants of 92 ± 13 and 120 ± 26 nM (errors represent 95% confidence intervals).

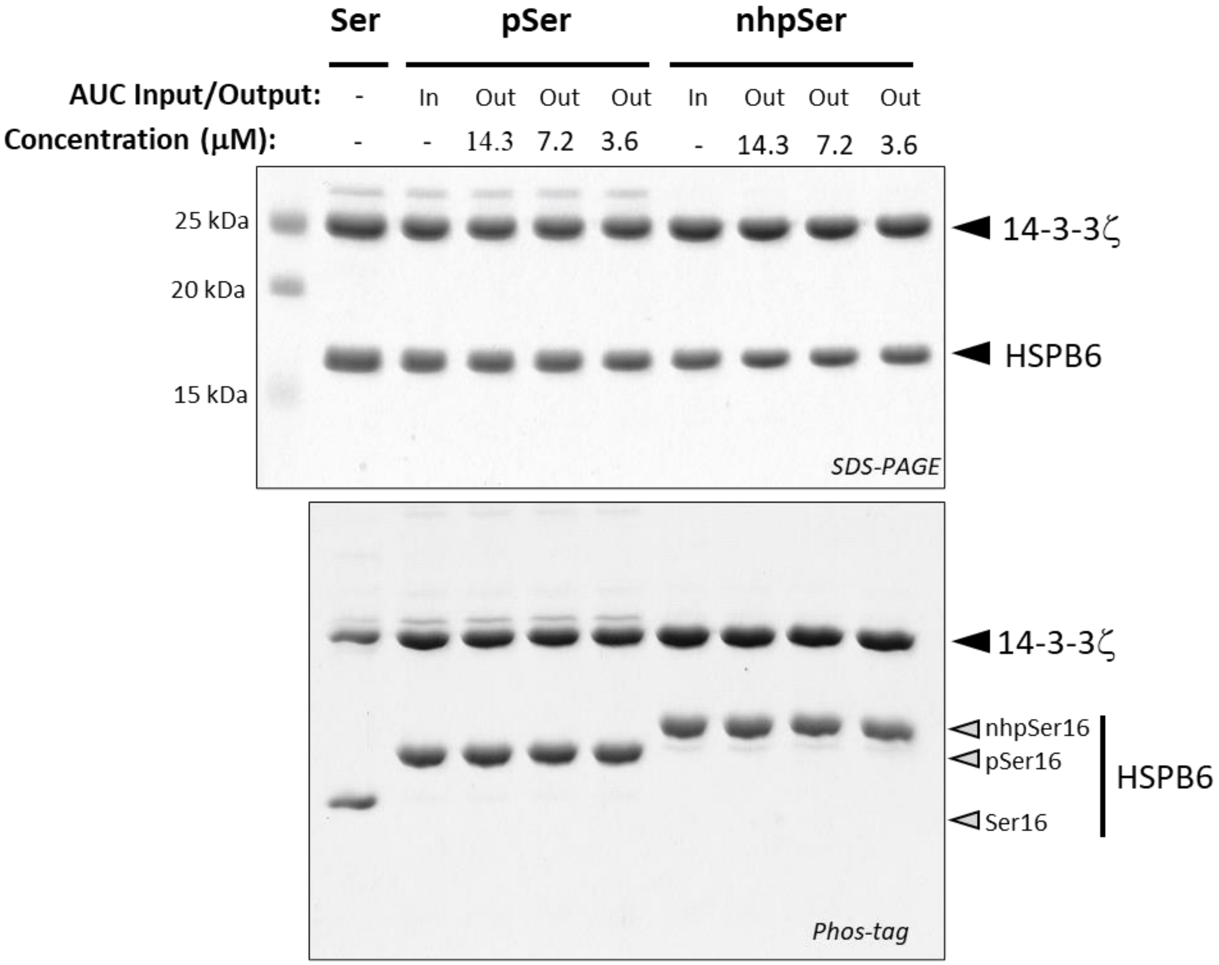

Figure S24. HSPB6/14-3-3ζ protein sample integrity before and after equilibrium analytical ultracentrifugation (AUC) analysis. Each sample shown in Supporting Fig. S23 was run after AUC analysis ("Out") along side the starting protein sample ("In"). A mixture of purified 14-3-3ζ and wild-type HSPB6 were loaded in the left lane for Phos-tag mobility comparison.

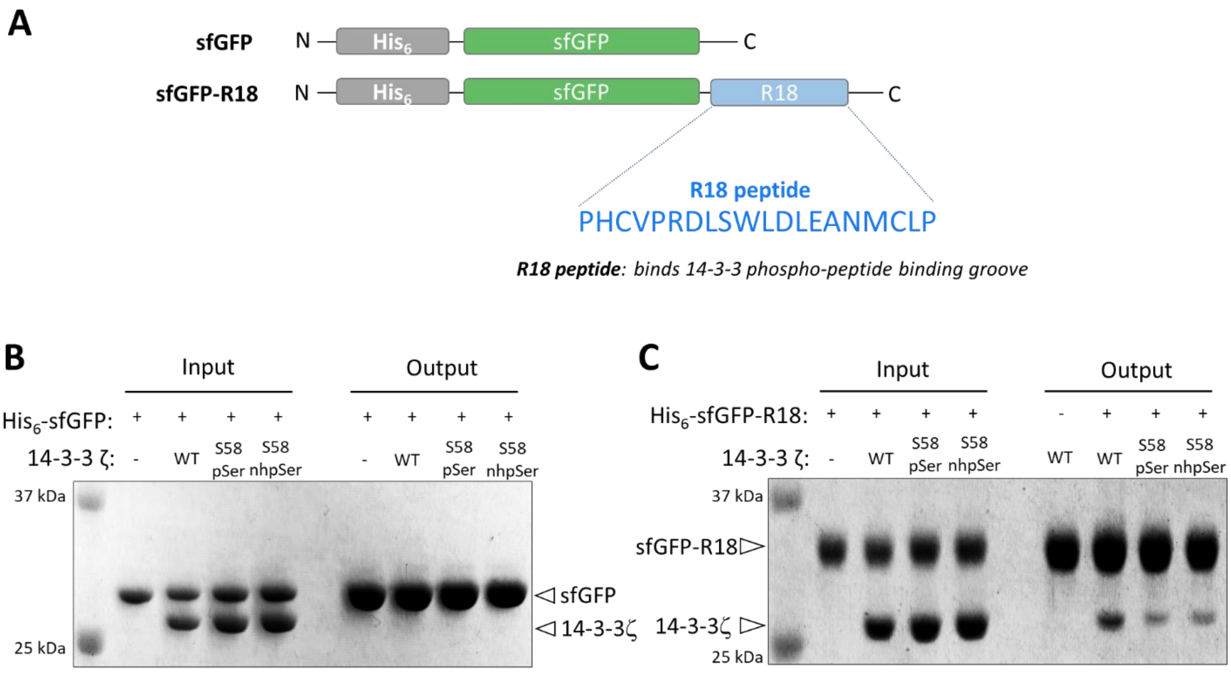

Figure S25. 14-3-3ζ phosphorylated at Ser58 with pSer and nhpSer retain a functional phospho-peptide binding groove. (A) Construct design for His$_6$-sfGFP and and His$_6$-sfGFP fused with R18; R18 is an engineered peptide that binds specifically to all 14-3-3 isoforms in the same amphipathic binding grove that phosphorylated peptides of 14-3-3 clients bind[14] (B) His$_6$-sfGFP and (C) His$_6$-sfGFP-R18 proteins were mixed with un-tagged 14-3-3ζ WT, pSer58 and nhpSer58 variants in equal molar ratios. Protein mixtures were then incubated with TALON metal affinity resin, which was washed extensively to remove unbound proteins. Bound proteins were eluted with imidazole and run on SDS-PAGE. When His$_6$-sfGFP was used as bait (panel B), none of the 14-3-3ζ forms co-eluted indicating no interaction as expected. When His$_6$-sfGFP-R18 was used as bait (panel C), 14-3-3ζ WT, pSer58 and nhpSer58 forms co-eluted, indicating a specific interaction between 14-3-3ζ and R18 in all cases, though the interaction of R18 with the pSer58 and nhpSer58 forms of 14-3-3ζ was weaker than with WT.

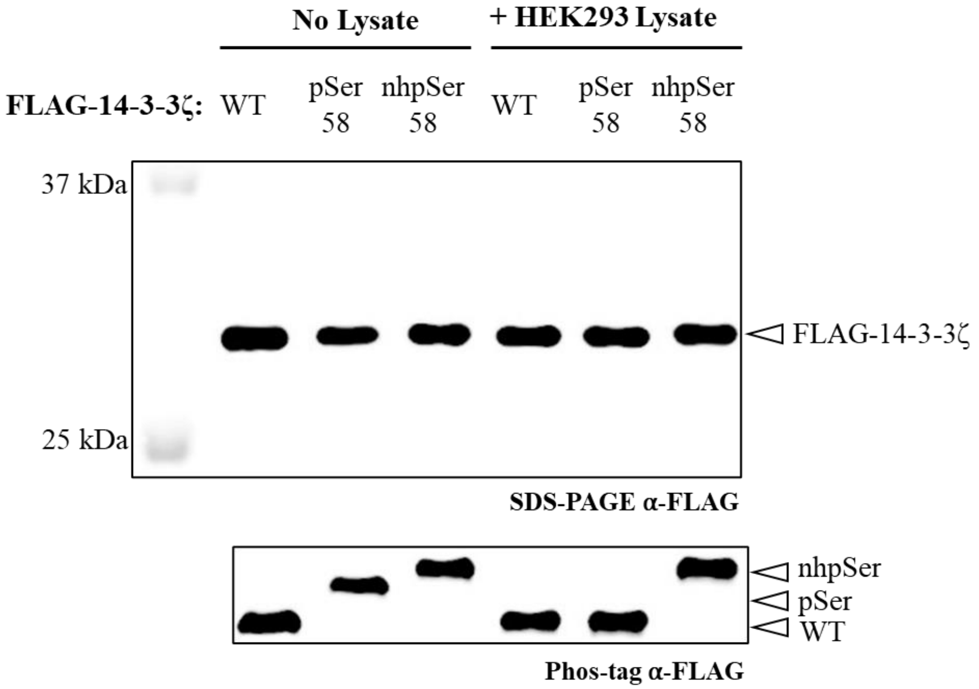

Figure S26. 14-3-3ζ pSer58 is hydrolyzed back to wild-type 14-3-3ζ in cell lysates. FLAG-tagged 14-3-3ζ WT, pSer58 and nhpSer58 expressed and purified from *E. coli* were incubated in buffer (no lysate) or HEK293T cell lysates for 120 min with calyculin A. Samples were run on SDS-PAGE (top) and Phos-tag (bottom) gels and probed with an α-FLAG antibody. Phos-tag electrophoresis confirmed the pSer58 variant was fully dephosphorylated in cell lysates during the course of incubation, while the nhpSer58 variant was unmodified.

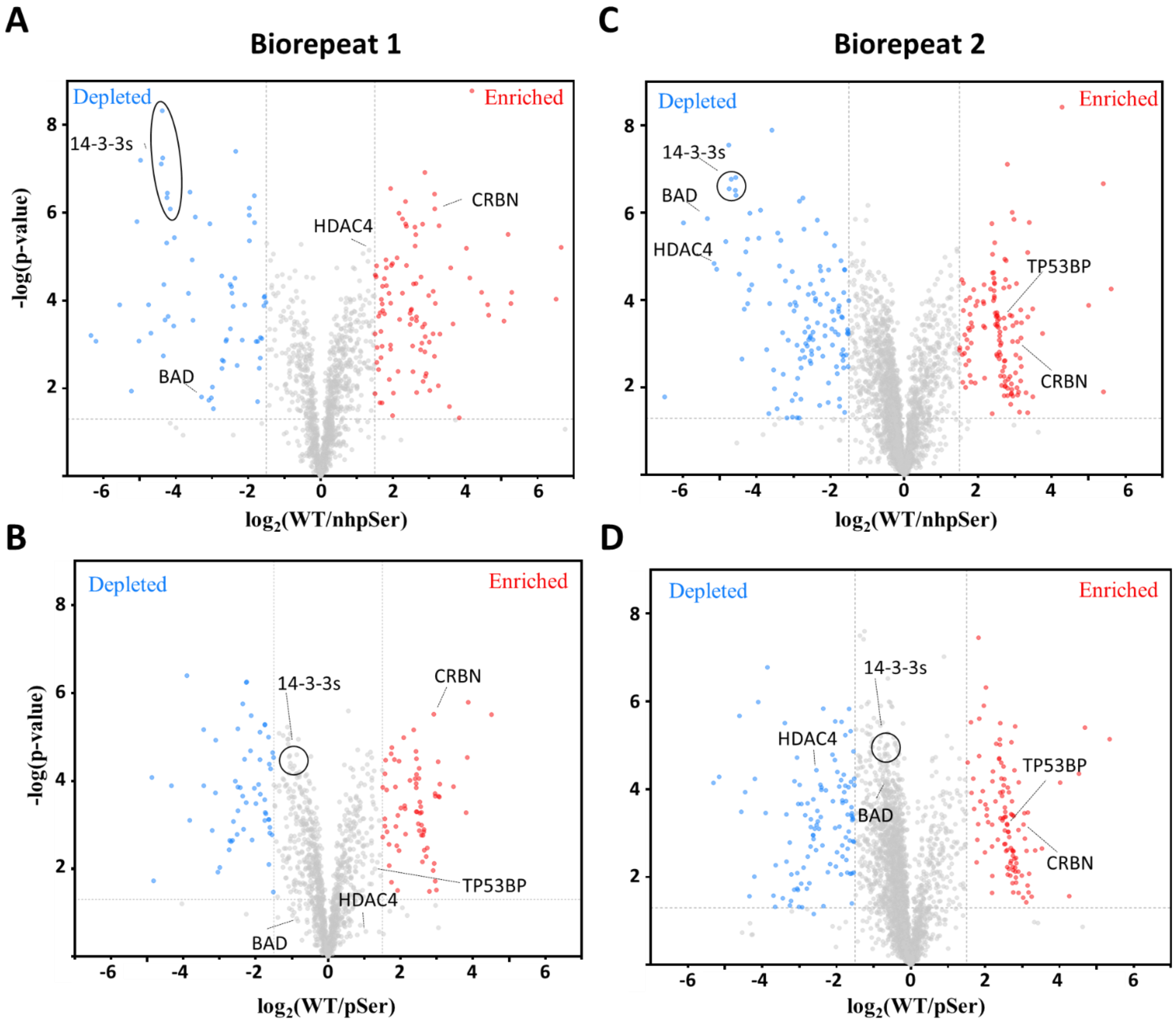

Figure S27. Volcano plots comparing the pool of proteins identified in two biological replicates for (A and C) 14-3-3ζ WT and nhpSer58 pulldowns and the (B and D) 14-3-3ζ WT and pSer58 pulldowns. Proteins identified in red as statistically "enriched" were at least 2.8-fold ($\log_2 1.5$) (p-value < 0.05) more abundant in the 14-3-3ζ nhpSer58 or pSer58 pools compared to that of 14-3-3ζ WT. Similarly, proteins identified in blue as statistically "depleted" were at least 2.8-fold ($\log_2 1.5$) (p-value < 0.05) less abundant in the 14-3-3ζ nhpSer58 or pSer58 pools compared to that of 14-3-3ζ WT. Protein IDs, enrichment values and associated p-values, as well as raw peptide intensities from both biological replicates, can be found in the Supporting Dataset 1.

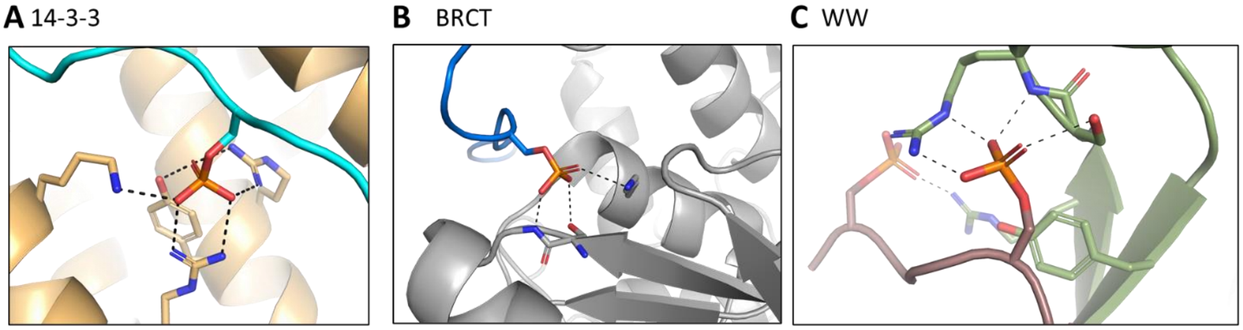

Figure S28. Prominent phospho-serine binding protein families do not use the bridging γ-oxygen as a recognition element. Representative structures of (A) 14-3-3 bound to a phosphoserine peptide (PDB 3mhr), (B) a BRCT domain bound to a phosphoserine peptide (PDB 3c0j) and (C) a WW domain bound to a doubly phosphorylated peptide (PDB 1f8a). Each case depicts the structural basis of phosphate-binding specificity for each domain but the pSer bridging γ-oxygen does not make a hydrogen bond with the pSer binding protein. Dotted black lines represent hydrogen bonds.

SUPPORTING SEQUENCES

>FrbA
MGRDLLRDLRLPDRNARYVSLAAAEEMGLVGISSAPRSLRVLVENTLRAAAAAKAGSTGDSLR
QAVLSANSLIAAATANGPVSEVEFRPTRLVLQDHSGIPVLADLASLRSIMADRGLDPRLARPALP
VDLVVDHSVEVASWARPDALSLNMRREYELNGERYRFLRWAQQEISGLRVVPPGRGIVHQMHL
EHLARVVLPDGDGLIAPDTVLGTDSHTPMVGALGVLGWGVGGIEAETVLLGHSVSLLSPQVIGV
RLTGELPVGSTATDLALTMTEFLRNVGVVGRFVEFFGPGVCALSVPDRATLSNMAPEYGCTVAF
FPVDDTTLRYLELTGRGAEEIRLVEAYCKEQGLFAGADASADPEFARVLDFDLSAVEPSAAGPRR
PQDRLPLGTVPASFPEPSGAALAGAAEGRRELADGAVAIAAITSCTNTANPHSMVTAGLVARKA
VQRGLAVPSWVKTSLAPGSRAVVHYLEAAGLLPDLEKLGFSVAGFGCTTCIGNSGGLAPEAEER
ARTEGVRLAAVLSGNRNFEGRIHPDVAAAYLASPALVVAYALAGTVLTDLTARPVGTDPDGAE
VFLADLWPTPEEIDAAVGLVAREHFASEKESLFTGGPEWTSLEHPSGPVYDWPAESDYLLRSPFF
DGLTHAPLSDLSGARVLVLTGDSTTTDHISPAGAIGADSPAGRYLRALGVEPALFNSYGCRRGNH
EVLVRGTFANPKFRNLLTPDVQGSSTLHVPTGERMSVHEAALAYAAEQVPVVVVGGRDYGFGS
SRDWAAKGPALLGVRAVLAKSFERIHRSNLIGMGIVPLEFLPDQDAGTLGLTGHEALDVIGLDGL
VPRGTVTVRARSAEGEPVAEWRMLARVDTAGELEYVRRGGFLRSVASDLLDAEA

>FrbB
MGTVSQYPDVAVRSDSPRPEASVLPAALELLESAGLTANWRIVAGQAPDGDGGRKPEAGLLPPF
AEPGASAYGLARAARRAWGGTFSVIERRGPLVRSDGTVNVLLFRYELDMSDTGMEFESNSGEQL
ALVKTAAELGPREIPEQAALGIQAIAPDRVSEFVRTAVGMCRRLDVDDLVVAHKGNSIKHTDGL
FALTAAETLESLAGAAGIAWRSLIIDHMAAELVRNPLDYRAILTHSSFADLLAAALAGQMTTDQL
GVCTTYDPAGRPLFGHDPALDTSAPDADPERWRVRRLASTLAAGAALAEHLRLPPAGRALRRA
AAELVRRDGGPIGWDQAVTTARKSLRAEGPARS

>FrbC
MGRNDLVLEDTTLRDGEQTPGVAFSKETKTAILNALIEAGVTSIEIGIPAMGGEELDFIKSVVDRQ
DEARLVVWHRGVREDVERSLDLGFTSVHVGLPTSAGHLKASVRKDRTWLLATARDMVKMAK
DRGAFVSISAEDIARTEISFLQEYAGVVAEAGADRLRLSDTVGLLGPEAYGERVAAVLSAADIDV
QCHAHNDFGLATANTLAGLKAGARYFHVTVNAIGERAGMADLAQVVVALKKLYDRDLGIDLT
KLKKVSRLVAEAAGHQVLPWQPITGDNVFAHESGIHANGMFRDTSSFEPFPPEHVGGERRYVLG
KHSGRALVAWALEQEGITPREELLPHCLEEVRALSIRIGGAVSHEQLVEIYNKAAA

>FrbD
MGTKRTMLRRAIARGGMSIAAGVHDGLSARIAQDAGFDVLWASGLGISAAHAVPDDSILTMTEF
LEAARVMNDSTDLPVLADCDTGFGDERNVARMVERYERAGIAGVCIEDKVFPKRNSLGDGAQD
QETIEGFAAKLAAAKKAQQTDDFVVVARIETFIAGGVKEDAIARADAYVAAGADAILIHSKSRD
GLEILDFGRSWQRKDVPLVAVPTTYPAVTADDLHEAGFSLAIYANQALRASITSMRESLRQITHD
RSSLRIEPKVAKLSEVFELQR

>FrbE
MGRKHTVTLIAGDGSGPELVAAARTVLDTAAGSAGFHLDWDVRPAGQGALRSVGELLPASTLD
SIRRNGVALKGPTTTPIGVGHRSVNITIRRELDLYACVRPVRVWPGAGAVDSGIDVVVVRENTED
VYSGLEYPADSDGAAVVSATLSEFGHADPGIAFSLKPITPGGSRRIVAFALDHARKNGRRKVEVG
ADVYRYPRTEGLFFEAARELGPEYPDIVTATVPVTVLCDSLVRDPADRDVLVLPNLYGDVISDVT

AALVGGLGMAPGANIGDRTAVFEATHGSAPEFAGSNRLNPTALILSGCMLLDHLGEAPTAARIR
AAVRSVLEEGRSVTFDLLPPDRAARAVTTTAYAEAVIERLRDIG

>sfGFP-His$_6$
MVSKGEELFTGVVPILVELDGDVNGHKFSVRGEGEGDATNGKLTLKFICTTGKLPVPWPTLVTT
LTYGVQCFSRYPDHMKRHDFFKSAMPEGYVQERTISFKDDGTYKTRAEVKFEGDTLVNRIELKG
IDFKE**D**GNILGHKLEYNFNSH**N**VYITADKQKNGIKANFKIRHNVEDGSVQLADHYQQNTPIGDGP
VLLPDNHYLSTQSVLSKDPNEKRDHMVLLEFVTAAGITHGMDELYKGS**HHHHH**
Note: D134 and N150 in bold/underline, black bold = His$_6$

>ΔN$_t$-bdSUMO-HSPB6[11-20]-sfGFP-His$_6$
M**AHINLKVKGQDGNEVFFRIKRSTQLKKLMNAYCDRQSVDMTAIAFLFDGRRLRAEQTPD**
ELEMEDGDEIDAMLHQTGGSG**WLRRASAPLP**GT**ENLYFQG**GSVSKGEELFTGVVPILVELDG
DVNGHKFSVRGEGEGDATNGKLTLKFICTTGKLPVPWPTLVTTLTYGVQCFSRYPDHMKRHDFF
KSAMPEGYVQERTISFKDDGTYKTRAEVKFEGDTLVNRIELKGIDFKEDGNILGHKLEYNFNSHN
VYITADKQKNGIKANFKIRHNVEDGSVQLADHYQQNTPIGDGPVLLPDNHYLSTQSVLSKDPNE
KRDHMVLLEFVTAAGITHGMDELYKGS**HHHHHH**
Note: blue = bdSUMO residues 19-97, gray = human HSPB6 residues 11-20, purple = TEV cut site, green = sfGFP, black bold = His$_6$, underlined = S16 of HSPB6.

>14-3-3ζ ΔC$_t$ (untagged in duet vector)
MDKNELVQKAKLAEQAERYDDMAACMKSVTEQGAELSNEERNLLSVAYKNVVGARRSSWRV
VSSIEQKTEGAEKKQQMAREYREKIETELRDICNDVLSLLEKFLIPNASQAESKVFYLKMKGDYY
RYLAEVAAGDDKKGIVDQSQQAYQEAFEISKKEMQPTHPIRLGLALNFSVFYYEILNSPEKACSL
AKTAFDEAIAELDTLSEESYKDSTLIMQLLRDNLTLWT

>His$_6$-bdSUMO-14-3-3ζ ΔC$_t$
MGSS**HHHHHH**SGSA**AGGEEDKKPAGGEGGGAHINLKVKGQDGNEVFFRIKRSTQLKKLM**
NAYCDRQSVDMTAIAFLFDGRRLRAEQTPDELEMEDGDEIDAMLHQTGGSGMDKNELVQK
AKLAEQAERYDDMAACMKSVTEQGAELSNEERNLLSVAYKNVVGARRS**S**WRVVSSIEQKTEG
AEKKQQMAREYREKIETELRDICNDVLSLLEKFLIPNASQAESKVFYLKMKGDYYRYLAEVAAG
DDKKGIVDQSQQAYQEAFEISKKEMQPTHPIRLGLALNFSVFYYEILNSPEKACSLAKTAFDEAIA
ELDTLSEESYKDSTLIMQLLRDNLTLWT
Note: blue = bdSUMO residues 2-97, underlined = S58 of 14-3-3ζ

>His$_6$-bdSUMO-HSPB6
MGSS**HHHHHH**SG**SAAGGEEDKKPAGGEGGGAHINLKVKGQDGNEVFFRIKRSTQLKKLM**
NAYCDRQSVDMTAIAFLFDGRRLRAEQTPDELEMEDGDEIDAMLHQTGGSG**EIPVPVQPS**
WLRRASAPLPGLSAPGRLFDQRFGEGLLEAELAALCPTTLAPYYLRAPSVALPVAQVPTDP
GHFSVLLDVKHFSPEEIAVKVVGEHVEVHARHEERPDEHGFVAREFHRRYRLPPGVDPAAV
TSALSPEGVLSIQAAPASAQA
Note: blue = bdSUMO residues 2-97, gray = Human HSPB6 2-153, black bold = His$_6$, underlined = S16

>His$_6$-bdSUMO-SARS-CoV-2 Nucleocapsid protein (full-length)
MGSS**HHHHHH**SGS**AHINLKVKGQDGNEVFFRIKRSTQLKKLMNAYADRQSVDMTALAFLF**
DGRRLRAEQTPDELEMEDGDEIDAMLHQTGGSGSDNGPQNQRNAPRITFGGPSDSTGSNQNG
ERSGARSKQRRPQGLPNNTASWFTALTQHGKEDLKFPRGQGVPINTNSSPDDQIGYYRRATRRIR

GGDGKMKDLSPRWYFYYLGTGPEAGLPYGANKDGIIWVATEGALNTPKDHIGTRNPANNAAIV
LQLPQGTTLPKGFYAEGSRGGSQASSRSSSRSRNSSRNSTPGSSRGTSPARMAGNGGDAALALLL
LDRLNQLESKMSGKGQQQQGQTVTKKSAAEASKKPRQKRTATKAYNVTQAFGRRGPEQTQGN
FGDQELIRQGTDYKHWPQIAQFAPSASAFFGMSRIGMEVTPSGTWLTYTGAIKLDDKDPNFKDQ
VILLNKHIDAYKTFPPTEPKKDKKKKADETQALPQRQKKQQTVTLLPAADLDDFSKQLQQSMSS
ADSTQA

Note: blue = bdSUMO residues 19-97, orange = Np full-length, purple = TEV cut site, black bold = His$_6$, underlined = S188 and S206 of the Np.

>ΔN_t-bdSUMO-Np Linker [175-247]-sfGFP-His$_6$

M**AHINLKVKGQDGNEVFFRIKRSTQLKKLMNAYCDRQSVDMTAIAFLFDGRRLRAEQTPD
ELEMEDGDEIDAMLHQTGG**S**GSRGGSQASSRSSSRSRNSSRNSTPGSSRGTSPARMAGNGG
DAALALLLLDRLNQLESKMSGKGQQQQGQT**GT**ENLYFQG**GSVSKGEELFTGVVPILVELDG
DVNGHKFSVRGEGEGDATNGKLTLKFICTTGKLPVPWPTLVTTLTYGVQCFSRYPDHMKRHDFF
KSAMPEGYVQERTISFKDDGTYKTRAEVKFEGDTLVNRIELKGIDFKEDGNILGHKLEYNFNSHN
VYITADKQKNGIKANFKIRHNVEDGSVQLADHYQQNTPIGDGPVLLPDNHYLSTQSVLSKDPNE
KRDHMVLLEFVTAAGITHGMDELYKGS**HHHHHH**

Note: blue = bdSUMO residues 19-97, orange = Np linker residues 175-247, purple = TEV cut site, green = sfGFP, black bold = His$_6$, underlined = S188 and S206 of the Np.

>His$_6$-TEV-bdSENP1

MGSS**HHHHHH**SS**ENLYFQG**GKEEVPEPFVPLTDEDEDNVRHALGGRKRSETLSVHEASNIVITR
EILQCLNDKEWLNDEVINLYLELLKERELREPNKFLKCHFFNTFFYKKLINGGYDYKSVRRWTTK
RKLGYNLIDCDKIFVPIHKDVHWCLAVINIKEKKFQYLDSLGYMDMKALRILAKYLVDEVKDKS
GKQIDVHAWKQEGVQNLPLQENGWDCGMFMLKYIDFYSRDMELVFGQKHMSYFRRRTAKEIL
DLKAG

Note: red = bdSENP1 residues 242-481, black bold = His$_6$, purple = TEV cut site

>His$_6$-ΔN_t- bdSUMO-GSK3β S9A

MGSS**HHHHHH**SGS**AHINLKVKGQDGNEVFFRIKRSTQLKKLMNAYCDRQSVDMTAIAFLF
DGRRLRAEQTPDELEMEDGDEIDAMLHQTGG**SGSGRPRTT**A**FAESCKPVQQPSAFGSMKVSR
DKDGSKVTTVVATPGQGPDRPQEVSYTDTKVIGNGSFGVVYQAKLCDSGELVAIKKVLQDKRF
KNRELQIMRKLDHCNIVRLRYFFYSSGEKKDEVYLNLVLDYVPETVYRVARHYSRAKQTLPVIY
VKLYMYQLFRSLAYIHSFGICHRDIKPQNLLLDPDTAVLKLCDFGSAKQLVRGEPNVSYICSRYY
RAPELIFGATDYTSSIDVWSAGCVLAELLLGQPIFPGDSGVDQLVEIIKVLGTPTREQIREMNPNYT
EFKFPQIKAHPWTKVFRPRTPPEAIALCSRLLEYTPTARLTPLEACAHSFFDELRDPNVKLPNGRD
TPALFNFTTQELSSNPPLATILIPPHARIQAAASTPTNATAASDANTGDRGQTNNAASASASNST

Note: blue = bdSUMO residues 19-97, yellow = GSK3β (human), black bold = His$_6$, underlined = S9A mutation

Primers Table: Primers used for generating the FrbABCDE library

Making T7 promoter library

P1 ACCTGTGGCGCCGGTGATGC
P2 TAGAGGGGAATTGTTATCCGCTCACAATTCNNNNNNAGTGAGTCGTATTAATTTCGC
P3 GAATTGTGAGCGGATAACAATTCCCCTCT
P4 TTCCACCAGAATCGGCACAACGC

Making fragments for FrbABCDE library

CDF-SpecR

P5 ttatggcGGTCTCaatctCACACGGTCACACTGCTTCCGG
P6 actggagaGGTCTCgccagAGGGAGAGCGTCGAGATCCC

FrbA (orf1)

P7 cgGGTCTCtCTGGcgtctcaacacgaaattaatacgactcactataggggaattgtgagc
P8 cgGGTCTCtCTGGcgtctcaacacgaaattaatacgactcactaaggcggaattgtgagc
P9 cgGGTCTCtCTGGcgtctcaacacgaaattaatacgactcactctaccagaattgtgagc
P10 cgGGTCTCtCTGGcgtctcaacacgaaattaatacgactcactattattgaattgtgagc
P11 cgGGTCTCtCTGGcgtctcaacacgaaattaatacgactcacttttgaccgaattgtgagc
P12 cttgagGGTCTCaACTCcgtctcacgaactcctttcagcaaaaaacccctcaag

FrbB (orf2)

P13 cgGGTCTCtGAGTcgtctcaacacgaaattaatacgactcactataggggaattgtgagc
P14 cgGGTCTCtGAGTcgtctcaacacgaaattaatacgactcactaaggcggaattgtgagc
P15 cgGGTCTCtGAGTcgtctcaacacgaaattaatacgactcactctaccagaattgtgagc
P16 cgGGTCTCtGAGTcgtctcaacacgaaattaatacgactcactattattgaattgtgagc
P17 cgGGTCTCtGAGTcgtctcaacacgaaattaatacgactcacttttgaccgaattgtgagc
P18 cttgagGGTCTCaAGCGcgtctcacgaactcctttcagcaaaaaacccctcaag

FrbC (orf3)

P19 cgGGTCTCtCGCTcgtctcaacacgaaattaatacgactcactataggggaattgtgagc
P20 cgGGTCTCtCGCTcgtctcaacacgaaattaatacgactcactaaggcggaattgtgagc
P21 cgGGTCTCtCGCTcgtctcaacacgaaattaatacgactcactctaccagaattgtgagc
P22 cgGGTCTCtCGCTcgtctcaacacgaaattaatacgactcactattattgaattgtgagc
P23 cgGGTCTCtCGCTcgtctcaacacgaaattaatacgactcacttttgaccgaattgtgagc
P24 cttgagGGTCTCaATGAcgtctcacgaactcctttcagcaaaaaacccctcaag

FrbD (orf4)

P25 cgGGTCTCtTCATcgtctcaacacgaaattaatacgactcactataggggaattgtgagc
P26 cgGGTCTCtTCATcgtctcaacacgaaattaatacgactcactaaggcggaattgtgagc
P27 cgGGTCTCtTCATcgtctcaacacgaaattaatacgactcactctaccagaattgtgagc
P28 cgGGTCTCtTCATcgtctcaacacgaaattaatacgactcactattattgaattgtgagc
P29 cgGGTCTCtTCATcgtctcaacacgaaattaatacgactcacttttgaccgaattgtgagc
P30 cttgagGGTCTCaTCCAcgtctcacgaactcctttcagcaaaaaacccctcaag

FrbE (orf5)

P31 cgGGTCTCtTGGAcgtctcaacacgaaattaatacgactcactataggggaattgtgagc

P32 cgGGTCTCtTGGAcgtctcaacacgaaattaatacgactcactaaggcggaattgtgagc

P33 cgGGTCTCtTGGAcgtctcaacacgaaattaatacgactcactctaccagaattgtgagc

P34 cgGGTCTCtTGGAcgtctcaacacgaaattaatacgactcactattattgaattgtgagc

P35 cgGGTCTCtTGGAcgtctcaacacgaaattaatacgactcacttgaccgaattgtgagc

P36 cttgagGGTCTCaAGATcgtctcacgaactcctttcagcaaaaaacccctcaag

REFERENCES

1. Freestone, T. S.; Zhao, H., Combinatorial pathway engineering for optimized production of the anti-malarial FR900098. *Biotechnol Bioeng* **2016,** *113* (2), 384-92.
2. Zhang, Y.; Werling, U.; Edelmann, W., SLiCE: a novel bacterial cell extract-based DNA cloning method. *Nucleic Acids Res* **2012,** *40* (8), e55.
3. Zhu, P.; Gafken, P. R.; Mehl, R. A.; Cooley, R. B., A Highly Versatile Expression System for the Production of Multiply Phosphorylated Proteins. *ACS Chem Biol* **2019,** *14* (7), 1564-1572.
4. Rogerson, D. T.; Sachdeva, A.; Wang, K.; Haq, T.; Kazlauskaite, A.; Hancock, S. M.; Huguenin-Dezot, N.; Muqit, M. M.; Fry, A. M.; Bayliss, R.; Chin, J. W., Efficient genetic encoding of phosphoserine and its nonhydrolyzable analog. *Nat Chem Biol* **2015,** *11* (7), 496-503.
5. Zhang, M. S.; Brunner, S. F.; Huguenin-Dezot, N.; Liang, A. D.; Schmied, W. H.; Rogerson, D. T.; Chin, J. W., Biosynthesis and genetic encoding of phosphothreonine through parallel selection and deep sequencing. *Nat Methods* **2017,** *14* (7), 729-736.
6. Park, H. S.; Hohn, M. J.; Umehara, T.; Guo, L. T.; Osborne, E. M.; Benner, J.; Noren, C. J.; Rinehart, J.; Soll, D., Expanding the genetic code of Escherichia coli with phosphoserine. *Science* **2011,** *333* (6046), 1151-4.
7. Mukai, T.; Hoshi, H.; Ohtake, K.; Takahashi, M.; Yamaguchi, A.; Hayashi, A.; Yokoyama, S.; Sakamoto, K., Highly reproductive Escherichia coli cells with no specific assignment to the UAG codon. *Sci Rep* **2015,** *5*, 9699.
8. Frey, S.; Gorlich, D., A new set of highly efficient, tag-cleaving proteases for purifying recombinant proteins. *J Chromatogr A* **2014,** *1337*, 95-105.
9. Frey, S.; Gorlich, D., Purification of protein complexes of defined subunit stoichiometry using a set of orthogonal, tag-cleaving proteases. *J Chromatogr A* **2014,** *1337*, 106-15.
10. Tugaeva, K. V.; Hawkins, D.; Smith, J. L. R.; Bayfield, O. W.; Ker, D. S.; Sysoev, A. A.; Klychnikov, O. I.; Antson, A. A.; Sluchanko, N. N., The Mechanism of SARS-CoV-2 Nucleocapsid Protein Recognition by the Human 14-3-3 Proteins. *J Mol Biol* **2021,** *433* (8), 166875.
11. Hornbeck, P. V.; Zhang, B.; Murray, B.; Kornhauser, J. M.; Latham, V.; Skrzypek, E., PhosphoSitePlus, 2014: mutations, PTMs and recalibrations. *Nucleic Acids Res* **2015,** *43* (Database issue), D512-20.
12. Kinoshita, E.; Kinoshita-Kikuta, E.; Takiyama, K.; Koike, T., Phosphate-binding tag, a new tool to visualize phosphorylated proteins. *Mol Cell Proteomics* **2006,** *5* (4), 749-57.
13. Beranek, V.; Reinkemeier, C. D.; Zhang, M. S.; Liang, A. D.; Kym, G.; Chin, J. W., Genetically Encoded Protein Phosphorylation in Mammalian Cells. *Cell Chem Biol* **2018,** *25* (9), 1067-1074 e5.
14. Wang, B.; Yang, H.; Liu, Y. C.; Jelinek, T.; Zhang, L.; Ruoslahti, E.; Fu, H., Isolation of high-affinity peptide antagonists of 14-3-3 proteins by phage display. *Biochemistry* **1999,** *38* (38), 12499-504.
